# Humanized yeast ORC drives R-loop dependent transcription-replication conflicts and global gene dysregulation

**DOI:** 10.1038/s41467-026-79167-4

**Published:** 2026-11-13

**Authors:** Clare S. K. Lee, Maxime Lalonde, Tobias Straub, Myriam Zheng, Elizabeth Márquez-Gómez, Melina Riepl, Bozhao Dong, Ioannis Tsirkas, Atiqa Sajid, Marcel Werner, Ana Janeva, Xanthoula Karypidou, Antonio Scialdone, Kurt M. Schmoller, Benoit Palancade, Bik Kwoon Tye, Stephan Hamperl

**Affiliations:** 1 Chromosome Dynamics and Genome Stability, Institute of Epigenetics and Stem Cells, Helmholtz Munich, Feodor-Lynen-Strasse 21, München, Germany; 2 Core Facility Bioinformatics, Biomedical Center, Faculty of Medicine, Ludwig-Maximilians-Universität München, Martinsried, Germany; 3 Université Paris Cité, CNRS, Institut Jacques Monod, Paris, France; 4 Institute of Computational Biology, Helmholtz Zentrum München, Neuherberg, Germany; 5 Institute of Functional Epigenetics, Molecular Targets and Therapeutics Center, Helmholtz Zentrum München, Neuherberg, Germany; 6 Department of Molecular Biology and Genetics, Cornell University, Ithaca, NY USA

**Keywords:** DNA, Origin firing, Transcriptional regulatory elements, Replisome

## Abstract

The Origin Recognition Complex (ORC) recruits Mini-Chromosome Maintenance (MCM) hexamers to initiate eukaryotic DNA replication. While yeast ORC binds specific DNA motifs, human ORC binds sequence-agnostically nucleosome-depleted promoter regions. Here, using a mutant yeast model that recapitulates the human ORC binding landscape, we demonstrate that pervasive and unscheduled ORC binding at promoters in the compact yeast genome generates R-loop associated transcription-replication conflicts and disrupts gene expression across thousands of genes. Unexpectedly, we also uncover that substituting endogenous gene promoters with a replication origin sequence maintains robust transcription. We reveal that this intrinsic, promoter-like function relies on the ability of origins to form a nucleosome-depleted region (NDR) encoded within the origin’s sequence composition. However, origin-substituted loci reposition the +1 nucleosome which drives the transcription machinery to select alternative transcription start sites (TSSs). Thus, origins and promoters share an intrinsic chromatin structure-controlled ability to drive transcription initiation. Together, our results show that origin licensing in compact genomes relies on sequence-specific, coupled origin licensing to insulate transcription from the replication machinery.

## Abstract (web summary)

ORC binds specific sites in yeast but broad promoter regions in humans, and whether origins and promoters share functions is unclear. Here, the authors use a humanized yeast model to show that ORC-bound origins trigger transcription-replication conflicts and can themselves act as promoters.

## Introduction

The Origin Recognition Complex (ORC) is a conserved six-subunit protein complex that binds genomic regions and marks them as potential DNA replication initiation sites. ORC recruits Mini-Chromosome Maintenance (MCM) double hexamers in a head-to-head conformation, forming the pre-replicative complex (pre-RC) responsible for DNA unwinding during replication. Although the proteins are structurally conserved, ORC binding site selectivity varies significantly among species, from DNA sequence-specific binding in budding yeast to no known consensus sequence requirement in higher eukaryotes. In mammals, genomic locations of ORC binding frequently correlate with different genomic features, such as CpG island containing gene promoters, with so-called “origin G-rich Repeated Element” (OGRE) motifs, G quadruplexes and other chromatin features that include open chromatin and certain histone modifications that represent active chromatin such as H3K27ac, H3K9ac, H3K4me2, H3K4me1, and H3K4me3^1–9^. In the budding yeast *Saccharomyces cerevisiae*, DNA replication is initiated from defined locations known as Autonomously Replication Sequences (ARSs). Canonical ARSs are characterized by a conserved AT-rich 11 bp stretch known as the ARS Consensus Sequence (ACS), which is essential for binding of the ORC complex. Notably, an additional B1 element consisting of WTW nucleotides from 17 to 19 bp 3′ to the ACS T-rich strand serves as the primary ORC binding site in yeast^10,11^. Yeast ORC binding specificity to the ACS is determined by a 19-amino acid insertion helix (IH) within the Orc4 subunit^12^. Removal of this IH motif (ORC-IHΔ) transforms yeast ORC from a sequence-specific DNA-binding to an agnostic binding to nucleosome-depleted regions (NDRs), which are commonly found at gene promoters^13^. Because promoter NDRs structurally resemble  budding yeast ARSs, being AT-rich and flanked by well-positioned −1 and +1 nucleosomes, ORC-IHΔ retains its preference for open chromatin architecture despite losing sequence specificity, mimicking the motif-independent binding pattern of human ORC.

In yeast, there are two proposed mechanisms explaining how ORC loads MCM double hexamers at ARSs. At canonical ARSs, ORC first binds at the ACS element, recruits the first MCM hexamer, then flip over to the B2 site and recruit the second MCM hexamer^14^. At non-canonical ARSs, two separate ORC complexes, each bound to one of the sites (ACS and B1), load the two MCM hexamers independently but in close proximity^15^. In either scenario, ORC DNA binding and MCM loading are coupled, meaning that the final head-to-head MCM double hexamer is formed at ACS where ORC binds, marking the sites of replication initiation.

This process is likely different in humans because human replication origins do not show precise and closely spaced ORC binding sites. Without a second nearby binding site, ORC can only load one single MCM hexamer at its location. Therefore, a head-to-head double hexamer is thought to form when two single hexamers, loaded at different locations, move toward each other and collide in a correct N- to N- terminal orientation^16,17^. Consistently, in the case of the Orc4-IHΔ mutant, ORC-IHΔ binding at promoters will likely result in single hexamer loading due to the absence of B elements, despite the fact that in vitro tests showed similar efficiency in pre-RC assembly as the wild-type^13^. This suggests that the humanized Orc4-IHΔ mutant may also load MCM at distinct promoters as single hexamers, which are then in a second step brought together to form stable MCM double hexamers.

Previous work has shown that RNA polymerases can push MCM hexamers in the direction of transcription^18^, making transcription an attractive candidate genomic process responsible to drive single MCM hexamers together in humanized Orc4-IHΔ. If transcription is responsible for the relocalization and the assembly of double hexamers, this would raise immediate questions: Are the sites of MCM double hexamer formation randomly distributed, or do they show a preferred localization following the orientation of transcription? Would the mislocalized MCM complexes constitute obstacles to transcription, and would they interfere with the gene expression program? Could the relocated MCM double hexamers be activated in S phase and lead to genome-destabilizing Transcription-Replication Conflicts (TRCs)? These questions remain largely unanswered, yet they hold significant impact on gene expression maintenance and genome stability.

In this study, we investigated the transcriptional consequences of altered genome-wide ORC-IHΔ binding at promoters. Strikingly, the “humanized” yeast ORC binding at promoters resulted in pre-RC formation at the 3’ end of the gene bodies, which in turn led to increased transcription-replication conflicts (TRCs) and accumulation of RNA-DNA hybrids. RNA-Seq analyses revealed widespread gene expression changes throughout the cell cycle in the Orc4-IHΔ mutant, most notably the upregulation of stress response genes and the downregulation of ribosome biogenesis. Crucially, overexpressing RNase H1 partially rescued these transcriptional defects, indicating that R-loop accumulation directly contributes to the observed expression dysregulation. We further investigated the direct effect of ORC binding at promoters on transcription by substituting the native promoters of four candidate genes with the ARS1 replication origin sequence. Our results show that the replacement of the NDR of native promoters with the ARS1 sequence maintains transcription, however with altered transcription start sites dictated by the repositioned location of the + 1 nucleosome. These findings indicate an inherent promoter-like function of yeast replication origins.

More generally, the “humanized” yeast system provides a valuable and easy to manipulate model with immediate relevance to higher eukaryotic systems and can explain why compact and gene-dense genomes such as that of yeast make use of sequence-specific and coupled ORC and MCM loading to improve coordination between transcription and replication machineries and to avoid conflicts leading to replication stress and associated genomic instability.

## Results

### Pervasive ORC-IHΔ binding at promoters in Orc4-IHΔ induces TRCs and R-loop dependent cell cycle arrest

**Fig. 1:**
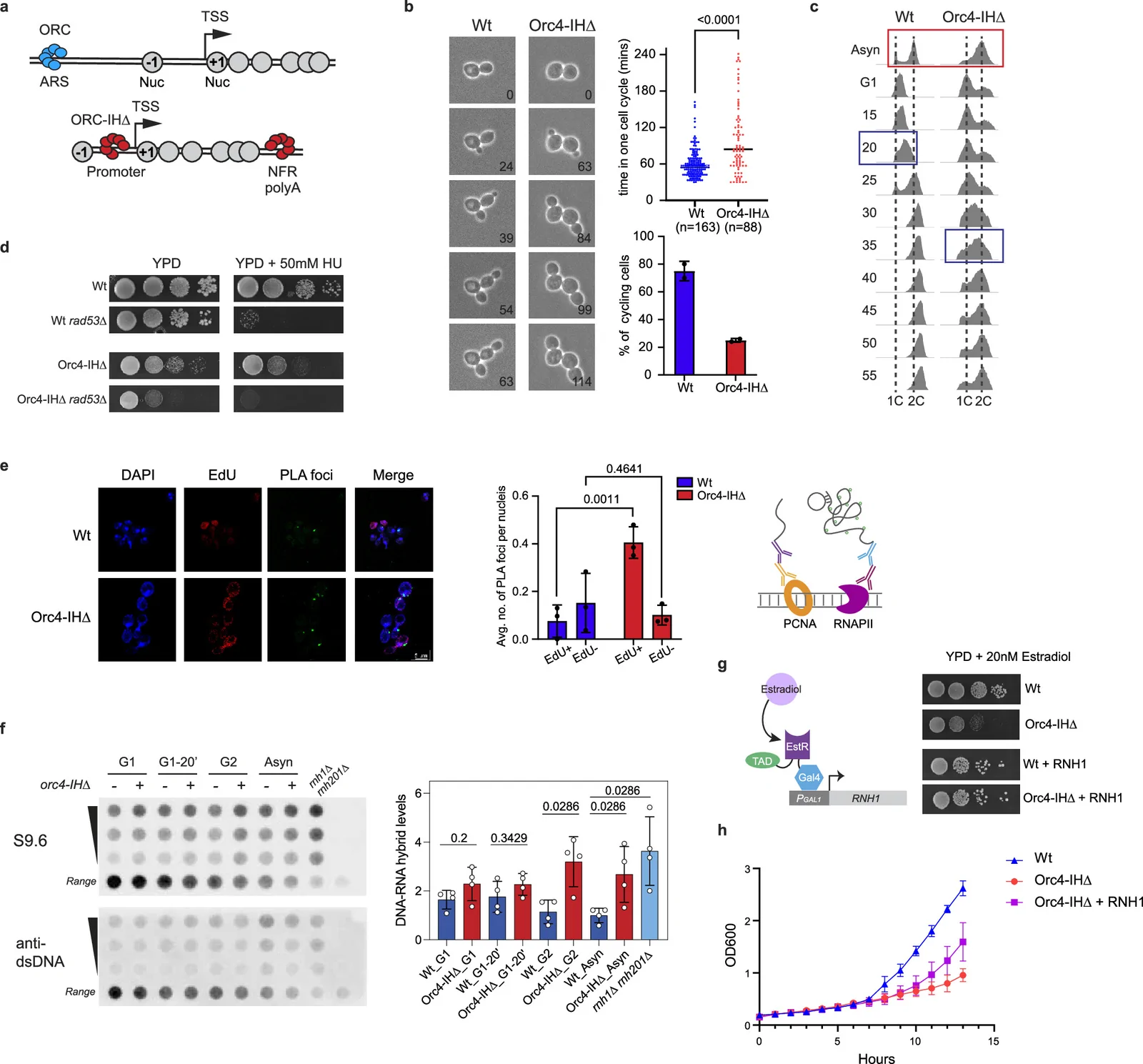
Pervasive ORC-IHΔ binding at promoters in Orc4-IHΔ induces R-loop dependent replication stress and TRCs. **a** Cartoon showing Wt and mutant ORC-IHΔ binding patterns in the genome. **b** Left panel: Representative time-lapse microscopic images of single-cell tracking of Wt and Orc4-IHΔ using the Cell-ACDC platform. The number at the bottom right indicates minutes. Right panel: Quantification of time spent in one cell cycle of each tracked cell (top) and percentage of cycling cells in Wt and Orc4-IHΔ (bottom). Unpaired t-test, two-sided was performed on the quantification of time spent in one cell cycle. The percentage of cycling cells were probably underestimated in both cell types, as we stopped video recording and/or tracking errors when the fields of view were crowded with growing cells. Error bars indicate mean values with standard deviations (SD). Plots shown are representative of *n*  =  3 biological replicates. **c** FACS analysis shows Orc4-IHΔ cells exhibit a delay in cell cycle progression. Wt and mutant cells were first arrested in G1 using alpha factor and then released into S phase (number on the left indicates minutes release from G1). **d** HU sensitivity of Rad53 depletion in Wt and Orc4-IHΔ is assayed by spotting serially diluted WT and mutant cells onto YPD plates containing 0 or 150 mM of HU. **e** Left panel: Representative images of Proximity Ligation Assay (PLA) assay with RNAPII Ser2P and PCNA antibodies in Wt and Orc4-IHΔ cells. EdU click-it staining was performed to label S phase cells. The nuclei of the asynchronously growing cell population were stained with DAPI. Middle panel: Quantification of PLA foci number in S phase cells (EdU + ) and non-S phase cells (EdU-) in Wt and Orc4-IHΔ (*n* = 3). Bars indicate mean values with standard deviations (SD). Two-way ANOVA with multiple comparisons test. Right panel: Diagram showing the PLA reaction using two antibodies of different species to label the replication and transcription machinery. **f** Dot blot assay showing the blot images (left panel) and quantitative analysis (right panel) of R-loops in the Wt and Orc4-IHΔ at indicated cell cycle stages (Asyn:asynchronous phase; G1:G1 phase; G1_20: 20 mins post release from G1, early S phase; G2M phase). R-loop signals were normalized to the total amount of dsDNA in the genomic DNA extracts. The *rnh1∆ rnh201∆* strain was used as an R-loop-forming positive control. Error bars indicate mean values with standard deviations (SD). Mann-Whitney-Wilcoxon rank-sum test was performed. Blots and plots shown are representative of *n*  =  3 biological replicates. **g** Diagram showing the estradiol induction system for the overexpression of *RNH1* and spot test. Left panel: A chimeric transcription factor which is composed of the Gal4 DNA-binding domain and the ligand-binding domain of the human Estrogen Receptor (EstR), as well as a transcription activation domain (TAD), drives the overexpression of *RNH1* by the *GAL1* promoter in the presence of estradiol. Right panel: Effect on growth phenotype under overexpression of *RNH1* is assayed by spotting serially diluted Wt and Orc4-IHΔ yeast strains alone or over-expressing Rnh1 on YPD plates containing 20 nM estradiol. **h** Growth analysis of Wt (blue), Orc4-IHΔ (red), and Orc4-IHΔ overexpressing Rnh1 (purple) in YPD, growth was monitored for 13 h and OD600 absorbance were recorded once every hour. All source data associated with this figure are provided in the Source Data file.

Our previous study showed that mutant ORC-IHΔ preferentially relocates from ARSs to nucleosome-depleted promoter regions, which account for ~80% of the de novo mutant ORC-IHΔ binding sites (Fig. 1a and Supplementary Fig. 1a)^13^. To characterize the functional consequences of this pervasive ORC-IHΔ binding on cell cycle, proliferation and cellular fitness, we first employed time-lapse single-cell microscopy^19^ to follow cell growth and divisions in individual cells over multiple cell cycles. We tracked wildtype (Wt) and Orc4-IHΔ cells and measured the time needed for each tracked cell to complete one cell cycle from bud emergence to separation of mother and daughter cell. Compared to Wt, Orc4-IHΔ cells require significantly more time to complete one cell cycle (Fig. 1b, right upper panel). In addition, only about 25% of mutant cells show bud emergence and active cell division across two generations, whereas more than 75% of Wt cells were actively cycling and dividing over at least two cell cycles (Fig. 1b, right lower panel), suggesting a severe cell cycle defect in Orc4-IHΔ cells. To determine which cell cycle stage is affected, we measured DNA content by flow cytometry. Asynchronous (Asyn) Orc4-IHΔ cells show a reduced number of G1 phase cells with 1 N DNA content compared to Wt and a much broader G2/M peak with 2 N DNA content, indicating a cell cycle defect in late S or G2/M phases (Fig. 1c, red box). Consistently, upon release from alpha factor-mediated G1 arrest, Orc4-IHΔ cells show a delay in S phase progression by about 10-15 min to reach a similar DNA content of mid S phase as Wt cells (Fig. 1c, blue boxes). In our previous study, we did not capture a significant delay in S-phase progression for the Orc4-IHΔ mutant because cells were synchronized and sampled at 15-minute intervals. Because the S-phase delay in this mutant is relatively brief (approximately 10–15 min to reach a similar DNA content), this original time-window resolution caused us to miss the subtle shift in replication kinetics. In the current study, we performed synchronization experiments with a much higher temporal resolution, collecting samples every 5 min (Fig. 1c). Using this tighter sampling window, we were able to detect the modest 10–15 min delay in S-phase progression that went unnoticed previously. Together, these findings demonstrate that ectopic Orc4-IHΔ binding leads to S-phase progression delay with an accumulation of cells in late S and G2/M phases.

To investigate if these phenotypes stem from DNA damage and S phase checkpoint activation, we first examined if Orc4-IHΔ cells accumulate γ-H2AX, a hallmark for DNA double-strand breaks (DSBs)^20,21^. However, we did not observe a significant difference in γ-H2AX signal between Wt and Orc4-IHΔ cells in S phase (Supplementary Fig. 1c**, upper panel**), suggesting that the Orc4-IHΔ cells do not accumulate DNA double-strand breaks. We reasoned that the strong cell cycle defect in the absence of DNA damage could be caused by a functional intra-S phase checkpoint response^22^. Therefore, we deleted the main S phase checkpoint effector kinase Rad53 in Wt and Orc4-IHΔ cells. Although γ-H2AX levels did not show clear differences between Wt and Orc4-IHΔ cells in a checkpoint-deficient (*rad53Δ*) background (Supplementary Fig. 1c, lower panel), spot tests indicate that Orc4-IHΔ cells rely on a functional S phase checkpoint, especially in the presence of hydroxyurea (HU)-induced replication stress to sustain viability (Fig. 1d).

We next wanted to investigate the cause for the checkpoint dependency of Orc4-IHΔ cells. Since the majority of ORC-IHΔ binding sites are promoter regions upstream of potential Transcription Start Sites (TSSs) (Supplementary Fig. 1a), we hypothesized that this non-canonical binding of a replication initiator complex could interfere with active transcription and cause transcription-replication conflicts (TRCs), which have been previously shown in yeast and human cells to cause DNA damage and checkpoint activation^23,24^. To investigate if there are increased levels of TRCs in Orc4-IHΔ cells, we performed Proximity Ligation Assay (PLA) using antibodies against actively elongating RNAPII (RNAPII Ser2P) and PCNA as components of the transcription and replication machinery, respectively. As expected, we detected a significant increase in the average number of TRC-PLA foci in EdU-positive Orc4-IHΔ cells when compared to Wt cells (Fig. 1e). Previous studies have shown that TRCs can cause checkpoint activation, particularly in the presence of R-loops^24^, RNA:DNA hybrids formed when nascent transcripts reanneal to their template DNA, displacing the non-template strand as single-stranded DNA (ssDNA)^25^. To test whether the growth and cell cycle defects in Orc4-IHΔ cells are caused by R-loops, we measured R-loop levels across synchronized cell populations isolated in G1, early S, and G2/M phases using a dot blot assay (Fig. 1f). As a positive control, we included a double-mutant strain inactivated for both hybrid-processing RNase H enzymes (*rnh1Δ rnh201Δ*), which exhibits robust accumulation of R-loops in this assay^26^. Notably, Orc4-IHΔ cells showed a marked, cell-cycle-dependent accumulation of R-loops, peaking specifically during G2/M phase. This cell cycle phase-specific elevation of RNA:DNA hybrids directly mirrors the timing of our observed G2/M delay (Fig. 1c), strongly implicating R-loop accumulation as a primary driver of the cell cycle defect in Orc4-IHΔ.To further test whether the growth and cell cycle defects in Orc4-IHΔ cells are caused by R-loops, we overexpressed RNase H1 (Rnh1)^27^ in both Wt and Orc4-IHΔ cells, using an estradiol induction system^28^ that allows tight control of RNase H1 levels depending on estradiol concentration (Fig. 1g and Supplementary Fig. 1d). We found that overexpression of Rnh1 in Orc4-IHΔ cells successfully rescues the growth arrest phenotype (Fig. 1g). Further growth analysis shows that overexpression of Rnh1 in Orc4-IHΔ cells restores a similar growth profile as observed in Wt cells (Fig. 1h). Together, these data show that the promiscuous ORC-IHΔ binding at promoters provokes R-loop mediated TRCs and activates the intra S phase checkpoint, leading to slow S phase progression and a G2/M cell cycle arrest.

### MCM binding is uncoupled from ORC binding sites in Orc4-IHΔ mutant cells

**Fig. 2:**
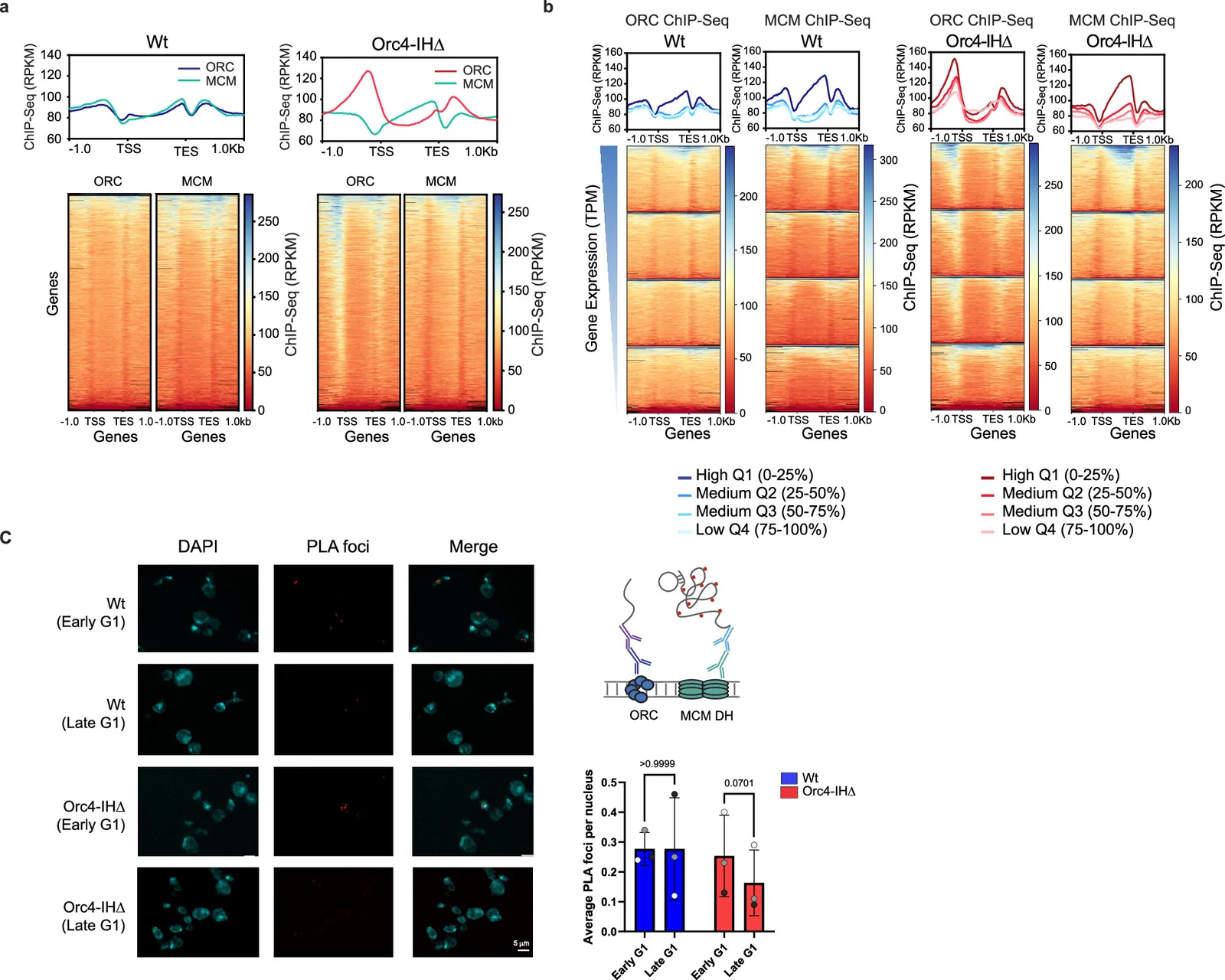
MCM binding is uncoupled from ORC binding sites in Orc4-IHΔ mutant cells. **a** Line plots and heatmaps show the ORC and MCM enrichment patterns in Wt and Orc4-IHΔ at annotated transcripts and flanking sequences. The line plots (top) show the average ORC and MCM ChIP-seq signal (RPKM) at all annotated transcripts and the surrounding regions ( ± 1 kb) in Wt and Orc4-IHΔ. Mutant ORC-IHΔ binding (red) at the 5′ of the transcriptional start site (TSS) is significantly higher than that of wildtype (blue). MCM binding (green) in Orc4-IHΔ is uncoupled from ORC binding, with higher enrichment at the gene body, whereas in Wt, ORC and MCM binding profiles are very similar. Heatmaps (bottom) also suggest the differential enrichment of mutant ORC binding upstream of the TSS and of MCM binding in the gene body regions. **b** Line plots and heatmaps show the ORC and MCM enrichment patterns in Wt and Orc4-IHΔ at annotated transcripts and flanking sequences. The line plots (top) show the average ORC and MCM ChIP-seq signal (RPKM) at transcripts grouped by their gene expression levels, and the surrounding regions ( ± 1 kb) in Wt and Orc4-IHΔ. High Q1 (0–25%) refers to the highly expressed gene quartile. Medium Q2 (25–50%) refers to the moderately high expressed gene quartile. Medium Q3 (50–75%) refers to the moderately low-expressed gene quartile. Low Q4 (75–100%) refers to the lowly expressed gene quartile. The transcripts in the heatmaps are arranged by descending RNA-seq signals. ORC binding intensity is positively correlated with the transcription levels. **c** Left panel: Representative images of Proximity Ligation Assay (PLA) assay with MYC and GFP antibodies targeting ORC and MCM in Wt and Orc4-IHΔ cells. Nuclei were stained with DAPI. Right panel (Low): Quantification of PLA foci number in Wt and Orc4-IHΔ cells at early versus late G1 (*n* = 3). Bars indicate mean values with standard deviations (SD). Paired t-test, two-sided. Right panel (Top): Diagram showing the PLA reaction using two antibodies of different species to label ORC and MCM. Source data associated with this figure are provided in the Source Data file.

To investigate the mechanistic basis for this increase in R-loop mediated TRCs in the Orc4-IHΔ mutant, we first investigated in more detail the binding patterns of ORC and MCM 1 kb upstream and downstream of all genes in Wt and Orc4-IHΔ cells, from previously generated ChIP-seq datasets^13^. In Wt cells, both ORC and MCM binding preferentially occur outside of genic regions, with low signal located upstream of the TSS and downstream of Transcription End Site (TES), respectively. This is consistent with the majority of yeast ARSs as the primary ORC and MCM loading sites located in intergenic regions. Importantly, the pattern of MCM binding closely resembled that of ORC binding (Fig. 2a and Supplementary Fig. 2a), consistent with stable head-to-head MCM double hexamer formation at or very near the ORC loading site. However, in the Orc4-IHΔ mutant, the MCM binding profile showed a clear discrepancy to the ORC-IHΔ binding profile: the majority of the Orc4-IHΔ signal accumulates upstream of the TSS, whereas MCM is enriched towards the 3’ end of the gene bodies upstream of the TES (Fig. 2a and Supplementary Fig. 2a). One plausible explanation is that in the ORC-IHΔ cells, ORC-IHΔ loads predominantly upstream and downstream of TSSs and TESs, respectively, and without the presence of B2 element in the promoter regions, single instead of double MCM hexamers are loaded. As a result, loaded single hexamers at both ends of a gene can slide on the chromatin and formation of a stable MCM double hexamer is only achieved when two single hexamers meet in the correct orientation in the gene body.

Previous studies showed that RNA polymerases can push MCM hexamers in the direction of transcription^18^. Thus, we speculated that the redistribution of MCM hexamers within gene bodies in Orc4-IHΔ cells may be correlated with the transcription direction and relative expression level of the gene. Consistently, we found that the position of MCM hexamers in Orc4-IHΔ cells was skewed towards the 3’ end of genes (Fig. 2a) and the top 25% highly expressed genes (Q1) showed the strongest enrichment of MCMs in gene bodies with a gradual decrease at medium (Q2 and Q3) and lowly transcribed (Q4) genes (Fig. 2b). Interestingly, we also observed robust binding of Wt ORC and MCM in the gene bodies of Q1 highly transcribed genes (Fig. 2b), suggesting that the relocation of MCM hexamers can also occur in the most highly transcribed genes of Wt cells (Fig. 2b).

To directly test whether MCM complexes are physically displaced from their initial ORC deposition sites prior to S phase entry, we tracked the spatial proximity of epitope-tagged ORC (Orc6-9xMyc) and MCM (Mcm7-GFP) using a Proximity Ligation Assay (PLA) in synchronized early and late G1 cell populations. Synchrony was achieved by arresting cells in G2 phase using nocodazole and release in fresh medium containing alpha factor, sampled at 15 minutes interval, early and late G1 timepoints were determined by FACS profile (Supplementary Fig. 2b). In Wt cells, ORC-MCM average number of PLA foci per cell remained stable throughout G1. In contrast, Orc4-IHΔ cells tend to show a decreased number of ORC-MCM PLA foci per cell as they transition from early to late G1 phase (Fig. 2c and Supplementary Fig. 2b). This physical uncoupling indicates that while Orc4-IHΔ cells initially load MCM complexes at non-canonical promoter sites in early G1, active transcription subsequently displaces these loaded single hexamers away from ORC and into downstream gene bodies before replication begins, providing an independent experimental validation for our transcription-driven sliding model.

### Uncoupled ORC and MCM binding leads to R-loop mediated disruption of the transcription program

**Fig. 3:**
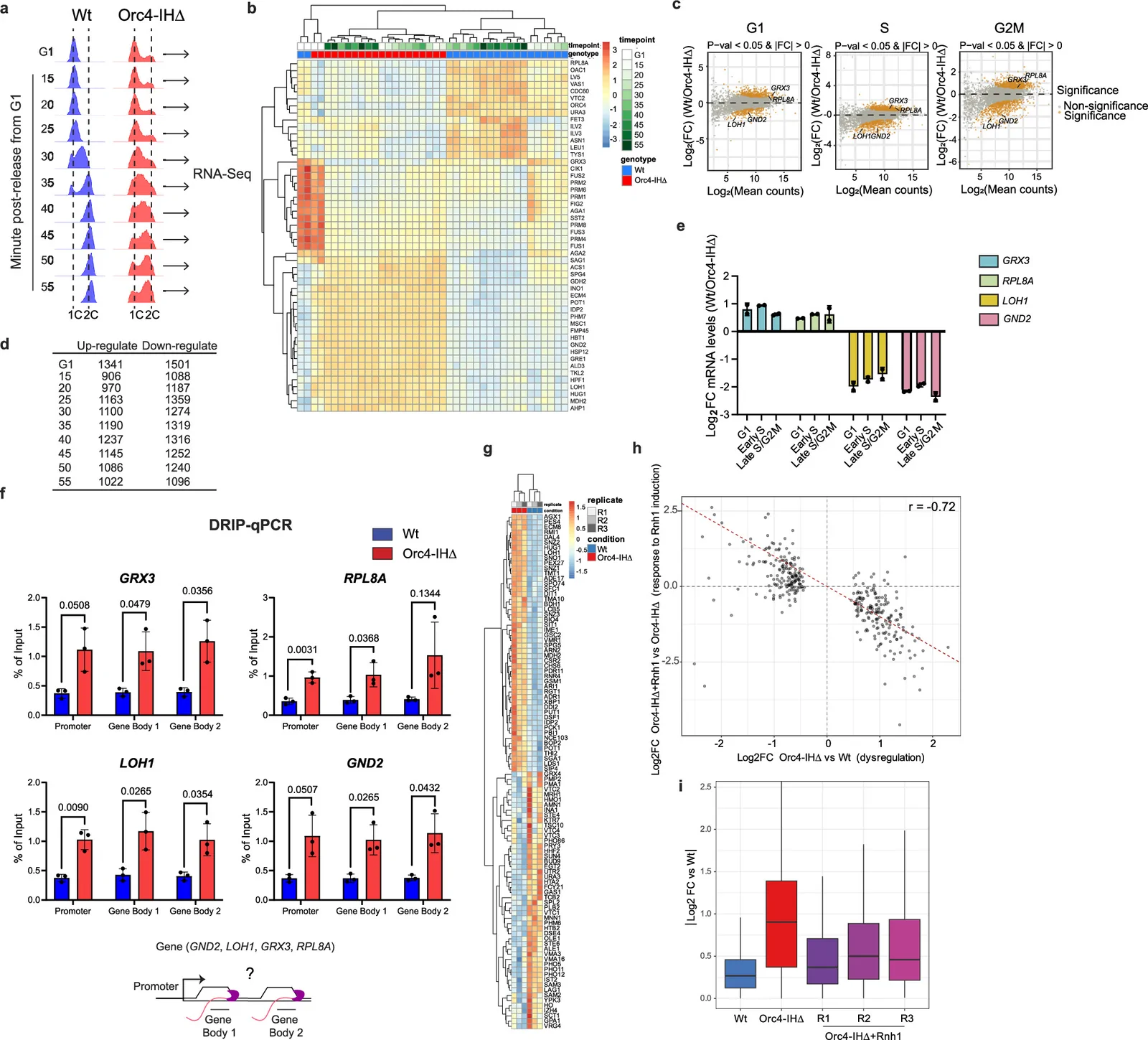
Decoupled ORC and MCM binding leads to R-loop mediated disruption of the transcription program. **a** FACS profiles of samples for RNA-seq libraries preparation. Wt and Orc4-IHΔ cells were first arrested in G1 using alpha factor and then released into S phase, samples were collected at 5 min intervals for library construction. A total of two biological replicates were collected. **b** Dendrogram heatmap of the top 50 genes that show gene expression changes between Wt and Orc4-IHΔ independent of cell-cycle stages. **c** Minus-Average (MA) plots show differentially expressed genes (significant) between Wt and Orc4-IHΔ at three key stages of the cell cycles (G1, S, G2M). Candidate genes (*GND2*, *GRX3*, *RPL8A*, *LOH1*) that were used for RT-qPCR validation in Fig. 3e were labeled. Significance of differential gene expression were assessed using the Wald test, and *p*-values were adjusted using the Benjamini-Hochberg false discovery rate (FDR) procedure. **d** Table showing the number of Up-regulated or Down-Regulated genes at each of the ten RNA-seq timepoints between the Wt and Orc4-IHΔ strains. **e** RT-qPCR validation of candidate genes mRNA levels at key cell cycle stages between Wt and Orc4-IHΔ using primer pair targeting genic regions. Error bars indicate mean values with standard deviations (SD). Plots shown are representative of *n*  =  2 biological replicates. **f** DRIP-qPCR analysis showing R-loop levels at four candidate genes (*GND2, LOH1, GRX3, RPL8A*) in Wt and Orc4-IHΔ using primer pair targeting promoter and genic regions as illustrated in the cartoon (bottom panel). Error bars indicate mean values with SD. Paired t-test, two-sided. Plots shown are representative of *n*  =  3 biological replicates. Source data are provided as a Source Data file. **g** Dendrogram heatmap of the top 100 genes that show up or down-regulated gene expression between Wt versus Orc4-IHΔ. **h** Rescue scatter plot showing Log2 Fold Change of genes significantly dysregulated in Orc4-IHΔ vs Wt against their Log2FC in Orc4-IHΔ (expressed RNH1) vs Orc4-IHΔ. The dashed diagonal marks exact 1:1 reversal (full rescue back to Wt level); points on it represent complete recovery, points at the origin represent no change from Orc4-IHΔ, and points on the horizontal axis (y = 0) represent no rescue at all. **i** Absolute Log2 fold change (FC) of Wt, Orc4-IHΔ, and three replicates of Orc4-IHΔ overexpressing RNH1 (R1, R2, R3) versus Wt. Plots shown are representative of *n*  =  3 biological replicates. All source data associated with this figure are provided in the Source Data file.

To investigate if the increase in R-loop mediated TRCs in Orc4-IHΔ cells interferes with the transcription program throughout the cell cycle, we performed a time-course RNA-seq in both Wt and Orc4-IHΔ cells. Cells were arrested in G1 phase followed by their synchronized release into S phase until completion of one cell cycle, as monitored by FACS analysis (Fig. 3a). Notably, alpha factor arrested Orc4-IHΔ cells maintained a small population of G2/M cells with 2 N DNA content that were unable to divide even after prolonged arrest for 4 h (Supplementary Fig. 3a), suggesting that this population is permanently arrested in G2/M phase, consistent with the single-cell microscopy results (Fig. 1b). After release into S phase, samples were collected at 5-minute intervals and processed for bulk RNA-sequencing (Fig. 3a). Principal Component Analysis (PCA) showed a clear separation of the gene expression profiles between Wt and Orc4-IHΔ genotypes, with principal component one (PC1) explaining 43.7% of the variance (Supplementary Fig. 3b). This suggests that genome-wide relocation of ORC-IHΔ to promoter regions has a major impact on gene expression independent of the cell cycle state. 10.2% of the variance in gene expression pattern was observed on principal component two (PC2) that separated early from late cell cycle timepoints (Supplementary Fig. 3b). Interestingly, Orc4-IHΔ samples showed much less variance in PC2 compared to Wt samples, indicating that their expression profiles are more similar and less affected by distinct cell cycle distributions. In line with the PCA analysis, we observed several hundred genes that were consistently up- or down-regulated in Orc4-IHΔ cells compared to Wt independent of cell cycle status (Fig. 3b). Overall, we observed ≥ 1000 genes that were either up- or down-regulated in Orc4-IHΔ cells at each timepoint across the cell cycle (Fig. 3d). This was also the case at the timepoints where the large majority of Wt and Orc4-IHΔ cells were in the same cell cycle stage in either G1 phase, S phase (30 min timepoint) or G2/M phase (50 min timepoint) (Fig. 3a, c), excluding the possibility that the altered cell cycle distribution in Orc4-IHΔ cells is the main cause of observed gene expression changes. Interestingly, genes with constitutively expressed promoters show lower differential expression than genes with inducible promoters, consistent with former observation that constitutive genes are less sensitive to genetic perturbations and environmental stimuli^29^ (Supplementary Fig. 3d). Consistently, we observed in the Orc4-IHΔ cells a large overlap of 40–50% and 50–60% of genes that were up- or down-regulated across the G1, S, and G2/M cell cycle stages, respectively (Supplementary Fig. 3e). To further explore which gene sets or molecular pathways are affected in Wt and Orc4-IHΔ cells, we performed gene ontology (GO) term cluster analysis of the differentially expressed genes. In the resulting heatmaps, GO terms are grouped by semantic similarity, with red diagonal blocks reflecting distinct clusters of functionally related biological processes summarized by word clouds (Supplementary Fig. 3f). We found that genes related to ribosome structure, ribosome biogenesis and translation are downregulated in the Orc4-IHΔ mutant, whereas genes related to sporulation and stress response are upregulated in the Orc4-IHΔ mutant (Supplementary Fig. 3f), consistent with a reduction in cellular fitness and metabolic rates, which could be triggered as a secondary response to the observed TRCs and S phase checkpoint activation.

Beyond these secondary stress responses, we reasoned that the primary transcriptional changes could stem directly from physical interference by mislocalized replisome components. Specifically, ORC binding at promoters could interfere with transcription initiation, whereas redistributed MCM hexamers within gene bodies could pose an obstacle for incoming RNAPII, hence affecting the gene expression level. To determine the effect of ORC and MCM binding on overall gene expression, we performed Pearson Correlation (PC) analysis of the dysregulated genes from our time-course RNA-seq dataset and the binding intensity of ORC and MCM at the promoters and gene bodies, respectively (Supplementary Fig. 3c). In G1 phase, we observed no correlation between ORC/MCM binding and gene expression changes in Orc4-IHΔ versus Wt cells (Pearson correlation values of 0.01 and 0.05). Despite the fact that many genes related to sporulation and stress response were upregulated in Orc4-IHΔ cells (Supplementary Fig. 3f), we did observe in S phase and more prominently in G2/M phase, a weak but visible negative correlation of ORC and MCM binding and gene expression level, indicating that the redistributed ORC/MCM complexes in Orc4-IHΔ cells have a stronger tendency to repress gene expression in late S phase (Supplementary Fig. 3c). Together, these data support a model that the non-canonical binding of ORC-IHΔ in Orc4-IHΔ cells at promoters leads to MCM loading and redistribution to gene bodies that can lead to repression of transcription, particularly in highly transcribed genes.

An additional, non-mutually exclusive driver of these widespread gene expression changes could be the global accumulation of R-loops in Orc4-IHΔ cells. To test this hypothesis, we first performed targeted RT-qPCR on four representative candidate genes that showed consistent upregulation (*GRX3, RPL8A*) or downregulation (*LOH1, GND2*) across multiple cell cycle stages in Orc4-IHΔ compared to Wt cells. This analysis confirmed that these expression changes were maintained across G1, early S, and late S/G2-M phases (Fig. 3e). Furthermore, S9.6 DRIP-qPCR across these same loci revealed a significant accumulation of R-loops in Orc4-IHΔ cells at both promoter and gene body regions of these genes (Fig. 3f), suggesting that ectopic ORC binding and aberrant MCM loading directly promote R-loop stabilization, in line with our global analysis by dot blot (Fig. 1f). Remarkably, this R-loop accumulation occurred at both up- and downregulated genes. This suggests that the induction of R-loops in Orc4-IHΔ cells inherently destabilizes the transcriptional program by disrupting elongation homeostasis, with R-loops impairing transcriptional elongation at some genes (as seen for *GRX3* and *RPL8A*), while favoring transcription initiation at other loci (*LOH1, GND2*), potentially by altering local chromatin accessibility or polymerase dynamics.

To evaluate the implication of R-loops as the source of the transcriptional rewiring in Orc4-IHΔ cells, we performed RNA-seq on Wt and Orc4-IHΔ cells with and without Rnh1 overexpression across three biological replicates. Analysis of log2fold-changes for genes dysregulated in Orc4-IHΔ cells versus Wt cells revealed a strong negative correlation (*r* = − 0.722) upon Rnh1 induction, confirming a systemic reversal of the mutant expression signature back toward wildtype levels (Fig. 3g, h). Interestingly, individual replicates exhibited varying degrees of Rnh1 induction, with Replicate 1 (R1) showing the highest, Replicate 2 (R2) intermediate, and Replicate 3 (R3) the lowest Rnh1 overexpression levels (Supplementary Fig. 3h). Critically, the magnitude of the transcriptional rescue scaled directly with Rnh1 expression levels, as quantifying the transcriptional recovery back to Wt levels revealed that Replicate 1 achieved the highest rescue reverting the majority of differentially expressed genes back to Wt levels, whereas the other two replicates showed a more modest rescue effect (Fig. 3i) Consistently, Replicate 1 clustered most closely with wildtype samples in Principal Component Analysis (Supplementary Fig. 3g). Together, these results demonstrate that the transcriptional defects in Orc4-IHΔ cells are largely R-loop-dependent, directly linking ectopic licensing and TRC formation to global gene expression dysregulation.

### ORC binding at promoters can stimulate both transcription and replication processes

**Fig. 4:**
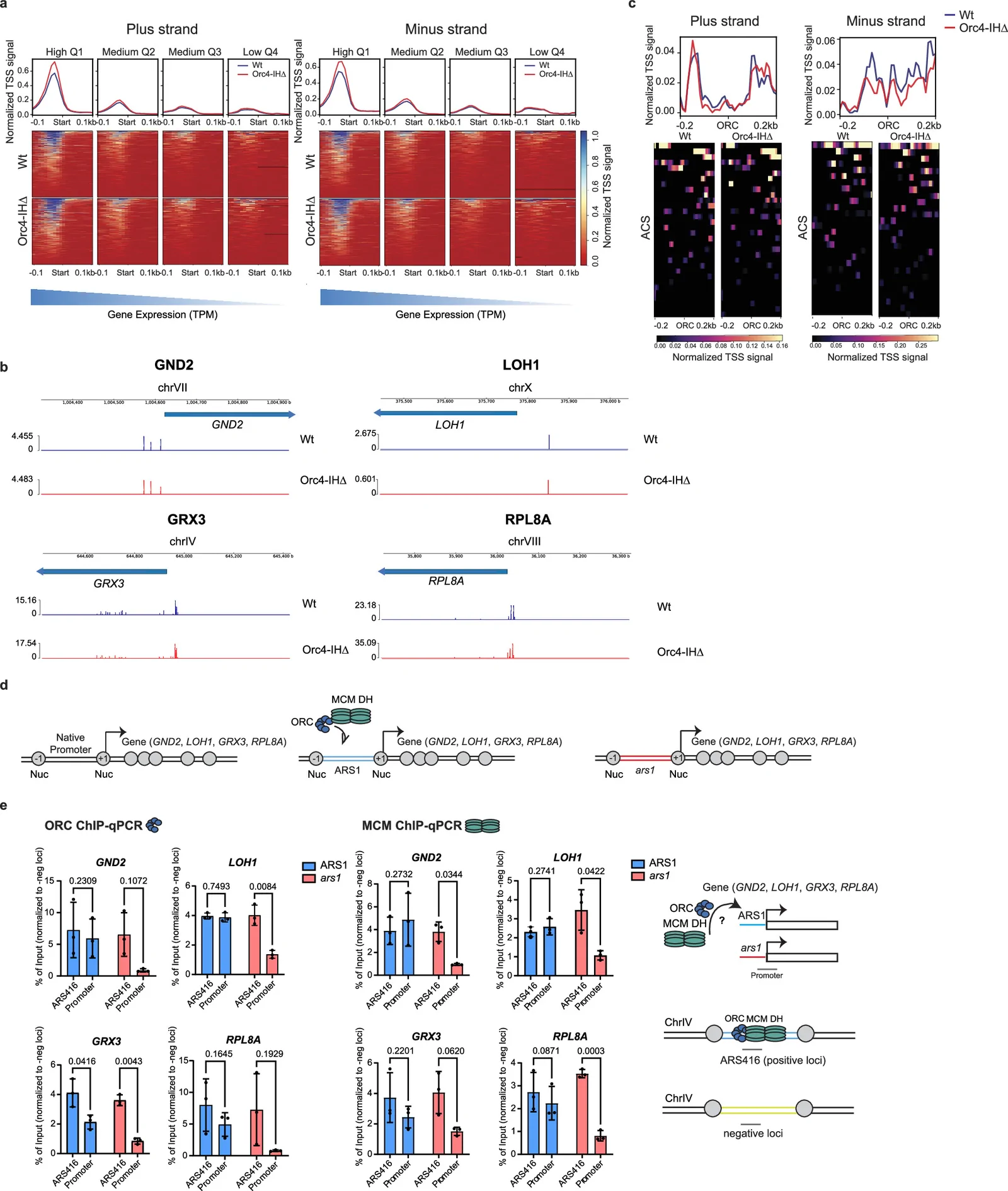
ORC binding at promoters can stimulate both transcription and replication processes. **a** Aggregated signal intensity plots and heatmaps showing TSS signals found in Wt and Orc4-IHΔ surrounding 100 bp upstream and downstream of the start codon of the gene. Genes were classified into four subsets based on transcript levels. High Q1 (0–25%) refers to the highly expressed gene quartile. Medium Q2 (25–50%) refers to the moderately high expressed gene quartile. Medium Q3 (50–75%) refers to the moderately low expressed gene quartile. Low Q4 (75–100%) refers to the lowly expressed gene quartile. Plus and minus strand TSS signals are shown separately. (Top) TSS signal intensity plots of Wt and Orc4-IHΔ surrounding the start codon. (Bottom) Corresponding heatmaps showing TSS signals found in Wt and Orc4-IHΔ at each genic region, each row represents one genic region. **b** Genome browser screenshots of regions on chromosome chrVII, chrX, chrIV, and chrVIII showing transcription start site locations (STRIPE-Seq) in the *GND2, LOH1, GRX3*, and *RPL8A* promoter regions in Wt and Orc4-IHΔ mutant. **c** Aggregated signal intensity plots and heatmaps showing TSS signals found in Wt and Orc4-IHΔ surrounding 200 bp upstream and downstream of the ORC-binding ACS sites. Plus and minus TSS signals are shown separately. (Top) TSS signal intensity plots of Wt and Orc4-IHΔ surrounding the center of the ORC binding site. (Bottom) Corresponding heatmaps showing TSS signals found in Wt and Orc4-IHΔ at each ACS region, each row represents one ACS region. **d** Schematic diagram showing the design of replacement of single endogenous gene promoter (*GND2, LOH1, GRX3, RPL8A*) nucleosome-free regions between plus-one and minus-one nucleosome with the wildtype ARS1 and mutant *ars1* sequence. **e** ChIP-qPCR analysis showing ORC and MCM levels at replaced promoter sequences. ChIP-qPCR shows ORC levels (Left panel) and MCM levels (middle panel) in the replaced promoter (ARS1 or *ars1)* sequence of four candidate genes (*GND2, LOH1, GRX3, RPL8A*) using the promoter primer pair illustrated in the cartoon (right panel). ARS416 serves as the positive control for ORC and MCM signal intensity. Error bars indicate mean values with SD. Unpaired t-test, two-sided. Plots shown are representative of *n*  =  3 biological replicates. Source data associated with this figure are provided in the Source Data file.

We next wondered how the promiscuous binding of ORC at promoters in Orc4-IHΔ cells affects transcription initiation frequency and transcription start site (TSS) selection. To this end, we performed STRIPE-Seq in Wt and Orc4-IHΔ cells, a method that captures capped 5’ RNA ends to precisely map active TSS and quantify promoter activity at single-nucleotide resolution^30^. When comparing the location and strength of TSS signals in all promoter regions of Wt and Orc4-IHΔ cells, we observed a consistent increase in TSS signal in Orc4-IHΔ cells on both plus and minus strand genes, particularly in the subset of the top 25% highly expressed genes (Q1) (Fig. 4a), indicating that ORC binding at promoters directly stimulates TSS usage and transcription initiation, particularly at strong promoters that show preferential relocation in Orc4-IHΔ cells (Supplementary Fig. 1a^13^), providing a mechanistic basis for how mutated Orc4 disrupts transcriptional homeostasis and drives broad expression rewiring across the genome. Importantly, this enhanced initiation frequency occurred without altering TSS selection, as peak coordinates were virtually indistinguishable between strains, as visualized in representative genome browser tracks of four candidate gene loci (Fig. 4b). Because ORC has this transcription stimulating property and it is sequestered away from origins to promoters in Orc4-IHΔ cells, we next examined the reciprocal consequences at origin sites. To this end, we analyzed our STRIPE-Seq data in the Wt and Orc4-IHΔ cells to compare TSS locations and usage at or around canonical replication origins. Interestingly, we found that a subset of ~ 13% of the annotated ARSs showed a TSS signal in proximity within 200 bp around the ACS. Interestingly, in this subset, Orc4-IHΔ cells showed in both plus and minus strands a slight reduction in TSS signal around the ACS ORC binding sites (Fig. 4c, top aggregate plots). Altogether, this reciprocal shift in TSS usage supports a model where origins may exhibit cryptic or weak promoter activity that is functionally stimulated by ORC binding, providing a novel mechanistic link between origin licensing complexes and local transcriptional control.

In parallel, to investigate whether origin may exhibit promoter activity and the effect of ORC binding at promoters on expression of associated genes, we first constructed yeast strains where we replaced the nucleosome-depleted promoter region of four candidate genes (*GND2, LOH1, GRX3, RPL8A*) between the +1 and − 1 nucleosome^31^ with wildtype ARS1 sequence, which is well known to recruit ORC and subsequent MCM loading. Previous work has shown that the mutant *ars1* sequence, which contains point mutations at A and B2 elements, is unable to recruit ORC and MCM^32^. Therefore, we also constructed yeast strains with corresponding gene promoters replaced with mutant *ars1* sequence to assess gene activity in the absence and presence of ORC binding (Fig. 4d). We first performed ChIP (Chromatin ImmunoPrecipitation)-qPCR experiments to determine the binding levels of epitope-tagged ORC (Orc6-9xMyc) and MCM (Mcm7-6xHA) complexes (Supplementary Fig. 4a) at promoter regions replaced with ARS1 or mutant *ars1* sequences. The native ARS1 (ARS416) origin on chromosome IV served as a positive control for ORC and MCM signals (Fig. 4e, right panel). For all four candidate genes, we found that the promoter-replacing ARS1 sequence has a similar ORC and MCM binding signal as the native ARS1 (ARS416) origin. In agreement, promoters replaced with mutant *ars1* sequences showed a clear decrease in ORC and MCM binding signals compared to the native ARS1 (ARS416) origin in all four candidate genes. In fact, the signals were comparable to those at a negative control locus, which is a region ~ 3 kb upstream of ARS1 (ARS416) previously shown to be devoid of ORC and MCM^13^ (Fig. 4e). These results suggest that the replaced ARS1 sequence retains the origin licensing function, whereas the replaced mutant *ars1* sequence is unable to recruit ORC and MCM, consistent with previous findings^32^.

To further elucidate whether the replaced ARS1 sequence can serve as a functional replication origin, we performed DNA copy number assay in strains with gene promoters replaced with either ARS1 or mutant *ars1* sequences. In this assay, cells were arrested in G1-phase and synchronously released to S phase to monitor Replication Timing (RT) by qPCR^33^. We expected the DNA copy number ratio of the early efficient origin to increase from 0 to 24 min after G1-release, when compared with a late replicating region on ChrIV. After 24 min, we expect replication will start initiating at the late replicating region, hence the copy number ratios should decrease until the completion of S phase (Supplementary Fig. 4b). Our results showed that replacing the ARS1 sequence at the promoters of four gene candidates maintains its origin function and replicates early in S phase, with similar copy number ratios as the early-replicating origin ARS305. On the contrary, the replaced mutant *ars1* sequence fails to initiate replication in early S phase, indicated by a significant decrease in copy number ratios compared with ARS305. One exceptional case was the gene candidate *LOH1*, where we observed no difference between promoter replacement with ARS1 or the mutant *ars1* sequence. This can be explained by the fact that the early-efficient replication origin ARS1012 is located just downstream of the TES of the gene, as indicated by the early replication profile of *LOH1* (Supplementary Fig. 4b), therefore allowing early replication of this domain independent of our promoter substitutions.

Having confirmed site-specific loading of functional pre-RC complexes at the four candidate gene promoters, we next asked whether the MCMs located close to these genic regions could be relocated by transcribing RNA polymerases towards the gene body, as previously shown^18^. To this end, we conducted ChIP-qPCR to monitor MCM signals at the gene bodies of the respective candidate genes. Interestingly, we found MCM signals at the genic regions just downstream of promoter regions in two (*GND2, LOH1*) out of four candidate genes, with *LOH1* showing a more prominent effect (Supplementary Fig. 4c). Our results were concordant with the published in vitro findings that RNA polymerases can push multiple MCM helicases over long distance on chromatin, with nucleosomes being ejected or displaced^18^. Yet this seems not to be a general, robust phenomenon across all investigated genes, suggesting specific locus- and chromatin-context may contribute to this MCM relocation.

### Replication origins exhibit intrinsic promoter activity driven by their ability to form a nucleosome-depleted region

**Fig. 5:**
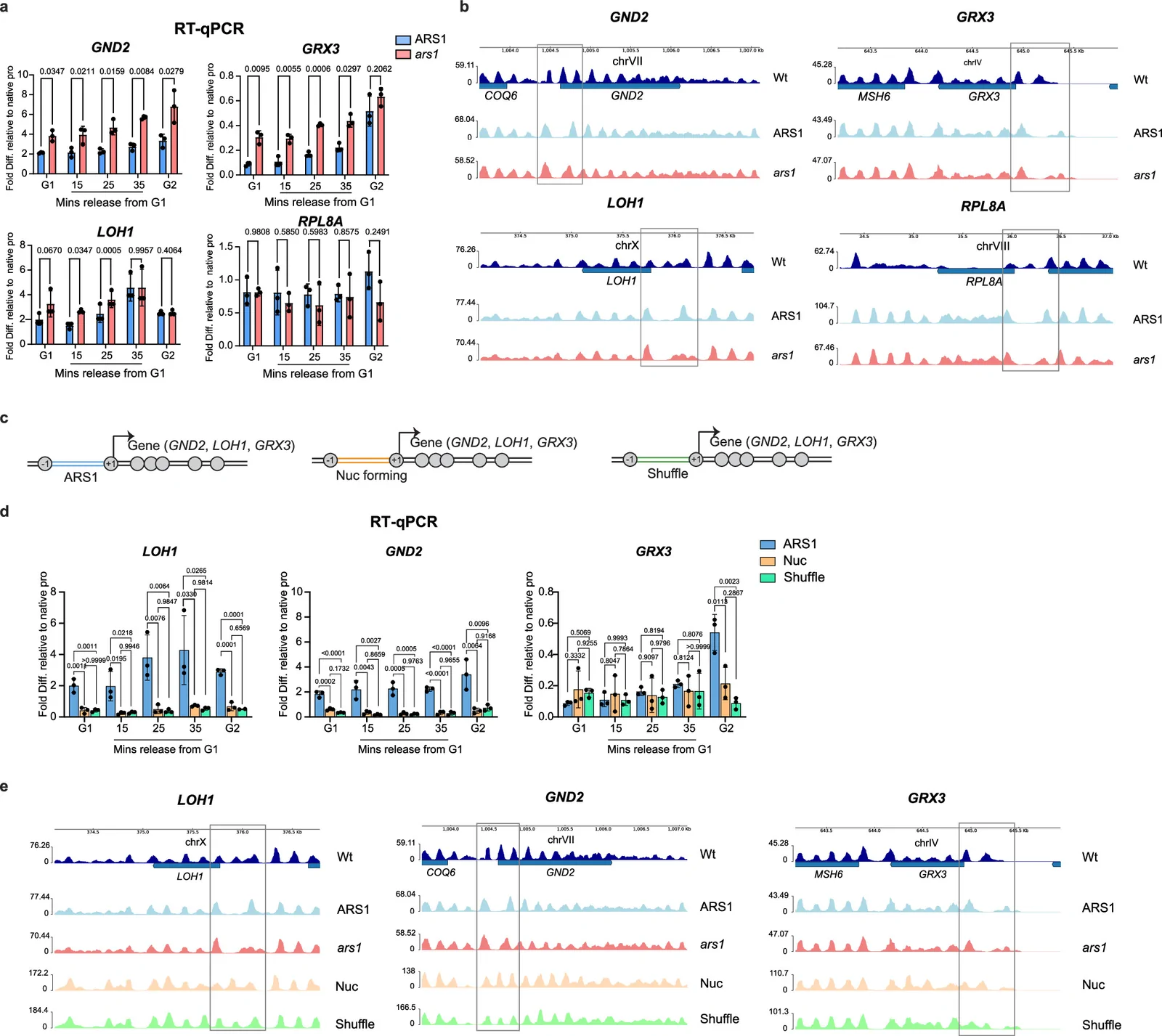
Replication origin sequence shows cryptic promoter function due to its nucleosome-exclusion feature. **a** RT-qPCR analysis of candidate gene expression (*GND2, LOH1, GRX3, RPL8A*) across one cell cycle (G1; 15, 25, 35 min release from G1; G2) between Wt strain and strains with candidate gene promoter replaced either with ARS1 or *ars1*, using primer pairs targeting the genic regions. Error bars indicate mean values with standard deviations (SD) (*n* = 3). Unpaired t-test, two-sided. **b** Genome browser screenshots of regions on chromosome VIII, IV, X, and VII showing nucleosome positioning in candidate genes’ genic and +/− 1 kbp of genic region (*GND2, GRX3, LOH1, RPL8A*) in Wt, and strains with the corresponding candidate gene promoter replaced with ARS1 or mutant *ars1* sequence. Boxes indicate the promoter regions. **c** Schematic diagram showing the design of replacement of single endogenous gene promoter (*GND2, LOH1, GRX3*) nucleosome-free regions between the plus-one and minus-one nucleosome with wildtype ARS1, nucleosome-forming sequence (Nuc-forming), and random shuffled ARS1 sequence (shuffle). **d** RT-qPCR analysis of candidate gene expression (*GND2, LOH1, GRX3*) across one cell cycle (G1; 15, 25, 35 min release from G1; G2) between Wt strain and strains with candidate gene promoter replaced either with ARS1, nucleosome-forming, or random shuffled ARS1 sequence, using primer pairs targeting the genic regions. Error bars indicate mean values with standard deviations (SD) (*n* = 3). Ordinary one-way ANOVA with multiple comparisons. **e** Genome browser screenshots of regions on chromosome X, VII, and IV showing nucleosome positioning in candidate genes’ genic and +/− 1 kbp of the genic region (*LOH1, GND2, GRX3*) in Wt and strains with the corresponding candidate gene promoter replaced with ARS1, mutant *ars1*, nucleosome-forming, and random shuffled ARS1 sequence. Boxes indicate the promoter regions. All source data associated with this figure are provided in the Source Data file.

Having confirmed that the ARS1 sequences inserted in place of candidate gene promoters can serve as functional replication origins, we next assessed if gene expression is retained and how ORC binding at the candidate gene promoters affected their gene expression profile across different cell cycle phases. To this end, we arrested cells in G1 phase and released them into S phase and collected samples at 15, 25, and 35 min post-release from G1. In addition, we arrested cells at G2 phase using nocodazole and then measured steady state transcription levels of the four candidate genes by RT-qPCR. Strikingly, when compared to the endogenous expression levels of the candidate genes controlled by their original native promoter sequence, we discovered that transcriptional output was not eliminated and still detectable in all cases (Fig. 5a). In fact, *GND2* and *LOH1* genes with ARS1/*ars1* promoter replacement showed, across all cell cycle stages a ~ two- to six-fold increased mRNA level compared to the expression levels from their native promoters (Fig. 5a, *GND2, LOH1*). Interestingly, the highly expressed ribosomal protein gene *RPL8A* also showed robust gene expression at a comparable level in the ARS1*/ars1*-substituted promoter strains compared to the wildtype strain (Fig. 5a, *RPL8A*), whereas *GRX3* retained between 10 and 60% of its original mRNA levels depending on the replaced promoter sequences and cell cycle stages (Fig. 5a, *GRX3*). Together, these data suggest that replacing the NDR of promoters with ARS1/*ars1* sequences retains gene expression activity, implying an intrinsic promoter activity of this replication origin sequence.

Interestingly, transcriptional output of *GND2, GRX3, and LOH1*, was significantly affected by the active recruitment of ORC at their gene promoters, especially in the case of *GND2* and *GRX3*. For these three non-constitutively expressed genes, the strains with the replaced ARS1 sequence capable of recruiting ORC at the promoter showed reduced gene expression levels throughout the cell cycle compared to the corresponding *ars1* mutant strains (Fig. 5a, *GND2, GRX3, LOH1*). In contrast, there was no significant difference in mRNA levels between ARS1 and *ars1* mutant strains in the constitutively and highly expressed RPL8A gene (Fig. 5a*RPL8A*). We wanted to further study the molecular mechanism why *GND2* and *GRX3* transcription are strongly affected by the binding of pre-RC components at promoters. We reasoned that the physical presence of ORC/MCM complexes may interfere with the recruitment of initiation-competent RNAPII complexes. To test this, we performed ChIP-qPCR using antibodies against total RNAPII in the strains with *the GND2* or *GRX3* promoter replaced with the ARS1 or *ars1* sequence. Interestingly, we observed similar total RNAPII occupancy levels on either promoters or downstream gene body regions regardless of whether ORC/MCM are binding at the promoters or not (Supplementary Fig. 5a), suggesting that the repressive effect on total mRNA levels is not regulated at the step of RNAPII loading to the gene.

To further investigate the mechanism for the intrinsic promoter activity of the origin sequence, we reasoned that the capacity of ARS1 to sustain transcription depends on its ability to establish an NDR required for pre-initiation complex (PIC) assembly^34^. To test this hypothesis, we performed MNase-seq to map the local chromatin architecture across our promoter-replacement constructs. Indeed, both wildtype ARS1 and mutant *ars1* inherently maintained a robust, well-defined NDR, providing a structural explanation for the persistence of transcriptional output (Fig. 5b).

To rule out the possibility that this transcriptional activity is merely a non-specific artifact of introducing AT-rich DNA, we replaced the native promoter at candidate gene loci with a randomly shuffled sequence (shuffle) matching the exact base composition of ARS1 (Fig. 5c). Strikingly, this shuffled control completely abolished transcription (Fig. 5d). Similarly, replacing ARS1 with a synthetic sequence engineered to strongly position nucleosomes (nuc forming)^35^ also fully eliminated promoter activity (Fig. 5d). MNase-seq profiling revealed that both the shuffled sequence and the nucleosome-positioning construct induced stable nucleosome occlusion over the promoter region, whereas ARS1 preserved the open NDR (Fig. 5e). Together, these results demonstrate the intrinsic capacity of ARS1 to establish and maintain an NDR is the fundamental feature driving its intrinsic promoter function.

### Transcription start site selection at origin-substituted loci is guided by + 1 nucleosome positioning

**Fig. 6:**
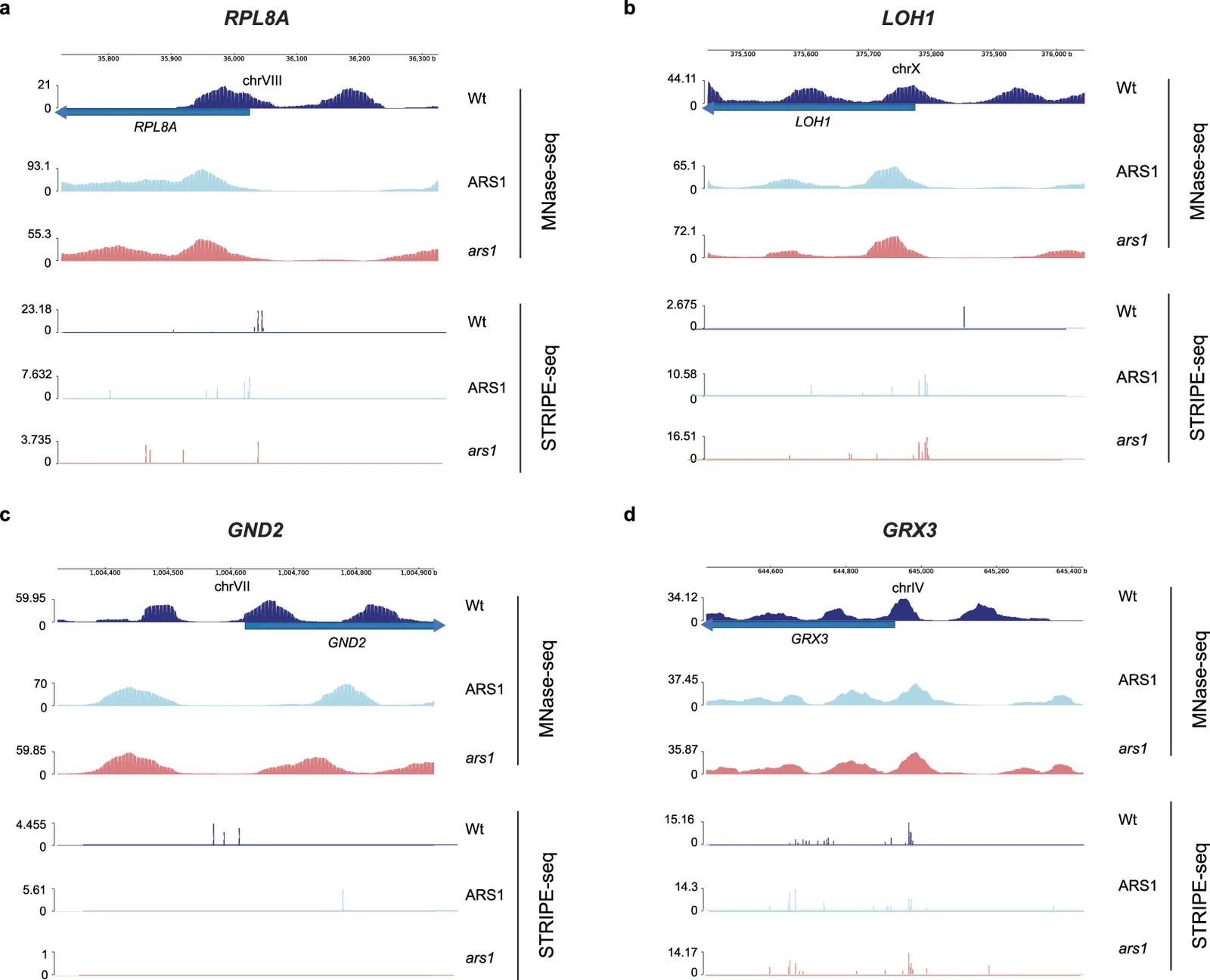
Transcription start site selection is guided by the plus one nucleosome. **a** Genome browser screenshots of regions on chromosome chrVIII showing nucleosome positioning (MNase-Seq) and transcription start site locations (STRIPE-Seq) in the *RPL8A* promoter region in Wt, and strains with the corresponding candidate gene promoter replaced with ARS1 or mutant *ars1* sequence. **b** Genome browser screenshots of regions on chromosome chrX showing nucleosome positioning (MNase-Seq) and transcription start site locations (STRIPE-Seq) in the *LOH1* promoter region in Wt, and strains with the corresponding candidate gene promoter replaced with ARS1 or mutant *ars1* sequence. **c** Genome browser screenshots of regions on chromosome chrVII showing nucleosome positioning (MNase-Seq) and transcription start site locations (STRIPE-Seq) in the *GND2* promoter region in Wt and strains with the corresponding candidate gene promoter replaced with ARS1 or mutant *ars1* sequence. **d** Genome browser screenshots of regions on chromosome chrIV showing nucleosome positioning (MNase-Seq) and transcription start site locations (STRIPE-Seq) in the *GRX3* promoter region in Wt, and strains with the corresponding candidate gene promoter replaced with ARS1 or mutant *ars1* sequence.

To determine whether origin substitution alters transcript structure and stability, we investigated how changing the promoter environment impacts transcription start site (TSS) selection and local chromatin architecture. To this end, we performed STRIPE-seq and overlaid it with our MNase-seq datasets to simultaneously track TSS selection and +1 nucleosome positioning across our candidate loci (Fig. 6a–d). At the *RPL8A* locus, the native promoter exhibits a cluster of closely spaced TSSs located 7 bp upstream of the start codon, positioned immediately adjacent to a well-defined +1 nucleosome (Fig. 6a). Substituting the *RPL8A* promoter with either ARS1 or mutant ars1 altered the local nucleosomal landscape, shifting the +1 nucleosome further downstream into the coding sequence. Strikingly, this repositioning of the +1 nucleosome was accompanied by a concordant downstream shift in TSS usage into the *RPL8A* gene body, resulting in the expression of 5’-truncated mRNA isoforms (Fig. 6a). Despite this spatial shift, total initiation signal intensity remained comparable across all three strains, fully aligning with our RT-qPCR measurements (Fig. 5a). A similar mechanism was observed at the *LOH1* locus, where native expression initiates from a single prominent TSS 78 bp upstream of the start codon (Fig. 6b). In both ARS1 and *ars1* replacement strains, the native TSS was lost. Instead, MNase-seq revealed an altered nucleosomal distribution with a shifted + 1 nucleosome that closely tracked with the appearance of new, highly active cryptic TSSs both near the start codon and within the coding region (Fig. 6b). Overall initiation signal intensity and frequency at *LOH1* were elevated in both origin-substituted strains compared to wildtype, consistent with their increased steady-state mRNA levels (Fig. 5a). At the *GND2* locus, ARS1 insertion similarly reorganized the local chromatin landscape, displacing the + 1 nucleosome downstream into the coding region and driving a robust, corresponding shift in TSS selection into the gene body (Fig. 6c). Interestingly, STRIPE-Seq signal converged from three alternative TSS in Wt cells to one predominant TSS signal under ARS1 substitution (Fig. 6c). Curiously, the *ars1* mutant displayed no detectable TSS signal at the *GND2* promoter region (Fig. 6c) even though RT-qPCR demonstrated robust steady-state transcription (Fig. 5a), which could reflect either lower technical efficiency in capturing short-lived transcripts or the activation of alternative cryptic initiation sites located beyond the scanned promoter window. Finally, at the *GRX3* locus, MNase-seq profiling confirmed that promoter replacement alters the boundary of the nucleosome-depleted region, repositioning the − 1 and + 2 nucleosomes downstream and uncovering cryptic, internal TSSs within the *GRX3* coding sequence (Fig. 6d). Together, these co-localized datasets demonstrate that replacing endogenous promoters with ARS1 or *ars1* sequences maintains robust transcription initiation while altering the precise choice of initiation sites. Fundamentally, the spatial selection of cryptic TSSs is dictated by the repositioning of the + 1 nucleosome, establishing a chromatin-centered model wherein origin-mediated nucleosome-depleted regions drive transcription initiation by setting new local boundaries for pre-initiation complex assembly.

## Discussion

In past decades, studies of replication origins in higher eukaryotes have focused primarily on mapping genomic regions where replication initiates^7,36–38^ rather than identifying the precise locations of ORC-binding sites. Until recently, only a limited number of high-resolution ORC mapping datasets were available in higher eukaryotes^2,17,39^. Our previously published work demonstrated that deleting the 19aa IH of the yeast Orc4 subunit “humanizes” the complex by expanding its DNA-binding repertoire such that it no longer preferentially binds the ACS. Instead, its binding is dictated by the local chromatin landscape with a strong preference for promoter regions (Supplementary Fig. 1a). This aligns closely with observations in higher eukaryotes where most ORC-binding sites are located at or upstream in proximity of promoter regions^1,2,40^. Therefore, our Orc4-IHΔ mutant recapitulates core aspects of higher eukaryotic replication dynamics.

Recent mapping of ORC binding sites in higher eukaryotic genomes indicates its preference at promoters^40^. A central question arising from promoter-centric ORC localization is how cells coordinate replication initiation with transcription to avoid harmful encounters and maintain genome stability. Here we demonstrate that pervasive ORC-IHΔ binding at promoters increases transcription-replication conflicts and R-loop dependent replication stress (Fig. 1). Prominently, dot blot assays revealed a cell-cycle specific accumulation of R-loops peaking during G2/M (Fig. 1f), directly aligning with the pronounced G2/M cell cycle arrest observed in mutant cells. Beyond replication initiation, it is well documented that ORC also plays a non-replicative role in sister chromatid cohesion^41^. Thus, downstream defects in cohesion dynamics may further exacerbate this G2/M arrest phenotype, offering an exciting avenue for future investigation.

Crucially, our time-course RNA-seq revealed that the transcriptional program is disrupted across all cell cycle stages (Fig. 3 and Supplementary Fig. 3). Given that Rnh1 overexpression could partially restore the dysregulated transcription landscape, which correlated directly with Rnh1 induction levels (Fig. 3i and Supplementary Fig. 3h), we further establish that these widespread expression changes are functionally linked to R-loop accumulation. Thus, we postulate that these transcriptional alterations may stem from two non-mutually exclusive processes: systemic stress responses to R-loop mediated TRCs and the physical relocation of MCM complexes into downstream gene bodies (Figs. 2, 3).

**Fig. 7:**
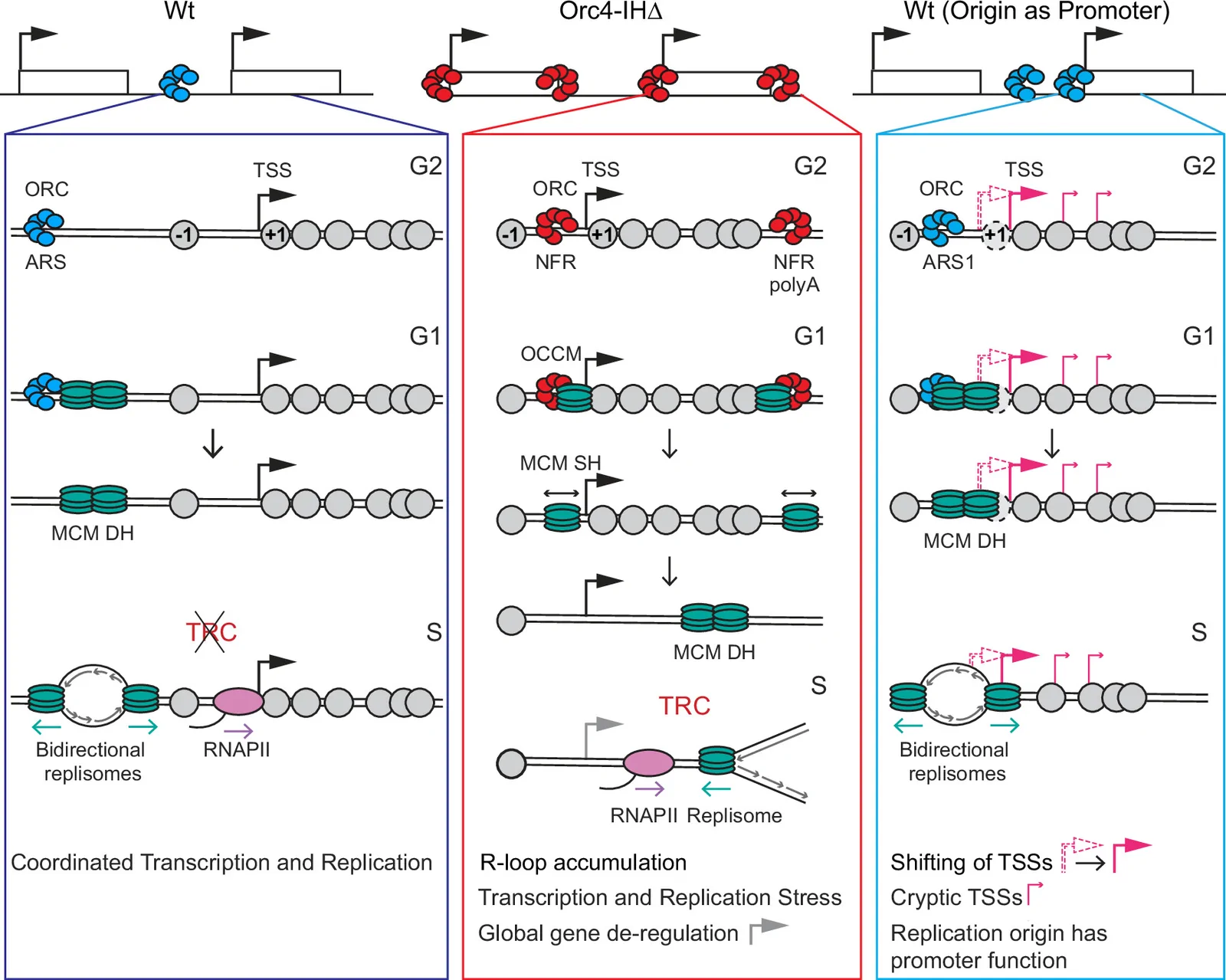
Model showing the interplay of transcription and replication machinery in Wt, Orc4-IHΔ, and strain with promoter substituted with ARS1. In Wt, ORC binds to ARSs locate at intergenic regions, ensuring coordinated transcription and replication, and avoiding conflicts of two machineries. In Orc4-IHΔ, unscheduled binding of mutant ORC at the promoter results in pre-RC loading at the gene bodies, acting either as an obstacle or a traveling replisome for incoming RNAPII, which led to transcription replication conflict, R-loop accumulation, and global gene de-regulation. In strain with the promoter substituted with the ARS1 sequence, change in promoter sequence motifs results in shifting of TSSs and generation of new cryptic TSSs, indicates inherent function of origins as promoters.

More in-depth analysis of the mechanistic reasons that trigger elevated TRCs in the Orc4-IHΔ showed that MCM binding becomes uncoupled from ORC binding sites in Orc4-IHΔ mutant cells, where MCM is loaded preferentially at the gene bodies, possibly due to collision of two single hexamers formed at both ends of genes due to the lack of B2 element at the promoters (Figs. 2a, 7). To directly test this sliding model, we monitored ORC-MCM proximity using Proximity Ligation Assays (PLA) across synchronized G1 populations. We observed a time-dependent loss of ORC-MCM proximity specifically as Orc4-IHΔ mutant cells transitioned from early to late G1 phase (Fig. 2c). This physical displacement provides direct experimental proof that active transcription forces single MCM hexamers away from their initial ORC deposition sites before S phase entry. Furthermore, the accumulation of MCM in Orc4-IHΔ at the gene bodies is positively correlated with the transcriptional strength (Fig. 2b). Interestingly, in the case of wild-type cells, we observed both ORC and MCM enrichment in the gene bodies as well. However, they are exclusively located at highly transcribed genes (Fig. 2b). We speculate that at the highly transcribed gene promoters in the wild-type cells, similar to what happens in the Orc4-IHΔ cells, ORC may bind near the promoter regions, yet the high density of RNAPII may translocate the recruited MCM hexamers to the gene bodies, resulting in subsequent MCM double hexamer formation in the genic regions. This transcription-driven “pushing” of replication machinery has been documented in multiple studies^42,43^.

Apart from looking at the effect of pre-RC formation on gene bodies, we investigated the global transcription initiation events in the Wt and Orc4-IHΔ and uncovered that ORC binding has a stimulatory effect on transcription initiation in highly transcribed genes (Fig. 4a). In addition, we identified transcription initiation events surrounding a small population of ORC binding sites (Fig. 4c). We then parallelly investigated the promoter activity of the origin and the direct effect of ORC and MCM binding at the promoter on transcription activity. In this targeted approach, we replaced the promoter sequences of four candidate genes with the ARS1 or *ars1* origin sequence (Fig. 4d). While licensing non-constitutive promoters suppressed transcription, a highly expressed constitutive promoter remained unaffected (Fig. 5a). This highlights a potential protective mechanism in higher eukaryotes where high-frequency transcription at housekeeping genes efficiently clears or prevents persistent pre-RC interference. In the case of non-constitutive promoters, we observed licensing suppress transcription, however the total RNAPII occupancy remained unchanged in licensing and non-licensing conditions (Supplementary Fig. 5a), therefore ORC/MCM complexes likely impair transcript output by altering RNAPII elongation dynamics or initiation kinetics rather than blocking initial polymerase recruitment.

A particularly striking outcome of these origin-substitution experiments is that origin sequences themselves inherently function as alternative promoters (Fig. 5). By coupling STRIPE-seq with MNase-seq at base-pair resolution, we unraveled the structural basis of this activity: ARS1 inherently establishes and maintains an NDR (Fig. 5b), a feature that is abolished when replaced by scrambled AT-rich sequences or synthetic nucleosome-positioning elements (Fig. 5c, d). Furthermore, our co-localized STRIPE-seq and MNase-seq profiles demonstrate that substituting native promoters with ARS1 alters the local chromatin architecture by repositioning the + 1 nucleosome downstream into the coding region (Fig. 6a–c). Strikingly, the precise choice of transcription start sites (TSSs) tracks directly with this + 1 nucleosome shift, resulting in cryptic, downstream TSSs within the gene body. Thus, the physical boundaries of origin-mediated NDRs dictate TSS selection, demonstrating a fundamental structural overlap and functional plasticity between replication origins and promoters.

In summary, unscheduled and pervasive ORC promoter binding in a compact yeast genome induces genic pre-RC accumulation, global R-loop-mediated TRCs, and widespread transcriptional disruption. Budding yeast avoids these conflicts by restricting ORC and MCM loading to sequence-specific intergenic regions. In contrast, metazoans with complex developmental programs with constantly changing chromatin states could not afford rigid sequence specificity, and therefore rely on accessible promoter NDRs for ORC binding. In spacious mammalian genomes, transcription-driven MCM displacement into broad intergenic spacers may safely mitigate conflicts. However, in gene-dense human genomic domains, unscheduled promoter licensing may trigger the exact TRCs, R-loop stabilization, and expression instability modeled by our humanized yeast strain. These results provide a unifying framework on the choice of origin selection in yeast and humans. Finally, the dual capacity of origin sequences to serve as promoters may ensure that essential open-chromatin loci are prioritized for early replication, providing a broad framework for understanding how replication and transcription machinery co-evolve across eukaryotic genomes.

## Methods

### Live-cell microscopy and image analysis

Live-cell time-lapse phase contrast microscopy was performed using a CellASIC ONIX2 microfluidics system coupled to a Zeiss LSM 800 microscope equipped with a widefield setup and an Axiocam 506 camera. Wt and Orc4-IHΔ cells maintained in YPD medium were briefly sonicated, and 200 μL were loaded into a CellASIC ONIX2 Y04C microfluidic plate. Fresh medium was continuously supplied using a constant pressure of 13.8 kPa. Temperature was controlled to 30 °C using an XLmulti S1 incubation chamber (Pecon) and an objective heater. Cells were imaged every 3 min using a 40x/1.3NA Ph3 oil immersion objective. Images were analyzed using ACDC as described in ref. ^19^ with the YeaZ segmentation model^44^.

### Yeast strains construction

Yeast strains expressing either the wildtype ORC (Wt) or the mutant ORC-IHΔ (Orc4-IHΔ) and corresponding BrdU incorporation strains were generated in a previous study^13^. Yeast strains with the endogenous promoter of *RAD53* replaced with the *GALS* promoter were generated using similar methods as Wt and Orc4-IHΔ strains. Yeast strains with the endogenous *GND2*, *LOH1*, *GRX3*, and *RPL8A* promoters replaced with ARS1,*ars1*, nucleosome-forming sequence, or ARS1-shuffle sequences were generated in the W303-1a background using two-step transformation. Briefly, promoter regions of candidate genes (*GND2*, *LOH1*, *GRX3*, and *RPL8A)* were replaced first with the *URA3* gene using a standard PCR approach, the *URA3* gene was later replaced by origin sequences or nucleosome-forming sequences by plating transformants on medium containing 5-FOA, which selects for recombination events that remove the *URA3* marker. Tandem copies of MYC and HA or GFP tags were fused to the C-terminus of *ORC6* and *MCM7* in the relevant strains for proximity ligation assay and ChIP-qPCR analysis^45^. Yeast strains overexpressing Rnh1 were generated using the estradiol-inducible system^28^. Transformations were done by the standard lithium acetate method. For yeast strains and plasmids used in this study, see Supplementary Data 1 and 3.

### Growth conditions and cell cycle synchronization

For every experiment, Wt, mutant Orc4-IHΔ, Wt-Rad53Δ, and Orc4-IHΔ-Rad53Δ starter cultures were cultured in YP galactose (YPG) medium before shifting to YPD medium to deplete the endogenously expressed Orc4 proteins or Rad53 proteins for at least 18 h at 30 °C until OD_600_ reached 0.4–0.8. Strains were grown at 30 °C in YPD medium for all experiments. Yeast strains with the endogenous *GND2*, *LOH1*, *GRX3*, and *RPL8A* promoters replaced with ARS1,*ars1*, nucleosome-forming, or ARS1-shuffle sequences were maintained in YPD medium and cultured to OD_600_ 0.4–0.8 for all experiments. Overexpression of Rnh1 in Wt and Orc4-IHΔ was achieved by culturing cells in YPD medium containing 20 nM estradiol, for overexpression of Rnh1 in the RNA-seq experiment, cells were cultured in YPD medium containing 20 nM estradiol for ~ 12–14 h at 30 °C. Cells were arrested at G1 phase with alpha factor (GenScript) at a concentration of 10 μg/ml for 2 to 4 h and replenished every 60 min. G2/M arrests were achieved by culturing cells in medium containing 20 μg/ml nocodazole (Sigma) for ~ 2 h. S phase released samples were collected at desired post-release timepoints from G1 arrested cells by replacement with fresh medium.

### Flow cytometry

875 μL volume was taken from each sample to be analyzed, and cells were centrifuged at 16,000 x *g* for 1 min at RT. Supernatant was removed, and cell pellets were washed with 1 ml sterile water. Cells were resuspended in 1 ml 70% ethanol and stored at 4 °C until further use. Before flow cytometry analysis, 1 ml of fixed cells were transferred to a fresh microtube and centrifuged for 16000 x *g* for 1 min at RT. Supernatant was removed, and the pellet dissolved in 300 μl 50 mM sodium citrate containing 0.1 mg/mL RNase A. After 2 h incubation at 50 °C, proteinase K was added to a final concentration of 0.1 mg/ml, followed by another 2 h incubation at 50 °C. 60 μl of digested sample was then mixed with 340 μl 50 mM sodium citrate containing 0.5 μM Sytox Green (S7020, Thermo Fisher Scientific). Prior to FACS analysis, the samples were briefly sonicated (Bioruptor, 5 × 10 sec) to disintegrate cell clumps before proceeding with the flow cytometry with standard gating strategies to identify stained cells (Supplementary Fig.1B).

### Spot test

Sensitivity of Wt and mutant Orc4-IHΔ yeast strains to hydroxyurea (HU), depletion of RAD53, and overexpression of Rnh1 were determined by spotting serial 1/10 dilutions of exponentially growing yeast cultures on YPD plates with or without 50 mM hydroxyurea, YPD only, and YPD plates with or without 20 nM estradiol. All the plates were incubated at 30 °C for a minimum of three days before analysis of spot size.

### Western blot

2.5 OD_600_ units of yeast cells were harvested for each sample to be analyzed by centrifugation for 5 min at 3000 x *g* at room temperature. Cells were washed once with sterile water. Cell pellets were resuspended in 100 μl sterile water, followed by the addition of 100 μl 0.2 M NaOH, the mixture was incubated at RT for 5 mins. Whole cell extracts were pelleted for 5 mins at 3000 xg at RT, and the pellet was resuspended in 50 μl 1x SDS sample buffer. Cell suspension was then boiled for 3 min at 95 °C and was pelleted again before sample loading (1 min, 3000 x *g*). Whole cell extracts were separated by electrophoresis and transferred onto polyvinylidene difluoride membranes (Immobilon®-P PVDF Membrane, Sigma-Aldrich). Membranes were blocked in 5% skimmed milk dissolved in 0.05% Tween/PBS for 1 h at RT. Membranes were then incubated with corresponding primary antibodies overnight at 4 °C followed by washing steps in 0.05% Tween/PBS. Membranes were incubated with appropriate HRP-linked secondary antibodies at RT for 1 h and washed thrice with 0.05% Tween/PBS prior to signal detection. Membranes were developed by chemiluminescence using ECL reagent (Thermo).

### Proximity ligation assay (PLA)

Cells were grown until OD_600_ reached 0.4–0.8, cells were then washed twice with 1XPBS and fixed with 4% PFA for 15 mins at RT. The reactions were quenched with addition of glycine at final concentration of 125 mM. Cells were washed twice with 100 mM KPO4 buffer (pH 6.5) and once with sorbitol wash buffer (1.2 M Sorbitol in 100 mM KPO_4_ buffer) before the spheroplasting step. Cells were resuspended in zymolyase solution (0.5 mg/mL Zymolyase 100 T, 1.2 M Sorbitol, 100 mM KPO_4_ pH 6.5, 20 mM 2-Mercaptoethanol) and spheroplasting was performed at 30 °C for 30 mins. Afterwards cells were centrifuged gently for 1 min at 1000 x *g* and washed once with sorbitol wash buffer (SWB) before resuspended again in SWB. Resuspended cells were seeded on ConA-coated 18 mm coverslips for 2 h or overnight at 4 °C for cell adherence. Next, unattached cells were removed by rinsing once with SWB, followed by permeabilization with 0.2-1% Triton for 3–15 mins at RT before proximity ligation assay^46^. Proximity ligation assay was performed according to the Duolink protocol after the click-it reaction. Samples were incubated with Duolink blocking solution for 60 min at 37 °C, followed by incubation with primary antibodies targeting RNAPII RPB1 (1:400) and budding yeast PCNA (1:400) or MYC-tagged ORC (1:400) and GFP-tagged MCM (1:2000) at 4 °C overnight. On the next day, antibody solution was removed, and cells were washed twice with Duolink wash buffer A (Sigma Aldrich). Subsequently, Duolink PLUS (Sigma Aldrich) and MINUS (Sigma Aldrich) probes diluted with Duolink Antibody Diluent (1:5) were applied to the samples and incubated for 1 h at 37 °C. Then, cells were washed twice with Wash buffer A and the ligation solution (1x Duolink ligation buffer, Ligase at 1:40 dilution) (Sigma-Aldrich) was applied for 30 min at 37 °C. Samples were washed again with wash buffer A and the amplification solution (1x Amplification buffer, Polymerase at 1:80 dilution) (Sigma Aldrich) was added to the samples, followed by incubation in the dark at 37 °C for 100 min. Finally, cells were washed twice with wash buffer B (Sigma Aldrich) and stained with DAPI (0.5ug/ml) in PBS for 30 mins in the dark. Excess DAPI was washed with 1 x PBS, and coverslips were mounted on glass slides with Vectashield Antifade mounting medium and stored at 4 °C until imaging. For EdU-incorporated PLA, Wt-BrdU and Orc4-IHΔ-BrdU strains which allow BrdU incorporation, were cultured in YPD for at least 18 h at 30 °C until it reached mid-log phase at OD600 0.4-0.6. EdU was added to the medium at a final concentration of 10μM for short pulse for 15 min at 30 °C before fixation and spheroplasting and permeabilization steps. Afterwards, cells were washed twice with 1xPBS before proceeding to the EdU click-it reaction as described in ref. ^46^, followed by the duolink protocol.

### RNA-Seq

RNA-seq libraries were prepared from total RNA with rRNA depletion using the QIAseq stranded RNA Library Kit (Qiagen, 180745), according to the manufacturer’s instructions. Total RNA was extracted using the Qiagen RNeasy Micro Kit (Qiagen, 74004) according to the manufacturer’s protocol. In brief, 1 ug total RNA was used as starting material, fragmentation time (95 °C, 10 min) was optimized to generate 150-250 bps fragments, and the libraries were amplified with 12 cycles of PCR. cDNA libraries were sequenced for 76 or 150 cycles in paired-end mode with dual 8 bp indexes, with 20 M reads/sample. Two biological replicates were obtained for the time-course RNA-seq experiment, and three biological replicates were obtained for the Rnh1 overexpression experiment.

### MNase-Seq

Chromatin was digested by MNase (M0247S) to mostly mononucleosomes, as previously described^13^. For sequencing library preparation, DNA samples were quantified with Qubit dsDNA HS Assay Kit (Thermo Scientific), and libraries were prepared using the NEBNext Ultra II DNA Library Kit with Purification Beads (E7103S, E7103L) and NEBNext Multiplex Oligos for Illumina (E6440S), according to the manufacturer’s protocol. Libraries were sequenced for 150 cycles in pair-end mode with 20 M reads/sample.

### Bioinformatic analysis

For RNA-seq, paired-end RNA-seq reads from *Saccharomyces cerevisiae* were quantified using kallisto (v0.48) with 30 bootstrap replicates against the Ensembl release 108 transcriptome and gene annotation. Transcript-level abundances were imported into R (v4.1) with tximport and summarized into a SummarizedExperiment object for downstream analysis to generate PCA plots and a dendrogram. MA plots and GO analysis were generated using separate pipelines. Briefly, read quality was assessed using MultiQC (v1.25.2)^47^. Adapter trimming and quality filtering were performed using fastp (v0.24.0)^48^ with default parameters. Transcript-level abundances were estimated with Salmon (v1.10.3) by pseudo-mapping to the *Saccharomyces cerevisiae* R64-1-1 transcriptome from Ensembl release 114^49^ as reference. Salmon outputs were summarized to the gene level using tximeta (v1.24.0) in R (v4.4.3). Gene-level count matrices were analyzed using DESeq2 (v1.46.0) in R. Normalization was performed using DESeq2’s default median-of-ratios method. Differential expression was assessed using the Wald test, and *p*-values were adjusted using the Benjamini-Hochberg false discovery rate (FDR) procedure. Genes with adjusted p-value < 0.05 and |log_2_ fold-change | > 0 were considered significantly differentially expressed. MA-plots were generated with ggplot2 (v3.5.2) to visualize global patterns of differential expression. Functional enrichment of differentially expressed genes was performed using clusterProfiler (v4.14)^50^ with enrichGO(), using Ensembl gene identifiers and GO Biological Process (BP) annotations from org.Sc.sgd.db (v3.20.0). The default clusterProfiler background (all annotated genes) was used. Enrichment *p*-values were calculated with a cutoff of 0.01 and adjusted using the Benjamini-Hochberg (FDR) procedure; GO terms with adjusted *p* < 0.05 were retained and considered significantly enriched for downstream visualization. Enrichment results were visualized as combined dotplot/gene-concept network (cnetplot) panels per genotype. Redundancy among significant GO terms was reduced by semantic similarity clustering using the simplifyEnrichment package (v2.0.0) (GO_similarity() and simplifyGO()), and GO term overlap between genotypes was visualized jointly using simplifyGOFromMultipleLists(). Cell cycle phases at each timepoints were determined by Cell-Cycle-Scoring in Seurat.

ORC and MCM ChIP-seq normalized signal tracks were generated in the previous study^13^. Reference ACS locations were obtained from GSM424494^51^. All transcript locations were obtained from the UCSC genome browser. A publicly available RNA-seq dataset SRR363968 was used to determine the transcriptional strength of all transcripts in wildtype *S. cerevisiae*. The data was mapped against sacCer2 using STAR aligner (version 2.7.5a). The gene transcript annotation was obtained from Ensembl Genomes. RSEM (version 1.3.3) was used to quantify the gene expression levels in transcripts per million (TPM). Deeptools was used to generate heatmaps and profiles. Scatter plots were generated using ggplot2 with Pearson Correlation. Log2 fold change of ORC and MCM ChIP signal in promoter and gene body of Orc4-IHΔ versus Wt was calculated using bigwigCompare in deeptools.

For STRIPE-seq, sequencing Libraries were processed using a pipeline independently rebuilt from those originally published with the method^30^, applying largely the same tools and parameters. Reads were trimmed with Trimmomatic (v0.39) using adapter clipping (ILLUMINACLIP:TruSeq2-PE.fa:2:30:10), trailing quality trimming (TRAILING:20), minimum length filtering (MINLEN:25), and cropping to 80 bp (CROP:80). Ribosomal RNA contamination was removed with Tagdust (v2.33) using -dust 97 -fe 3. UMIs were extracted with UMI-tools (v1.1.2) from the first 8 nt at the 5′ end of read 1. Adapter/linker removal was performed with cutadapt (v4.0), requiring a minimum read length of 25 bp, perfect matches to the 5′ linker (-g ^TATAGGG -e 0), and secondary adapter removal with --front=CCTACACGACGCTCTTCCGATCT --overlap=10 --error-rate=0.09. Cleaned reads were aligned to the *Saccharomyces cerevisiae* reference genome (Ensembl release 114) using STAR (v2.7) with --genomeSAindexNbases 10. Alignments were filtered for proper pairs and processed with samtools (v1.15), using sort -m 7 G -@ 6 followed by fixmate and markdup -r. Processed BAM files were analyzed in R (v4.1) using TSRchitect and TSRexploreR to define transcription start sites (TSSs) and transcription start regions (TSRs). For each condition and replicate, TSSs were clustered with a maximum distance of 40 bp and a minimum tag count threshold of 5. Count matrices were generated and normalized using the DESeq2 method implemented in TSRexploreR, with additional filtering to retain TSSs supported by ≥ 3 tags in at least one sample. Normalized TSS signal tracks were exported in bedGraph format for visualization downstream analysis.

Paired-end MNase-seq reads were aligned to custom, strain-specific reference sequences comprising the native (“Nuc”) or a randomized (“Shuffle”) variant of an ARS-linked locus (LOH1, GND2, GRX3 or RPL8A) embedded in the corresponding full-length S. cerevisiae chromosome (IV, VII, or X) — using Bowtie2 (v2.5.4) with maximum fragment length set to 250 bp (-X 250, appropriate for mono- and sub-nucleosomal MNase fragments) and --no-discordant --no-mixed to retain only concordant, uniquely mapped read pairs. Alignments were filtered with SAMtools (v1.23) for proper pairing (-f 0×2), multi-mapping reads were excluded (SAM XS:i: tag). Filtered BAMs were converted to paired-end fragment BED files with BEDTools (v2.31.1) and loaded into R as GenomicRanges objects.

Nucleosome dyads were called from fragment coordinates with nucleR (v2.38.0): paired-end fragments were converted to RPM-normalized coverage (processReads/coverage.rpm, computed both untrimmed and trimmed to a central 40-bp window), smoothed by FFT filtering retaining the lowest 2% of frequency components (filterFFT, pcKeepComp = 0.02), and dyad peaks were called as local maxima above the 99th-percentile threshold (peakDetection, threshold = “99%“). For visualization, fragments were independently resized to a fixed 50-bp window centered on the fragment midpoint (dyad proxy) and converted to genome-wide coverage with rtracklayer (v1.66.0), exported as raw and CPM-normalized (scaled to 1 × 10^6 fragments per sample) bigWig tracks. MNase-seq genome browser tracks were output using bigwigAverage and pyGenomeTracks

### Copy number assay

60 ml yeast cultures were grown in YPD medium until they reached their mid-log phase (OD_600_, 0.4-0.8). Cells were then diluted to OD_600_ equal to 0.4 and cells were arrested at G1-phase by addition of alpha factor (10 ug/ml) for two hours, and alpha factor was replenished for every one hour. Cells were released from S phase by replacement of fresh YPD medium. At every 8-minute interval, 4.5 ml sample was added to 500 μl of 1% sodium azide solution (W/V) in 0.2 M EDTA. Samples were washed once with water, and resulting pellets were snap frozen in liquid nitrogen. For DNA extraction, pellets were resuspended into 500 μl RINB (50 mM Tris–HCl pH 8, 0.1 M EDTA, 0.1% (v/v) beta mercaptoethanol) with Zymolyase to a final concentration of 2% (w/v) and were incubated for 1 h at 37 °C and 700 rpm shaking. After addition of 2 μl RNAse A (10 mg/ml), spheroplasts were incubated for 1 h at 37 °C, followed by the addition of 5 μl Proteinase K (10 mg/ml) and 10 μl SDS (20%) at 55 °C for 1 h. DNA was isolated by phenol-chloroform extraction (1:1) followed by overnight ethanol precipitation at − 20 °C. Briefly, one volume (517 μl) of phenol:chloroform:isoamyl alcohol (25:24:1) was added to each sample and vortexed thoroughly for approximately 20 seconds. Samples were then centrifuged at RT for 5 min at 16,000 x *g*. The upper aqueous phase was carefully removed and transferred to a new Eppendorf tube. 5 M NH_4_OAc (0.75 x volume of sample) and 100% ethanol (2.5 × (volume of sample +NH_4_OAc)) were added to the aqueous phase, and samples were placed at –20 °C overnight for DNA precipitation. The next day, samples were centrifuged at 4 °C for 30 min at 16,000 × *g* to pellet the DNA. Supernatant was removed, and pellet was washed with 150 μl of 70% ethanol. Ethanol was removed thoroughly by an extra centrifugation step at 4 °C for 2 min at 16,000 × *g,* and the pellet were dried at room RT for 5–10 min before dissolving in 50 μl of sterile H_2_O by pipetting up and down multiple times.

### Dot blot analysis

Global R-loop detection by dot blot was performed as previously described^26^. Decreasing amounts of genomic DNA extracts, obtained by phenol-extraction and ethanol precipitation, were adsorbed onto nylon membranes (Hybond-N + , GE Healthcare). R-loops were detected using the S9.6 monoclonal antibody (Kerafast; Cat# ENH001; RRID:AB_2687463; 0.3 µg/mL in Tris-Buffered Saline [TBS], 0.5% Tween-20, 5% skimmed milk) and anti-mouse peroxidase-conjugated antibodies (Jackson ImmunoResearch; Cat# 115-035-068; RRID: AB_2338505), using chemiluminescent reagents (Supersignal, Thermo Fisher Scientific) and a ChemiDoc MP Imaging System (Bio-Rad). R-loop levels were normalized to the total amount of DNA in the extracts, as detected using an anti-double-stranded DNA (HYB331-01, Santa Cruz Biotechnology; Cat# sc-58749; RRID: AB_783088; 1:5000 in TBS, 0.5% Tween-20, 5% BSA). Serial dilutions of a reference sample were used as a standard for quantification.

### ChIP-qPCR

ORC, MCM and total RNAPII ChIP were performed as previously described with minor modifications (^13^). In short, about 4 × 10^8^ cells were crosslinked by adding 16% formaldehyde to a final concentration of 1% and incubated at room temperature for 25 min with gentle shaking. Cross-linking was quenched by adding 2.5 M glycine to a final concentration of 0.125 M followed by an additional 10 min incubation at RT. Cells were washed three times with ice-cold 1xTBS. Cells were then resuspended in lysis buffer (1 M Hepes KOH pH 7.5, 5 M NaCl, 0.5 M EDTA pH 8.0, 20% Triton, 5% DOC) and subjected to bead-beating for 10 min at 2000rpm (Thermo mixer, 4 °C) for five times, with 5 min break at every ten minutes. The chromatin fraction was collected by centrifugation and sonicated to yield an average DNA size of 300 bp, using an ultrasonicator (Covaris E220). About 400 μl of clarified sonicated chromatin was subjected to immunoprecipitation with 15 μl of protein A-Dynabeads (Invitrogen) conjugated to anti-MYC antibody (Roche, 9E10) for ORC ChIP, anti-HA antibody (Roche, 3F10) for MCM ChIP, and anti-RNAPII-CTD antibody (Sigma-Aldrich, 8WG16) for total RNAPII ChIP at 4 °C overnight. For the protein A-Dynabeads conjugated to antibodies, 10 μg of antibody was conjugated to 60 μl of protein A-Dynabeads. Immunoprecipitated DNA was then recovered as previously described (^13^). Reverse crosslinked samples were digested with RNase A (ThermoFisher) for 1 h followed by proteinase K (New England Biolabs) for 2 h at 37 °C. DNA was then purified using a MinElute PCR purification column (Qiagen) according to the manufacturer’s instructions. qPCR was performed by mixing DNA with iTaq SYBR Green Supermix according to the manufacturer’s protocol. The enrichment of ORC, MCM, and RNAPII on specific loci was calculated by dividing the percent of input signal of specific loci to negative loci, which is well documented to devoid of signal of either ORC, MCM, or RNAPII. The percent of input signal value implicates how much a genomic locus was recovered in the ChIP sample relative to the amount of that locus present in the chromatin input. The percent of input values were calculated in two steps: in step 1, input was adjusted to 100% by the formula (Ct of input-log2 of dilution factor of input); in step two, percent of input value was calculated by the formula (100*2^(Adjusted input – Ct of IP).

### DRIP-qPCR

DRIP-qPCR was performed using the ChIP-qPCR protocol as described above with minor modifications. Before the immunoprecipitation step, DTT was added to sonicated chromatin to a final concentration of 10 mM. The mixture was incubated at 50 °C for 20 min followed by incubation on ice for 5 minutes before addition of 25 μl of protein A-Dynabeads (Invitrogen) conjugated to anti-S9.6 antibody (SMABE1095) for DRIP ChIP at 4 °C overnight.

### RT-qPCR

About 4 × 10^8^ cells per sample were harvested at desired cell cycle phases. Cells were snap-frozen in liquid nitrogen. The next day, total RNA was extracted using RNeasy Micro Kit (Qiagen), according to the manufacturer’s protocol. Subsequently, cDNA synthesis was performed using the RT-SuperScriptIII kit, following the manufacturer’s protocol (ThermoFisher; Cat#18080051). qPCR was performed as described above.

### STRIPE-seq library preparation

STRIPE-seq libraries were prepared as previously described^30^. Briefly, total RNA was extracted using RNeasy Micro Kit (Qiagen) according to the manufacturer’s protocol. Uncapped RNA was removed by Terminator exonuclease (TEX) treatment followed by template switching reverse transcription (TSRT) reaction and library amplification. Amplified STRIPE-seq libraries were sequenced for 150 cycles in paired-end mode on the Illumina NovaSeq X + 1.5B platform.

### Quantification and statistical analyses

Error bars on Figs. 1b, e, f, 2c, 3f, 4b, 5a, b and Extended Data Figs. 4b, c, 5a indicate the standard deviation of the biological replicates. All statistical details of the experiments can be found in the figure legends. All statistical comparisons were made using GraphPad Prism 10.4.2.

### Reporting summary

Further information on research design is available in the Nature Portfolio Reporting Summary linked to this article.

## Supplementary information

Supplementary Information

Description of Additional Supplementary Files

Supplementary Data 1

Supplementary Data 2

Supplementary Data 3

Reporting Summary

Transparent Peer Review file

## Source data

Source data

## Data Availability

Any additional information required to reanalyze the data reported in this paper is available from the corresponding author upon request. The previously published ORC and MCM ChIP-Seq data are deposited in NCBI’s Gene Expression Omnibus and are accessible through GEO Series accession number GSE149163. The data of time course RNA-Seq, STRIPE-Seq, RNA-Seq in Wt and Orc4-IHD with RNH1 overexpression, MNase-Seq with candidate gene promoter sequence replaced with nucleosome-forming sequence, and MNase-Seq with candidate gene promoter replaced with origin sequence, have been deposited to European Nucleotide Archive (ENA) under accession numbers PRJEB98985 (RNA-Seq), PRJEB100524 (STRIPE-Seq), PRJEB123404 (RNA-Seq with RNH1 overexpression), PRJEB123431 (MNase-Seq for nucleosome-forming sequence), and PRJEB123527 (MNase-Seq for origin sequence), respectively. Source data are provided in this paper.
