# Supplementary material for: Humanized yeast ORC drives R-loop dependent transcription-replication conflicts and global gene dysregulation
Source: Nat Commun. 2026 Nov 13;17:11679. doi: 10.1038/s41467-026-79167-4 (PMC13768333; doi:10.1038/s41467-026-79167-4)
Supplement: Supplementary file 1 [file 41467_2026_79167_MOESM1_ESM.pdf]

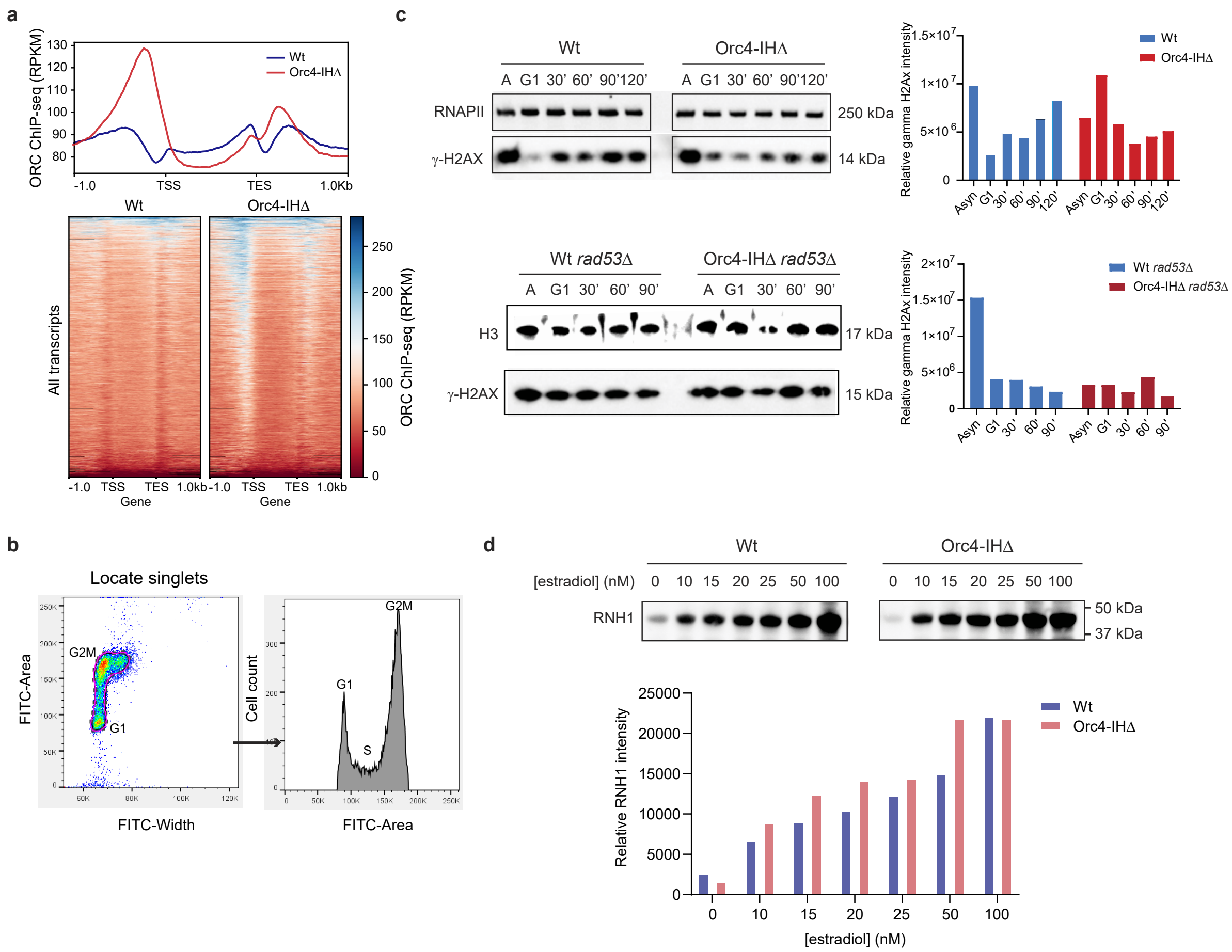

Supplementary Fig. 1.

- **a.** Line plot and heatmaps show the ORC enrichment signals in Wt and Orc4-IHΔ at annotated transcripts and flanking sequences. The line plot (top) shows the average ORC ChIP-seq signal (RPKM) at all annotated transcripts and the surrounding regions (±1 kb) in Wt and Orc4-IHΔ. Mutant ORC-IHΔ binding (red) at the 5′ of the transcriptional start site (TSS) is significantly higher than that of wildtype (blue). Heatmaps (bottom) show differential enrichment of mutant ORC-IHΔ binding upstream of the TSSs.
- **b.** Plots illustrating the gating strategies used in FACS analysis. FITC-Area against FITC-Width gates were used to identify SYBR-Green-stained singlets before the conversion to a histogram.
- **c.** Western blot and corresponding quantification of γ-H2AX of Wt and Orc4-IHΔ in asynchronous phase (A), G1-arrested phase (G1), 30-, 60-, 90-, and 120-minutes

release from arrested G1 phase (30’, 60’, 90’, 120’). RNAPII-phospho-S2 was used as loading control. (Top panel). Western blot and corresponding quantification of γ-H2AX of Wt and Orc4-IHΔ under depletion of Rad53 in asynchronous phase (A), G1-arrested phase (G1), 30-, 60-, and 90-minutes release from arrested G1 phase (30’, 60’, 90’). Histone H3 was used as loading control. (Lower panel). Source data are provided as a Source Data File.

d. Anti-HA Western blot showing three hours induction of HA-tagged RNH1 expression under different concentrations of estradiol in Wt and Orc4-IHΔ containing the estradiol expression cassette (Top panel). Quantification of relative RNH1 western blot signal intensity (Lower panel). Source data are provided as a Source Data File.

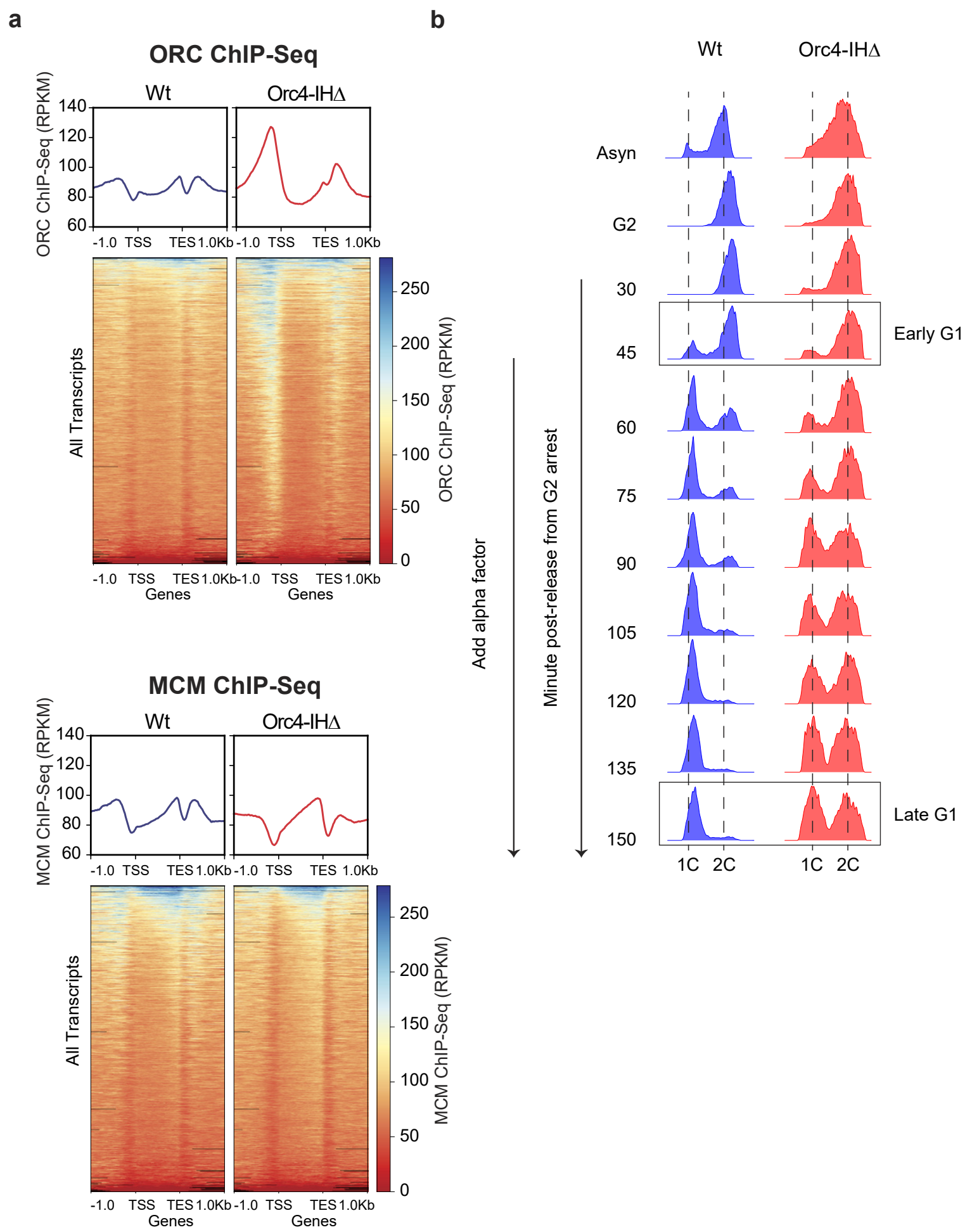

Supplementary Fig. 2.

- **a.** Line plot and heatmaps show the ORC and MCM enrichment signals in Wt and Orc4-IHΔ at annotated transcripts and flanking sequences. The line plots show the average ORC ChIP-seq signal (RPKM) (top) and MCM ChIP-seq signal (RPKM) (bottom) at all annotated transcripts and the surrounding regions (±1 kb) in Wt and Orc4-IHΔ. Heatmaps show corresponding RPKM signal at each transcript.
- **b.** FACS analysis. Wt and Orc4-IHΔ mutant cells were first arrested in G2 using nocodazole and then released into G1-phase (number on the left indicates minutes release from G2), alpha factor was added starting 45 minutes post release from G2 to keep cells in G1 state.

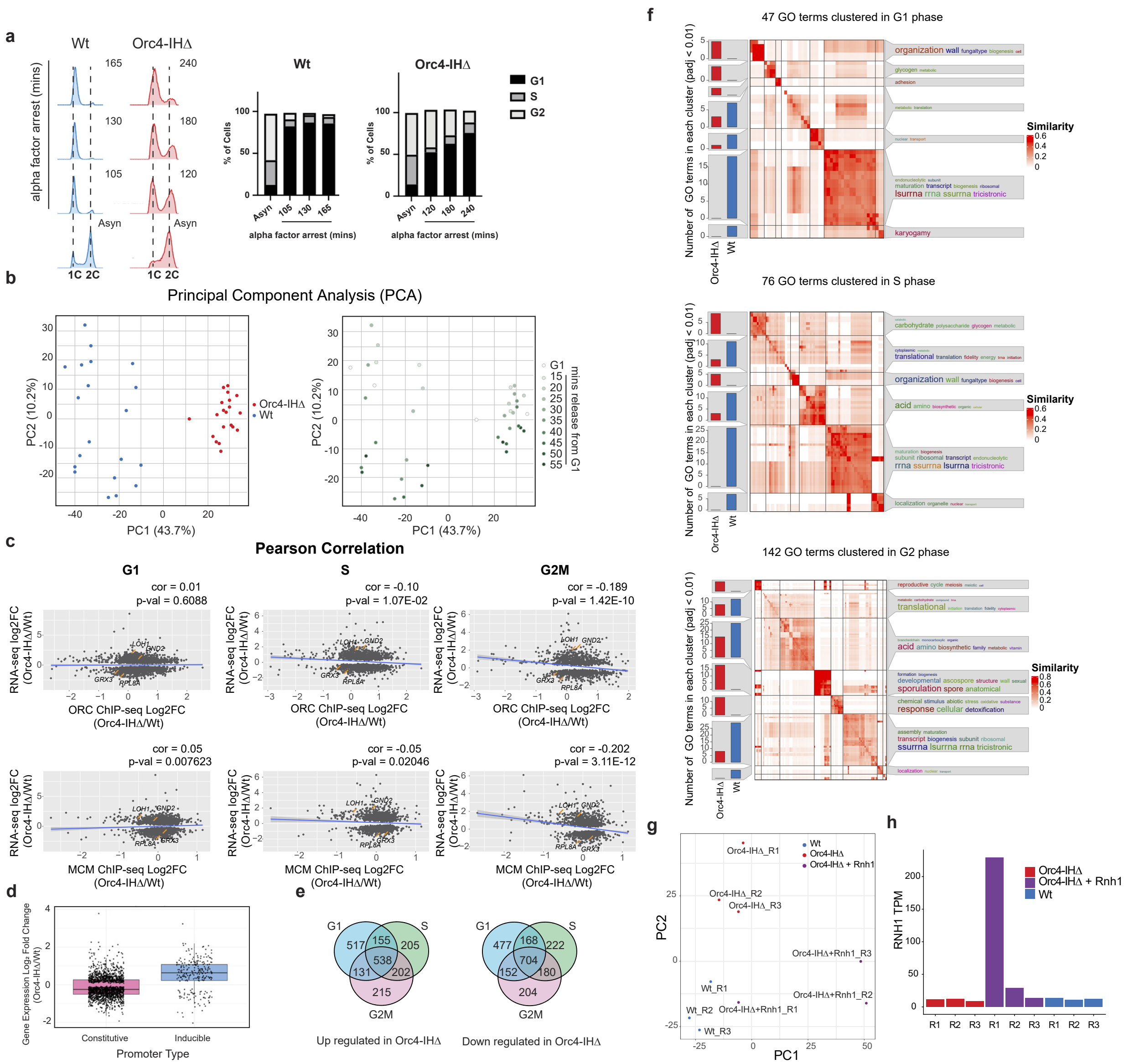

Supplementary Fig. 3.

a. FACS analysis shows the time duration tested to achieve proper alpha factor induced G1 arrest in Wt and Orc4-IHΔ cells. Mutant cells need significantly longer duration for proper arrest. (Left panel) Quantification of percentage of cell in respective cell cycle phases (G1, S, G2) in Wt and Orc4-IHΔ cells at different arrest durations. Source data are provided as a Source Data File.

b. Principal Component Analysis (PCA) plots with Wt and Orc4-IHΔ RNA-seq samples plotted in two dimensions using their projections onto the first two principal components (PC1 and PC2). Colored for samples (Left panel) and

colored for different cell cycle stages (Right panel). Source data are provided as a Source Data File.

- **c.** Scatter plots show correlation between gene expression levels and mutant ORC-IHΔ and MCM binding levels in RPKM at promoters and genic regions at distinct cell cycle phases (G1, S, G2M). (Top) Scatter plots show correlation of gene expression level and mutant ORC-IHΔ binding level within 500bp upstream of the start codon of respective genes. (Bottom) Scatter plots show correlation of gene expression level and mutant MCM binding level within gene body of respective genes. Each dot represents one gene. Pearson correlation coefficient (cor) and p-val shown on the top right corner of each plot. Source Data are provided as Source Data File.
- **d.** Box and whisker plot of the log2 fold-change of gene expression level in Orc4-IHΔ versus Wt separated by constitutively expressed or inducible expressed genes according to promoter classification dataset in[29]. Source Data are provided as Source Data File.
- **e.** Venn diagrams showing the number and overlap of genes that are up- or down-regulated in Orc4-IHΔ across the G1, S, and G/M cell cycle stages. Source Data are provided as Source Data File.
- **f.** Clustering of Gene Ontology (GO) terms in Wt and Orc4-IHΔ at distinct cell cycle phases (G1, S, G2) with word clouds as the annotation. Number of GO terms in each cluster is show as bar plots for Wt (Blue) and Orc4-IHΔ (Red). Prominently up-regulated genes in Wt are ribosomal genes whereas those in Orc4-IHΔ are stress response genes. Source Data are provided as Source Data File.
- **g.** Principal Component Analysis (PCA) plots with Wt, Orc4-IHΔ, and Orc4-IHΔ overexpressing Rnh1 RNA-seq samples plotted in two dimensions using their projections onto the first two principal components (PC1 and PC2). Colored for samples as indicated. Source Data are provided as Source Data File.
- **h.** Quantification of Rnh1 induction in Orc4-IHΔ RNA-seq samples in three biological replicates. Source Data are provided as Source Data File.

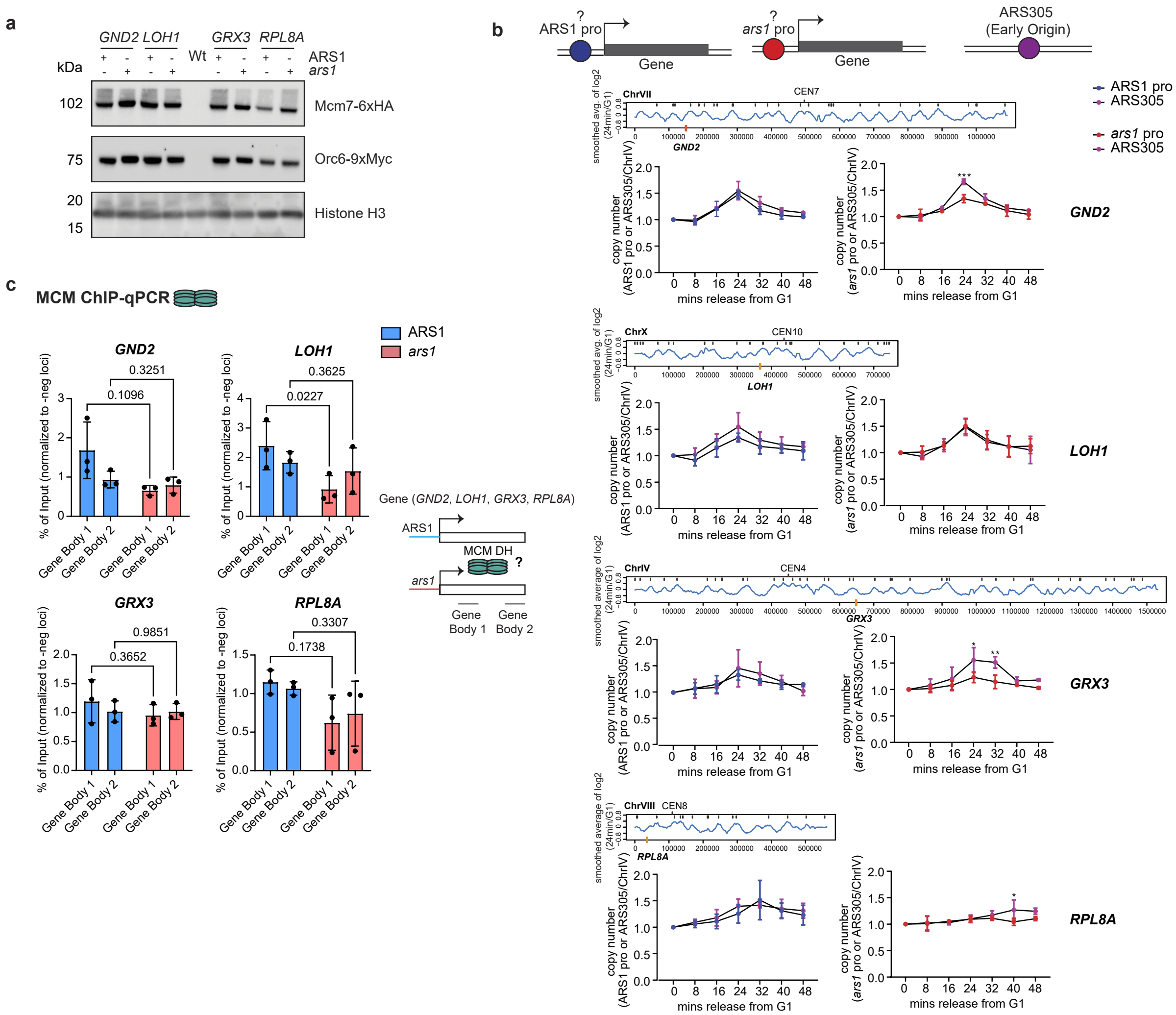

Supplementary Fig. 4.

- **a.** Western blot of HA-tagged MCM and MYC-tagged ORC in Wt and yeast strains with candidate gene (shown on top) promoter replaced by either ARS1 or *ars1* sequences. Wt is the wildtype strain without any gene modifications. Histone H3 was used as loading control. Source Data are provided as a Source Data File.
- **b.** Replication timing analysis of replaced ARS1 (ARS1 pro) and *ars1* (*ars1* pro) sequences at candidate gene (*GND2, LOH1, GRX3, RPL8A*) promoters. The DNA copy number ratios of replaced sequences were compared to early replicating region ARS305 in G1 phase released samples as the timepoints indicated on the x-axis (ratios were calculated as the ratios to the late replicating region of ChrIV). Error bars indicate mean values with standard deviations (SD) (n=3). Two-way

ANOVA with multiple comparisons test. Replication profiles of chromosomes at 24 minutes release from G1 into S phase were shown on top of the line-plots for each candidate genes, respectively. Black bars indicate the ARS regions. Source Data are provided as a Source Data File.

c. ChIP-qPCR analysis showing MCM levels at gene body regions at four candidate genes (*GND2, LOH1, GRX3, RPL8A*) with promoters replaced with ARS1 or *ars1*, using the primer pairs as illustrated in the cartoon (right). A region on ChrIV which is known to be devoid of ORC and MCM signal serves as the negative loci for MCM signal intensity. Error bars indicate mean values with SD. Unpaired t-test. Source Data are provided as Source Data File.

a

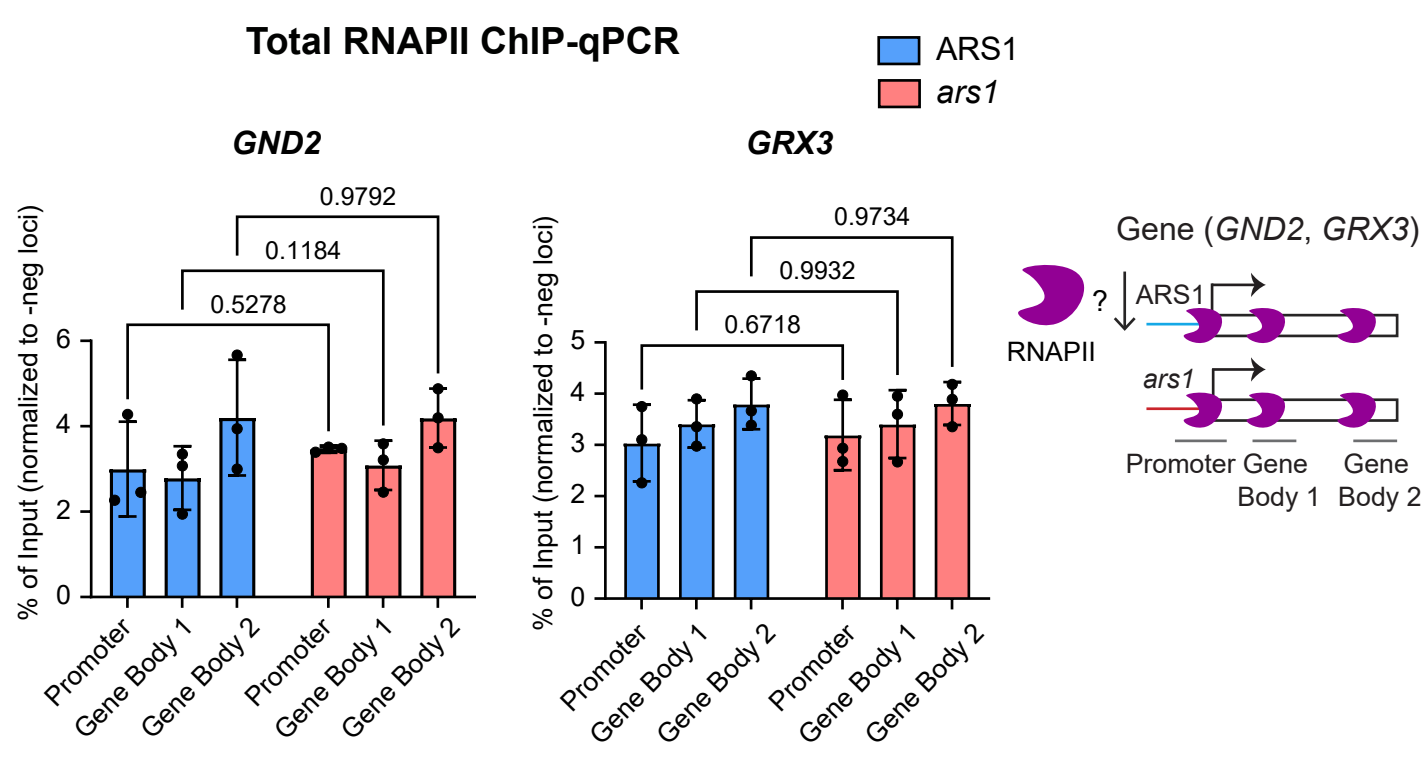

Supplementary Fig. 5.

a. ChIP-qPCR analysis showing total RNAPII levels at the promoter and genic region of two candidate genes (*GND2*, *GRX3*) where the promoters were replaced with ARS1 or *ars1* sequence, using primer pairs as illustrated in the cartoon (right panel). Error bars indicate mean values with standard deviations (SD) (n=3). Unpaired t-test. Source Data are provided as a Source Data File.

Supplementary Tables

Experimental model systems

Yeast strains	Supplementary Data 1	N/A

Bacterial and Virus Strains

REAGENT or RESOURCE	SOURCE	IDENTIFIER
Escherichia coli DH5alpha	Thermo Fisher Scientific	Cat#18265017

Antibodies Antibodies

Antibody Name	SOURCE	IDENTIFIER
Monoclonal mouse IgM anti RNA polymerase II RPB1 (H5)	BioLegend	Cat#920204
Mouse monoclonal [5E6/2] to PCNA	Abcam	Cat#ab70472
Mouse Monoclonal Anti-RNA Polymerase II CTD Antibody (8WG16)	Merck	Cat#05-952; RRID: AB_11213782
Mouse Monoclonal anti-c-MYC clone 9E10	Sigma-Aldrich	Cat# 11667149001 ;
Anti-HA-Peroxidase, high affinity (3F10)	Sigma-Aldrich	Cat#12013819001
Anti-HA High Affinity (3F10); from rat IgG1	Roche	Cat#11867423001
Recombinant Anti-Histone H2A (phospho S129) antibody [EPR17588] - ChIP Grade (ab181447)	Abcam	Cat# ab181447
Polyclonal rabbit anti Histone H3	Abcam	Cat#ab1791; RRID: AB_302613
Goat Anti-Rabbit IgG (H+L), Horseradish peroxidase conjugate	Invitrogen	Cat#G21234; RRID: AB_2536530
Goat Anti-mouse IgG (H+L), Horseradish peroxidase conjugate	Invitrogen	Cat#G21040; RRID: AB_2536527
Polyclonal rabbit anti GFP	Abcam	Cat# ab290
Monoclonal mouse anti RNA-DNA hybrid, clone S9.6	Merck	Cat#SMABE1095
Anti S9.6 monoclonal antibody	Kerafast	Cat#ENH001; RRID: AB_2687463
Anti-mouse peroxidase-conjugated antibody	Jackson ImmunoResearch	Cat#115-035-068; RRID:AB_2338505
Anti-double-stranded DNA antibody	Santa Cruz Biotechnology	Cat#sc-58749; RRID:AB_783088

Chemicals and recombinant proteins

REAGENT OR RESOURCE	SOURCE	IDENTIFIER
5-Ethynyl-2'-deoxyuridine (EdU)	Carl Roth	Cat#7845.3
RNase A	Thermo Scientific	Cat#EN0531
DNase I	New England Biolabs	Cat# M0303S
Proteinase K	SERVA	Cat#33756
phenol:chlorophorm:isoamyl alcohol (25:24:1; v/v)	Invitrogen	Cat# 15593-031
OmniPur Phenol:Chloroform:Isoamyl Alcohol, 25:24:1	Millipore	Cat#6805-100ML
Zymolyase 20T	BioMol	Cat#
Estrogen E2 (β-Estradiol)	Sigma-Aldrich	Cat# E2758
Dynabeads Protein A	Invitrogen	Cat# 10001D
Halt Protease-Inhibitor-Cocktail (100x)	Thermo Scientific	Cat#78429
Halt Protease and Phosphatase Inhibitor Cocktail (100x)	Thermo Scientific	Cat# 78446
SYBR green I nuclei acid dye & SYBR green II RNA gel stain	Thermo Fisher Scientific	Cat# S32717
Terminator 5-Phosphate-Dependent Exonuclease	Lucigen	Cat# TER51020
RNAClean XP	Beckman Coulter	Cat# A63987
4′,6-Diamidino-2-phenylindole dihydrochloride (DAPI)	Sigma Aldrich	Cat# 32670
AlexaFluor 594 azide	Thermo Fisher Scientific	Cat# A10270
AlexaFluor 488 azide	Thermo Fisher Scientific	Cat# A10266
Ascorbic acid	Sigma-Aldrich	Cat#A7506-100G
BSA Fraction V	Sigma-Aldrich	Cat#10735078001
Hydroxyurea (HU)	Biomol	Cat#H9120.10
5M Betain	Thermo Fisher Scientific	Cat# AAJ77507UCR
KAPA HiFi HotStart ReadyMix	Roche	Cat# KK2601
SuperScript II Reverse Transcriptase	Thermo Fisher Scientific	Cat#18064014
Tween-20	Kraft	Cat#21440.2000
Triton X-100	Sigma-Aldrich	Cat#X100-100ML
Deoxycholic acid	Santa Cruz	Cat#sc-214865A
Micrococcal Nuclease	NEB	Cat# M0247S

Critical commercial assays

REAGENT or RESOURCE	SOURCE	IDENTIFIER

SuperSignal™ West Pico PLUS Chemiluminescent Substrate	Thermo Fisher	Cat#34580
iTaq Universal SYBR Green Supermix	Bio-Rad	Cat#1725121
Proteinase K	SERVA	Cat#33756
SuperScript III First-Strand Synthesis System	Thermo Fisher Scientific	Cat#18080-051
Invitrogen™ Click-iT™ EdU Cell Proliferation Kit for Imaging, Alexa Fluor 488 dye	Invitrogen	Cat#C10337
Invitrogen™ Click-iT™ EdU Cell Proliferation Kit for Imaging, Alexa Fluor 594 dye	Invitrogen	Cat#C10339
QIAseq Stranded Total RNA Lib Kit with QIAseq beads	Qiagen	Cat#180745
Duolink detection reagents (ligation/amplification)- Green	Sigma Aldrich	Cat# DUO92014
Duolink *In Situ* PLA Probe Anti-Mouse PLUS	Sigma Aldrich	Cat#DUO92001
Duolink *In Situ* PLA Probe Anti-Rabbit PLUS	Sigma Aldrich	Cat#DUO92002
Duolink *In Situ* PLA Probe Anti-Mouse MINUS	Sigma-Aldrich	Cat#DUO92004
Duolink *In Situ* PLA Probe Anti-Rabbit MINUS	Sigma Aldrich	Cat#DUO92005
KAPA library Quant Kit	Roche (Illumina)	Cat#07960298001
RNeasy Micro Kit	QIAGEN	Cat#74004
Qubit 1X dsDNA HS Kit	Thermo Fisher Scientific	Cat#Q33230

Oligonucleotides

Primers for ChIP-qPCR	Supplementary Data 2	N/A
Primers for STRIPE-seq	Supplementary Data 2	N/A
Primers for RT-qPCR	Supplementary Data 2	N/A
Primers for DNA copy number assay	Supplementary Data 2	N/A
Primers for cloning	Supplementary Data 2	N/A
Primers for yeast strain construction	Supplementary Data 2	N/A
Oligos for yeast strain construction	Supplementary Data 2	N/A

Recombinant DNA

REAGENT or RESOURCE	SOURCE	IDENTIFIER
Plasmid constructs	Supplementary Data 3	N/A

Software and algorithms

REAGENT or RESOURCE	SOURCE	IDENTIFIER
ImageJ	NIH	https://imagej.net/ij/index.html
FlowJo (version 10)	BD Bioscience	
kallisto (v0.48)	[52]	https://github.com/pachterlab/kallisto
MultiQC (v1.25.2)	[47]	https://doi.org/10.1093/bioinformatics/btw354
fastp (v0.24.0)	[48]	https://doi.org/10.1093/bioinformatics/bty560
bedtools (version 2.31.0)	[53]	https://github.com/arq5x/bedtools2/releases/tag/v2.31.0
Salmon (v1.10.3)	[49]	https://doi.org/10.1093/nar/gkae1071
tximeta (v1.24.0)	R Core Team	https://github.com/thelovelab/tximeta
R v4.1	R Core Team	https://www.r-project.org
R (v4.4.3)	R Core Team	https://www.r-project.org
ggplot2 (v3.5.2)	[54]	https://ggplot2.tidyverse.org
clusterProfiler (v4.14)	[50]	https://guangchuangyu.github.io/software/clusterProfiler/
deeptools (version 3.5.2)	[55]	https://deeptools.readthedocs.io/en/develop/index.html
DESeq2 (v1.46.0)	[56]	https://bioconductor.org/packages/release/bioc/html/DESeq2.html
STAR aligner (version 2.7.5a)	[57]	https://github.com/alexdobin/STAR
RSEM (version 1.3.3)	[58]	https://deweylab.github.io/RSEM/ releases
Trimmomatic (v0.39)	[59]	https://github.com/usadellab/Trimmomatic/releases
Tagdust (v2.33)	[60]	https://fredhutch.github.io/easybuild-life-sciences/updates/2020-10-30-tagdust/
UMI-tools (v1.1.2)	[61]	https://umi-tools.readthedocs.io/en/latest/
cutadapt (v4.0)	DOI:10.14806/ej.17.1.200	https://cutadapt.readthedocs.io/en/v4.0/
STAR (v2.7)	[57]	https://github.com/alexdobin/STAR
samtools (v1.15)	htslib.org	https://www.htslib.org/doc/1.15/samtools.html
ACDC	[19]	https://github.com/Schmoller/Cell_ACDC

52. Bray, N.L., Pimentel, H., Melsted, P. & Pachter, L. Near-optimal probabilistic RNA-seq quantification. *Nat Biotechnol* **34**, 525-527 (2016).
53. Quinlan, A.R. & Hall, I.M. BEDTools: a flexible suite of utilities for comparing genomic features. *Bioinformatics* **26**, 841-842 (2010).
54. Wickham, H. in Use R!,, Edn. 2nd 1 online resource (XVI, 260 pages 232 illustrations, 140 illustrations in color (Springer International Publishing : Imprint: Springer,, Cham; 2016).
55. Ramirez, F. *et al.* deepTools2: a next generation web server for deep-sequencing data analysis. *Nucleic Acids Res* **44**, W160-165 (2016).
56. Love, M.I., Huber, W. & Anders, S. Moderated estimation of fold change and dispersion for RNA-seq data with DESeq2. *Genome Biol* **15**, 550 (2014).
57. Dobin, A. *et al.* STAR: ultrafast universal RNA-seq aligner. *Bioinformatics* **29**, 15-21 (2013).
58. Li, B. & Dewey, C.N. RSEM: accurate transcript quantification from RNA-Seq data with or without a reference genome. *BMC Bioinformatics* **12**, 323 (2011).
59. Bolger, A.M., Lohse, M. & Usadel, B. Trimmomatic: a flexible trimmer for Illumina sequence data. *Bioinformatics* **30**, 2114-2120 (2014).
60. Lassmann, T., Hayashizaki, Y. & Daub, C.O. TagDust--a program to eliminate artifacts from next generation sequencing data. *Bioinformatics* **25**, 2839-2840 (2009).
61. Smith, T., Heger, A. & Sudbery, I. UMI-tools: modeling sequencing errors in Unique Molecular Identifiers to improve quantification accuracy. *Genome Res* **27**, 491-499 (2017).
