# Supplementary material for: Humanized yeast ORC drives R-loop dependent transcription-replication conflicts and global gene dysregulation
Source: Nat Commun. 2026 Nov 13;17:11679. doi: 10.1038/s41467-026-79167-4 (PMC13768333; doi:10.1038/s41467-026-79167-4)
Supplement: Supplementary file 2 [file 41467_2026_79167_MOESM2_ESM.pdf]

**Description of Additional Supplementary Files**

File Name: Supplementary Data 1
Description: Yeast strains used in this study.

File Name: Supplementary Data 2
Description: Primers used in this study.

File Name: Supplementary Data 3
Description: Plasmids used in this study.
