# Supplementary material for: Humanized yeast ORC drives R-loop dependent transcription-replication conflicts and global gene dysregulation
Source: Nat Commun. 2026 Nov 13;17:11679. doi: 10.1038/s41467-026-79167-4 (PMC13768333; doi:10.1038/s41467-026-79167-4)
Supplement: Supplementary file 3 [file 41467_2026_79167_MOESM6_ESM.pdf]

nature portfolio

Corresponding author(s): Stephan Hamperl

Last updated by author(s): Aug 16, 2026

Reporting Summary

Nature Portfolio wishes to improve the reproducibility of the work that we publish. This form provides structure for consistency and transparency in reporting. For further information on Nature Portfolio policies, see our Editorial Policies and the Editorial Policy Checklist.

Statistics

For all statistical analyses, confirm that the following items are present in the figure legend, table legend, main text, or Methods section.

n/a	Confirmed	
☐	☒	The exact sample size (*n*) for each experimental group/condition, given as a discrete number and unit of measurement
☐	☒	A statement on whether measurements were taken from distinct samples or whether the same sample was measured repeatedly
☐	☒	The statistical test(s) used AND whether they are one- or two-sided<br>*Only common tests should be described solely by name; describe more complex techniques in the Methods section.*
☐	☒	A description of all covariates tested
☐	☒	A description of any assumptions or corrections, such as tests of normality and adjustment for multiple comparisons
☐	☒	A full description of the statistical parameters including central tendency (e.g. means) or other basic estimates (e.g. regression coefficient) AND variation (e.g. standard deviation) or associated estimates of uncertainty (e.g. confidence intervals)
☐	☒	For null hypothesis testing, the test statistic (e.g. *F*, *t*, *r*) with confidence intervals, effect sizes, degrees of freedom and *P* value noted<br>*Give P values as exact values whenever suitable.*
☒	☐	For Bayesian analysis, information on the choice of priors and Markov chain Monte Carlo settings
☒	☐	For hierarchical and complex designs, identification of the appropriate level for tests and full reporting of outcomes
☐	☒	Estimates of effect sizes (e.g. Cohen's *d*, Pearson's *r*), indicating how they were calculated

Our web collection on statistics for biologists contains articles on many of the points above.

Software and code

Policy information about availability of computer code

Data collection	Date were collected as described in the Methods - Section Bioinformatic Analysis
Data analysis	Data were analyzed using the software tools as described in the Methods - Section Tables

For manuscripts utilizing custom algorithms or software that are central to the research but not yet described in published literature, software must be made available to editors and reviewers. We strongly encourage code deposition in a community repository (e.g. GitHub). See the Nature Portfolio guidelines for submitting code & software for further information.

Data

Policy information about availability of data

All manuscripts must include a data availability statement. This statement should provide the following information, where applicable:

- Accession codes, unique identifiers, or web links for publicly available datasets
- A description of any restrictions on data availability
- For clinical datasets or third party data, please ensure that the statement adheres to our policy

This paper does not report any original code.
Any additional information required to reanalyze the data reported in this paper is available from the lead contact upon request.
The data of time course RNA-Seq, STRIPE-Seq, RNA-Seq in Wt and Orc4-IH⍰ with RNH1 overexpression, MNase-Seq with candidate gene promoter sequence replaced with nucleosome-forming sequence, and MNase-Seq with candidate gene promoter replaced with origin sequence, have been deposited to European

Nucleotide Archive (ENA) under accession numbers PRJEB98985, PRJEB100524, PRJEB123404, PRJEB123431, and PRJEB123527, respectively.

Research involving human participants, their data, or biological material

Policy information about studies with human participants or human data. See also policy information about sex, gender (identity/presentation), and sexual orientation and race, ethnicity and racism.

Reporting on sex and gender	This paper does not report research involving human participants
Reporting on race, ethnicity, or other socially relevant groupings	This paper does not report research involving human participants
Population characteristics	This paper does not report research involving human participants
Recruitment	This paper does not report research involving human participants
Ethics oversight	This paper does not report research involving human participants

Note that full information on the approval of the study protocol must also be provided in the manuscript.

Field-specific reporting

Please select the one below that is the best fit for your research. If you are not sure, read the appropriate sections before making your selection.

☒ Life sciences ☐ Behavioural & social sciences ☐ Ecological, evolutionary & environmental sciences

For a reference copy of the document with all sections, see nature.com/documents/nr-reporting-summary-flat.pdf

Life sciences study design

All studies must disclose on these points even when the disclosure is negative.

Sample size	No sample size calculation were performed, the authors consider the use of n=3 biological replicates sufficient for all research work in Saccharomyces cerevisiae
Data exclusions	No data were excluded from the analysis. All data have been generated using standard yeast genetics and cell biology.
Replication	All key experimental findings reported in this study were independently replicated. No experimental findings central to the conclusions of this study failed to replicate.
Randomization	Randomization is not relevant to this study given the used standar molecular biology, yeast genetics and NGS readouts.
Blinding	Blinding is not relevant to this study given the used standard molecular biology, yeast genetics and NGS readouts.

Reporting for specific materials, systems and methods

We require information from authors about some types of materials, experimental systems and methods used in many studies. Here, indicate whether each material, system or method listed is relevant to your study. If you are not sure if a list item applies to your research, read the appropriate section before selecting a response.

Materials & experimental systems

n/a	Involved in the study	
☐	☒	Antibodies
☐	☒	Eukaryotic cell lines
☒	☐	Palaeontology and archaeology
☒	☐	Animals and other organisms
☒	☐	Clinical data
☒	☐	Dual use research of concern
☒	☐	Plants

Methods

n/a	Involved in the study	
☒	☐	ChIP-seq
☒	☐	Flow cytometry
☒	☐	MRI-based neuroimaging

Antibodies

Antibodies used	All antibodies used are described in the Methods - Section Tables
Validation	All primary antibodies represent commercially purchased antibodies and were not further validated for specificity.

Eukaryotic cell lines

Policy information about cell lines and Sex and Gender in Research

Cell line source(s)	A summary of all yeast strains with corresponding genotypes are listed in Supplementary Table 1
Authentication	All yeast strains were verified by genotyping PCR and Sanger sequencing.
Mycoplasma contamination	Yeast (Sacharomyces cerevisiae) is not susceptible to Mycoplasma contamination
Commonly misidentified lines (See ICLAC register)	Not used in this study

Plants

Seed stocks	Not used in this study
Novel plant genotypes	Not used in this study
Authentication	Not used in this study
