# Supplementary material for: Humanized yeast ORC drives R-loop dependent transcription-replication conflicts and global gene dysregulation
Source: Nat Commun. 2026 Nov 13;17:11679. doi: 10.1038/s41467-026-79167-4 (PMC13768333; doi:10.1038/s41467-026-79167-4)
Supplement: Supplementary file 4 [file 41467_2026_79167_MOESM7_ESM.pdf]

Peer Review File

Humanized yeast ORC drives R-loop dependent transcription-replication conflicts and global gene dysregulation

Corresponding Author: Dr Stephan Hamperl

This file contains all reviewer reports in order by version, followed by all author rebuttals in order by version.

Version 0:

Reviewer comments:

Reviewer #1

(Remarks to the Author)
ORC binding sites in higher eukaryotic chromosomes are enriched at promoters, raising fundamental questions about how DNA replication may interfere with gene transcription. The authors address this using the yeast Orc4-IHΔ mutant, which recapitulates the human ORC binding landscape. They show that ORC binding at promoters deregulates transcription during the cell cycle, triggers conflicts between replication and transcription, and induces R-loop–dependent replication stress, highlighting the potential risk of transcription replication conflicts at promoter-associated ORC sites. The study also demonstrates that ARS sequences possess intrinsic promoter activity. Overall, the work provides important insights and establishes Orc4-IHΔ as a valuable tool to study questions related to DNA replication and chromosome stability.
Please find below my remarks:

1-The authors previously reported, in an earlier study, that mutant Orc4-IHΔ did not exhibit any delay in the replication process. However, in the present study, the authors appear to demonstrate the opposite, namely that the mutant displays an extended DNA replication phase compared to the wild-type strain. It is therefore important to clarify this apparent discrepancy.
2-The authors suggest that the replication delay is due to RNA–DNA hybrids induced by transcription–replication conflicts. This claim is supported by spot assays showing that suppression of RNase H rescues the growth defects of the strain (Figure 1F). I believe it would be preferable to show the original, uncropped images of these spot assays rather than pre-cropped images, as this would allow a better assessment of the results.
3-The authors propose that the accumulation of cells in G2 is due to RNA–DNA hybrids, since RNase H overexpression reduces the proportion of cells arrested in G2 in an asynchronous population. However, the effect shown in the figure is very subtle. To better assess the effect of RNase H overexpression on the replication defect associated with RNA–DNA hybrids, it would be more appropriate to perform the analysis using synchronized cultures, as in Figure 1C
4- As DNA replication is tightly linked to sister chromatid cohesion, the accumulation of Orc4-IHΔ cells in G2 could be due to a defect in sister chromatid cohesion. Could the authors therefore analyze the recruitment of cohesin subunits or directly assess sister chromatid cohesion in this mutant?
5- Figure 3B. How can the enrichment of ORC over gene bodies, rather than promoters, of highly transcribed genes in wild-type cells be explained?
6- I suggest that the authors consider applying a more quantitative method, such as calibrated ChIP-seq, in selected experiments to more accurately measure Pol II, MCM, and ORC occupancy on DNA.

Reviewer #2

(Remarks to the Author)
Evaluation Summary
The idea of using a mutant yeast model that reproduces human ORC binding patterns to investigate effects on transcription is highly original and, considering the experimental value of yeast, deserves attention. The conclusion that Transcription-Replication Conflict (TRC) result from formation of R loops needs to be validated at a few exemplary genes.

Overview
Previous studies from these authors demonstrated that mutant yeast ORC (Orc4-IHΔ) preferentially relocalizes from ARS to nucleosome-deficient promoter regions. In this study, they examined the effects on transcription using a mutant yeast model that replicates this human ORC binding pattern. It is very interesting how the mutant ORC re-distributes to promoters, just as seen with mammalian ORC, and that MCM loading is now no longe well correlated with ORC binding, again as seen with mammalian ORC. The finding that ARS1, perhaps from being a nucleosome depleted region, can also act as a promoter is also intriguing, but not well validated.

The main findings and results are as follows:
Orc4-IHΔ cells depend on a functional S-phase checkpoint, particularly in the presence of replication stress. Nonspecific binding of ORC-IHΔ to the promoter in Orc4-IHΔ cells induces TRC, activating the S-phase checkpoint that delays growth and S-phase progression and causes G2/M cell cycle arrest. RNH1 overexpression rescues the growth arrest, suggesting that R-loops are also involved, but it is unclear that R loops are actually fmed. Extensive binding of ORC-IHΔ to the promoter region in Orc4-IHΔ cells inhibits the transcription program throughout the cell cycle. In Orc4-IHΔ cells, binding of ORC-IHΔ to the promoter region induced loading of the MCM complex and redistribution to the gene body, suggesting that this leads to transcriptional repression, particularly in highly transcribed genes.

Replacing the promoter sequence elements with ARS1 and ars1 replication origins maintained transcription initiation but altered the precise selection of the transcription start site, suggesting that they possess intrinsic promoter-like functions. Furthermore, it is suggested that binding of ORC to an ARS1 promoter increases the frequency of transcription start site utilization and transcription initiation frequency, particularly in the case of potent promoters with high levels of transcription product.

This is an excellent paper and the suggestions below are primarily to help improve the paper. The authors may choose to simply rebut our questions, or perhaps remove some of the weaker data/conclusions from the paper.

Major point 1
While it is suggested that non-specific binding of ORC-IHΔ to the promoter in Orc4-IHΔ cells induces TRC through R-loops, there is insufficient evidence that R loops actually form or that the R-loop formation explains the changes in gene expression. Are R-loops actually formed in the promoter regions or bodies of the four genes they selectively study (GND2, LOH1, GRX3, and RPL8A) in Orc4-IHΔ cells, and is there any relationship between their formation and whether gene expression increases or decreases?
In relation to the above, it would be better to show the locations of the four genes mentioned above in Figure 3c and Extended Data Figure 3b.

Major point 2
Do any of the promoters that were replaced with ARS elements contain a functional TATA box? If they do, what provides the TATA box like function in ARS1 or ars1?
Why is there more transcription from ars1 compared to ARS1? The authors suggest that this is because ORC or MCM do not bind to ars1, but can that be shown by inactivating ORC or MCM using conditional mutants to evaluate whether that increases transcription from these sites?
The authors suggest that the nucleosome depleted region of ARS1 (or ars1) is sufficient for promoter activity. Can that be experimentally demonstrated by deleting the nucleosome depleted region of ARS1 in one of the promoters?

Major point 3
Regarding the Pearson correlation analysis between the expression levels of differentially expressed genes and the binding strength of ORC and MCM to their promoter regions and gene bodies, the correlation coefficients alone show little to no correlation (Fig. 3c, ORC, Extended Data Fig. 3) or a very weak correlation (Fig. 3c, MCM G2M). If this data is published, it would be good to also show the p-value.
Based on this data, it is difficult to conclude that the redistributed ORC/MCM complexes in Orc4-IHΔ cells tend to more strongly repress gene expression during late S phase. Also, it is not clear why their effect will be more pronounced in late S phase. This is not a major point of the paper and may not need to be shown.

Minor points:
1) The description of Extended Data Fig. 2e is insufficient.

2) Regarding the RNA-seq data, it would be best to specify the number of replicates (presumably two?).

3) Regarding the changes in gene expression levels shown in Figure 5a, were the sequences of the nucleosome-free regions in the promoters deleted when the ARS1/ars1 were inserted? If so, it would be helpful to also show the promoter activity under those conditions.

Reviewer #3

(Remarks to the Author)
In this manuscript by Lee et al., the authors report several interesting findings surrounding the impact of a “humanized” yeast ORC that lost its ability to recognize the typical yeast ARS consensus sequence. Two main findings appear. First, this mutant ORC that preferentially localizes to promoter NFR regions triggers a broad change in gene expression accompanied

by a significant cell cycle block in G2/M and evidence for increased proximity between the transcription and replication machineries indicative of elevated conflicts between these two processes. In a second part, the authors replace several endogenous promoters by a typical ARS sequence and report the surprising finding that ARSs associate with significant transcription initiation activity. Thus, a mutant ORC no longer able to bind to typical ARS sequences localizes to promoters and an ARS sequence functions as a promoter, highlighting interesting similarities between promoters and ARSs.

Unfortunately, the two parts appear disjointed and, on their own, each part is relatively incomplete. Thus, while the manuscript (which is well-written) starts strong, one leaves disappointed by the end.

Major comments:
- Authors show convincingly that the ORC-IH mutant induces a cell cycle delay / block without obvious elevated DNA damage. They attribute this to transcription-replication conflicts (TRCs) based on evidence of higher PLA signal between RNAPII and PCNA. Such TRCs were further ascribed to increased R-loops on the evidence that RNase H1 overexpression can rescue the cell cycle defect. However, the authors did not measure R-loops in the study, either in WT or ORC mutant cells, and did not identify which R-loops were allegedly removed by RNase H1 to resolve the conflicts. As the authors are undoubtedly aware. RNase H1 has been implicated in the processing of post-replicative hybrids in yeast and of Replication Fork associated hybrids in mammalian cells. It is therefore quite unclear if a standard R-loop model is correct and at any rate, this hasn't been tested. At this point, all that should be said is the "ORC-IH binding at promoters in ORC-IH cells provoke TRCs that can be suppressed by RNase H1 activity" (line 179). A lot more digging is required to implicate R-loops, including R-loop mapping in WT and mutant strains, identifying which R-loops are affected by RNase H1, and proving beyond correlations that these R-loops caused TRCs.
- The description of transcriptional rewiring by the ORC-IH mutant is fairly straightforward and convincing. The authors try to pin this major rewiring as a secondary consequence of TRCs and S-phase checkpoint activation. This section is relatively weak and unconvincing, as per the authors own admission. It clearly seems to me that a dedicated cellular stress response is being triggered by the ORC-IH mutant. Given the GOs and the seeming collapse of rRNA production, authors should specifically investigate what is happening over the rDNA regions and follow that aspect. While pinning the blame on TRCs might be seen as necessary to keep "the story", I don't think it is doing the actual biology at play any service…
- The section dealing with the notion that transcription might be pushing MCM complexes towards gene bodies in the ORC-IH mutant and repress transcription is again relatively weak and unconvincing.
- The next part, dealing now with replacing specific promoter sequences with the ARS1 sequence, appears disjointed from the rest. The section reads fine but raises many questions. Why for instance, does the same ARS1 function as a strong promoter at 3 tested loci but not so much at the fourth (GRX3)? At GRX3, the introduction of the ARS1 sequence seems to strongly affect initiation at neighboring gene promoters (Ext Fig 5b); how is that explained? A similar phenomenon is seen at LOH1, with the appearance of strong initiation signals 1.5 kb from the insertion point. It seems that at 3 out of 4 tested loci, the mutant ars1 sequence has stronger initiation activity than the ARS1 sequence capable of recruiting pre-RCs, but not at RPL8A – why?
- Perhaps one of the key takeaways from this last part is that ARS1surprisingly possesses promoter activity. But the generality of this conclusion can't be established because only one ARS was tested. I would strongly suggest to test other ARS to determine whether promoter function is a conserved feature.

Minor comments:
- Authors argue that the lack of difference between ARS1 and ars1 in replication initiation at LOH1 is due to the proximity with a nearby efficient origin. Authors should test this and delete ARS1012.
- There is a Typo in Figure 5a x-axis (relese)

Version 1:

Reviewer comments:

Reviewer #1

(Remarks to the Author)
I appreciate the effort the authors have made to address my comments. Nevertheless, regarding point 1, I think the authors should mention in the text the reasons that may explain the discrepancy between the two studies.

Reviewer #2

(Remarks to the Author)
The authors have satisfactory answered my comments. I liked the paper in the first round itself, and it is even better now.

Reviewer #3

(Remarks to the Author)
My comments have been successfully addressed.

Open Access This Peer Review File is licensed under a Creative Commons Attribution 4.0 International License, which permits use, sharing, adaptation, distribution and reproduction in any medium or format, as long as you give appropriate credit to the original author(s) and the source, provide a link to the Creative Commons license, and indicate if changes were made.
In cases where reviewers are anonymous, credit should be given to 'Anonymous Referee' and the source.
The images or other third party material in this Peer Review File are included in the article's Creative Commons license, unless indicated otherwise in a credit line to the material. If material is not included in the article's Creative Commons license and your intended use is not permitted by statutory regulation or exceeds the permitted use, you will need to obtain permission directly from the copyright holder.
To view a copy of this license, visit https://creativecommons.org/licenses/by/4.0/

Reviewer #1

ORC binding sites in higher eukaryotic chromosomes are enriched at promoters, raising fundamental questions about how DNA replication may interfere with gene transcription. The authors address this using the yeast Orc4-IHΔ mutant, which recapitulates the human ORC binding landscape. They show that ORC binding at promoters deregulates transcription during the cell cycle, triggers conflicts between replication and transcription, and induces R-loop–dependent replication stress, highlighting the potential risk of transcription replication conflicts at promoter-associated ORC sites. The study also demonstrates that ARS sequences possess intrinsic promoter activity. Overall, the work provides important insights and establishes Orc4-IHΔ as a valuable tool to study questions related to DNA replication and chromosome stability.

We sincerely thank the reviewer for their positive assessment and acknowledgment of the significance of our study. We are pleased that the reviewer found our work provides important insights into transcription-replication conflicts and establishes the Orc4-IHΔ mutant as a valuable tool for studying chromosome stability. We greatly appreciate the reviewer's constructive feedback and have addressed all specific comments below to further improve the manuscript.

Please find below my remarks:

1-The authors previously reported, in an earlier study, that mutant Orc4-IHΔ did not exhibit any delay in the replication process. However, in the present study, the authors appear to demonstrate the opposite, namely that the mutant displays an extended DNA replication phase compared to the wild-type strain. It is therefore important to clarify this apparent discrepancy.

2-The authors suggest that the replication delay is due to RNA–DNA hybrids induced by transcription–replication conflicts. This claim is supported by spot assays showing that suppression of RNase H rescues the growth defects of the strain (Figure 1F). I believe it would be preferable to show the original, uncropped images of these spot assays rather than pre-cropped images, as this would allow a better assessment of the results.

We thank the reviewer for pointing out this apparent discrepancy, and we appreciate the opportunity to clarify our findings.

The reviewer is entirely correct that in our previous study, we did not capture a significant delay in S-phase progression for the Orc4-IHΔ mutant[1]. In that earlier work, cells were synchronized and sampled at 15-minute intervals. Because the S-phase delay in this mutant is relatively brief (approximately 10–15 minutes to reach a similar DNA content), this original time-window resolution unfortunately caused us to miss the subtle shift in replication kinetics.

In the current study, we performed the synchronization experiments with a much higher temporal resolution, collecting samples every 5 minutes (**Figure 1c**). Thanks to this tighter sampling window, we were able to detect the modest 10–15 minute delay in S-phase progression that went unnoticed previously. Importantly, we would like to note

that the subsequent delay in the G2/M phase is fully and consistently reproduced between both our previous study and the current manuscript.

We also provide below the uncropped images of the original spot assay in **Figure 1 for Reviewer** to allow better assessment of the results but we would prefer to keep the cropped images in the main figure for space consideration. We agree that the rescue of the growth defect is more subtle and difficult to assess from the spot assays. Therefore we now additionally performed growth curve assays in Wt and Orc4-IHΔ mutant cells with and without RNH1 overexpression which showed a clear rescue of the cell cycle progression phenotype (**New Figure 1h**).

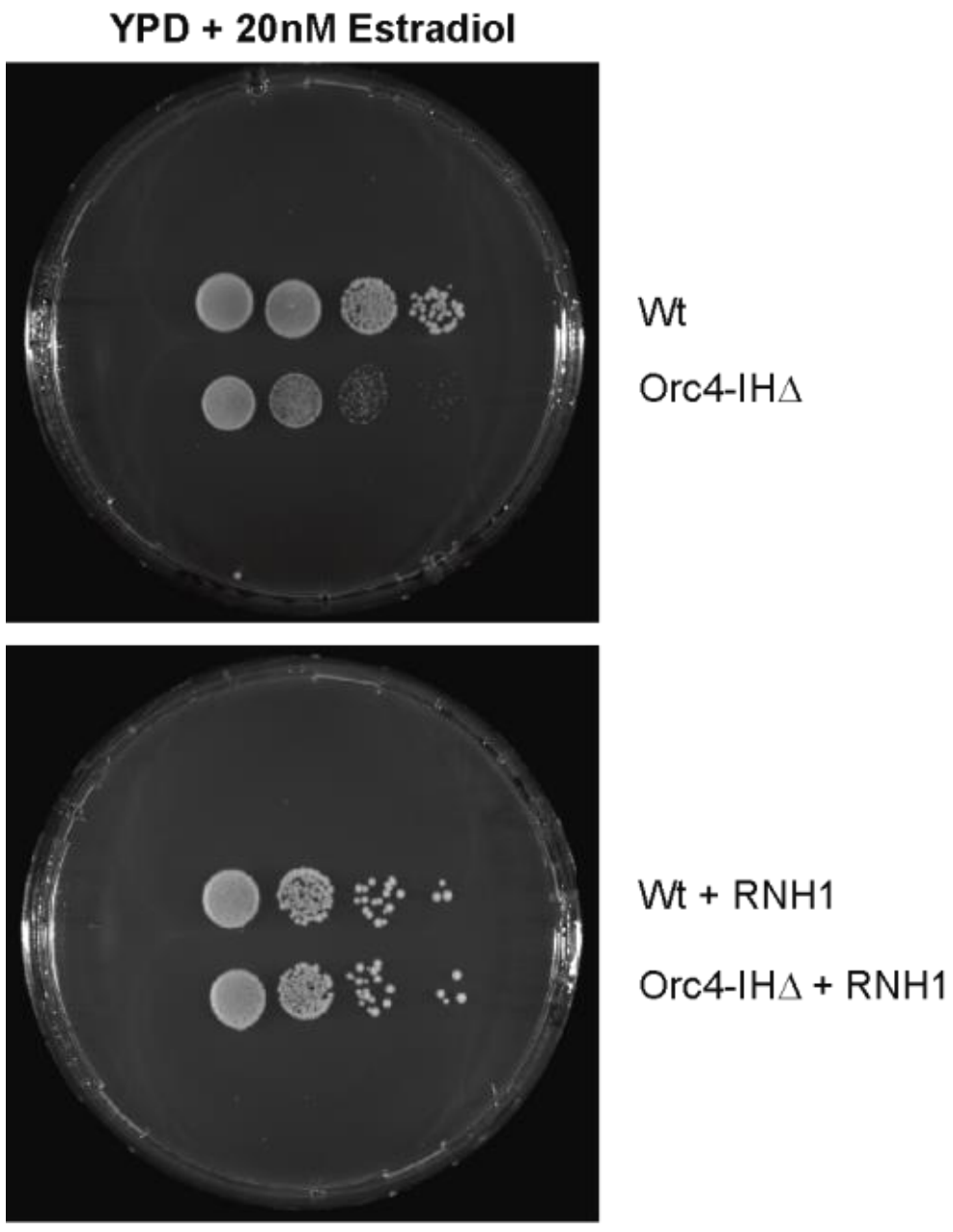

Figure 1 for Reviewer: Uncropped Images of the spot assay of Wt and Orc4-IHΔ mutant cells with and without RNH1 overexpression.

3-The authors propose that the accumulation of cells in G2 is due to RNA–DNA hybrids, since RNase H overexpression reduces the proportion of cells arrested in G2 in an asynchronous population. However, the effect shown in the figure is very subtle. To better assess the effect of RNase H overexpression on the replication defect associated with RNA–DNA hybrids, it would be more appropriate to perform the analysis using synchronized cultures, as in Figure 1C

We appreciate the reviewer's constructive suggestion to assess the effect of RNase H overexpression using synchronized cultures. Besides the growth curve analysis in Wt and Orc4-IHΔ mutant cells with and without RNH1 overexpression (see comment 2 above), we also aimed to directly link RNA:DNA hybrid accumulation to the specific cell cycle defect in S-phase progression and G2/M accumulation. To this end, we measured global R-loop levels directly using a dot blot assay within distinct cell populations synchronized in the G1, early S, and G2/M phases of the cell cycle. Through this approach (now presented as a new **Figure 1f** in the revised manuscript), we observed a clear, preferential accumulation of R-loops specifically in G2 cells in the Orc4-IHΔ mutant, which aligns perfectly with the timing of the observed cell cycle delay.

4- As DNA replication is tightly linked to sister chromatid cohesion, the accumulation of Orc4-IHΔ cells in G2 could be due to a defect in sister chromatid cohesion. Could the authors therefore analyze the recruitment of cohesin subunits or directly assess sister chromatid cohesion in this mutant?

We thank the reviewer for this intriguing suggestion. The reviewer is entirely correct that DNA replication stress is intimately linked to sister chromatid cohesion, and defects in cohesion can indeed trigger the observed G2/M checkpoint arrest.

While exploring the potential impact of cohesin dynamics in our mutant is a highly interesting avenue for future research, we feel it falls outside the immediate scope of the current study. The primary focus of this work is to characterize how promoter-associated ORC binding induces transcription-replication conflicts and drives R-loop-dependent replication stress and transcriptional rewiring.

Because we observe a direct, cell cycle phase-specific accumulation of R-loops in G2/M cells (as discussed in Comment 3) and a rescue effect upon RNase H overexpression (Comment 2), we believe our current data provides a strong, self-contained mechanism showing that R-loop-induced replication stress is a primary driver of the G2/M delay.

To address the reviewer's excellent point, we have added a section to the Discussion (**page 20, lines 550-554**) acknowledging that post-replicative defects, such as altered sister chromatid cohesion, could potentially contribute to or exacerbate this G2/M accumulation, representing an interesting direction for future studies.

5- Figure 3B. How can the enrichment of ORC over gene bodies, rather than promoters, of highly transcribed genes in wild-type cells be explained?

We agree with the reviewer that the enrichment of ORC over the gene bodies of highly transcribed genes in wild-type cells is an intriguing and highly interesting observation.

While we do not yet have a definitive mechanistic explanation for this specific pattern, we favor a model where a phenomenon similar to what occurs globally in the Orc4-IHΔ mutant may be happening locally at these highly expressed loci in wild-type cells. Specifically, we speculate that while ORC may initially bind near the promoter regions of these genes, the high density of actively transcribing RNA Polymerase II (RNAPII) complexes physically pushes or translocates the bound ORC (or the licensed MCM single hexamers) along the gene body. This transcription-driven "pushing" of the replication machinery has been documented in other contexts (e.g., [2–4]). Alternatively, this subset of highly transcribed genes may contain a more “open” promoter that could facilitate a certain level of baseline ORC binding with lower affinity but could be sufficiently long to recruit MCMs. In wild-type cells, this likely occurs at a much lower frequency or is restricted to exceptionally highly transcribed loci, whereas the Orc4-IHΔ mutant exacerbates and generalizes this conflict across the genome.

We fully agree that the precise molecular orchestration of this process warrants deeper investigation. To address the reviewer's comment and stimulate further discussion, we have added a paragraph to the revised Discussion (**page 21, lines 578-583**) outlining this potential RNAPII-mediated pushing mechanism to explain the wild-type gene body enrichment.

6- I suggest that the authors consider applying a more quantitative method, such as calibrated ChIP-seq, in selected experiments to more accurately measure Pol II, MCM, and ORC occupancy on DNA.

We thank the reviewer for this suggestion. The genome-wide ChIP-seq data in this study derive from our previous work (Reference), which did not include spike-in calibration, and repeating these experiments with calibrated ChIP-seq was beyond the scope of the current study. As an alternative quantitative approach for the key loci (LOH1, GND2, GRX3 and RPL8A) underlying our core findings and for which new Pol II, ORC or MCM ChIP analysis were performed as part of this study, we performed input-normalized ChIP-qPCR, which corrects for variability in immunoprecipitation efficiency and DNA recovery at the locus level. We have expanded the Methods section (**page 32-33, lines 906-914**) to detail the normalization formulas and internal controls used.

Reviewer #2

Evaluation Summary

The idea of using a mutant yeast model that reproduces human ORC binding patterns to investigate effects on transcription is highly original and, considering the experimental value of yeast, deserves attention. The conclusion that Transcription-Replication Conflict (TRC) result from formation of R loops needs to be validated at a few exemplary genes.

Overview

Previous studies from these authors demonstrated that mutant yeast ORC (Orc4-IHΔ) preferentially relocalizes from ARS to nucleosome-deficient promoter regions. In this study, they examined the effects on transcription using a mutant yeast model that replicates this human ORC binding pattern. It is very interesting how the mutant ORC re-distributes to promoters, just as seen with mammalian ORC, and that MCM loading is now no longe well correlated with ORC binding, again as seen with mammalian ORC. The finding that ARS1, perhaps from being a nucleosome depleted region, can also act as a promoter is also intriguing, but not well validated.

The main findings and results are as follows:

Orc4-IHΔ cells depend on a functional S-phase checkpoint, particularly in the presence of replication stress. Nonspecific binding of ORC-IHΔ to the promoter in Orc4-IHΔ cells induces TRC, activating the S-phase checkpoint that delays growth and S-phase progression and causes G2/M cell cycle arrest. RNH1 overexpression rescues the growth arrest, suggesting that R-loops are also involved, but it is unclear that R loops are actually fmed. Extensive binding of ORC-IHΔ to the promoter region in Orc4-IHΔ cells inhibits the transcription program throughout the cell cycle. In Orc4-IHΔ cells, binding of ORC-IHΔ to the promoter region induced loading of the MCM complex and redistribution to the gene body, suggesting that this leads to transcriptional repression, particularly in highly transcribed genes.

Replacing the promoter sequence elements with ARS1 and ars1 replication origins maintained transcription initiation but altered the precise selection of the transcription start site, suggesting that they possess intrinsic promoter-like functions. Furthermore, it is suggested that binding of ORC to an ARS1 promoter increases the frequency of transcription start site utilization and transcription initiation frequency, particularly in the case of potent promoters with high levels of transcription product.

This is an excellent paper and the suggestions below are primarily to help improve the paper. The authors may choose to simply rebut our questions, or perhaps remove some of the weaker data/conclusions from the paper.

We sincerely thank the reviewer(s) for their constructive, insightful, and highly encouraging feedback. We are grateful for their characterization of our manuscript as an "excellent paper" and their appreciation of our Orc4-IHΔ mutant yeast model as "highly original" for recapitulating mammalian-like ORC binding and MCM loading patterns. Below, we provide a point-by-point response to the specific suggestions and questions raised.

Major point 1

While it is suggested that non-specific binding of ORC-IHΔ to the promoter in Orc4-IHΔ cells induces TRC through R-loops, there is insufficient evidence that R loops actually form or that the R-loop formation explains the changes in gene expression. Are R-loops actually formed in the promoter regions or bodies of the four genes they selectively study (GND2, LOH1, GRX3, and RPL8A) in Orc4-IHΔ cells, and is there any relationship between their formation and whether gene expression increases or decreases?

In relation to the above, it would be better to show the locations of the four genes mentioned above in Figure 3c and Extended Data Figure 3b.

We entirely agree that direct, physical validation of R-loop formation at these specific loci is critical to substantiate our model. To address this major point, we have performed two independent, complementary experiments: 1) a global cell-cycle-resolved S9.6 dot blot analysis and 2) locus-specific DRIP-qPCR across the requested four candidate genes.

The dot blot analysis was performed on synchronized cell populations isolated at G1, S, and G2/M phases of the cell cycle. Intriguingly, we observe the major accumulation of R-loops in G2/M cells (see New Figure 1f), which argues that the TRCs occurring during S-phase lead to persistent, unresolved RNA:DNA hybrids that trigger the S-phase checkpoint, ultimately resulting in the observed G2/M cell cycle arrest. This temporal pattern highlights that these aberrant nucleic acid structures are not rapidly cleared during replication but instead accumulate and persist into the post-replicative cell cycle phases.

Furthermore, we also performed S9.6 DRIP-qPCR at the four candidate genes (*GND2*, *LOH1*, *GRX3*, and *RPL8A*) as requested by the reviewer. For each gene, we observe a significant accumulation of R-loops in in Orc4-IHΔ cells compared to wildtype cells at both promoter and gene body regions (New Figure 3f), suggesting that ectopic ORC binding and subsequent aberrant MCM loading lead to subsequent R-loop

stabilization. This result is particularly interesting in the light of our previous results that two of the candidate genes are upregulated (*LOH1, GND2*) and the other two (*GRX3 and RPL8A*) are downregulated in our gene expression datasets (see **Figure 3e**). Thus, the accumulation of R-loops appears to be independent of the direction of gene expression changes. A plausible explanation is that the physical barrier imposed by ectopic ORC/MCM complexes triggers TRCs and alters RNA polymerase II dynamics in a locus-specific manner. In the case of the downregulated genes (*GRX3, RPL8A*), the stabilized R-loops may act as severe physical roadblocks that block transcriptional elongation, resulting in a drop in mature mRNA levels. Conversely, for the upregulated genes (*LOH1, GND2*), the accumulation of R-loops may induce a state of higher local chromatin accessibility, that may trigger more frequent transcription initiation events. The exact molecular explanation will require additional mechanistic studies out of the scope of this manuscript. Independent of whether the steady-state transcript levels ultimately increase or decrease, the universal presence of these RNA:DNA hybrids demonstrate that mislocalized licensing complexes inherently destabilize the transcription program by disrupting normal elongation homeostasis across all investigated gene loci.

Finally, as suggested by the reviewer, we have updated the Pearson Correlation plots (now **Extended Data Figure 3c**) to explicitly indicate the locations of these four key representative genes (*GND2, LOH1, GRX3, and RPL8A*).

Major point 2

Do any of the promoters that were replaced with ARS elements contain a functional TATA box? If they do, what provides the TATA box like function in ARS1 or ars1?

Why is there more transcription from ars1 compared to ARS1? The authors suggest that this is because ORC or MCM do not bind to ars1, but can that be shown by inactivating ORC or MCM using conditional mutants to evaluate whether that increases transcription from these sites?

The authors suggest that the nucleosome depleted region of ARS1 (or ars1) is sufficient for promoter activity. Can that be experimentally demonstrated by deleting the nucleosome depleted region of ARS1 in one of the promoters?

We thank the reviewer for these intriguing questions regarding the underlying mechanism of the promoter-like activity of ARS elements.

To address the first point concerning transcription initiation motifs, it is important to note that not all promoters in yeast rely on a functional TATA box; a significant proportion of the *S. cerevisiae* genome utilizes TATA-less promoters that rely on alternative core promoter elements or specific chromatin architectures to recruit the transcription machinery[5]. Therefore, the promoter activity of ARS may also be achieved by a TATA-box-less mechanism, as observed for many other native yeast promoters. Nevertheless, we have screened the sequences of these regions to determine if they contain potential TATA-box motifs. We found that promoters of *GND2, GRX3, and LOH1* contain TATA-box motifs. There are no TATA-box motifs found in the ARS1 and

ars1 sequences. However, we identified multiple transcription factor binding motifs for Abf1 and Sok2.

Regarding why ars1 induces higher levels of transcription compared to ARS1, this observation aligns well with the fact that the ars1 mutation abolishes the sequence-specific recruitment and stable binding of ORC. Crucially, ORC binding at yeast loci has long been established to exert a potent repressive function on local gene expression, both through direct transcriptional interference and by recruiting structural silencing factors[6,7]. Consequently, removing the stable assembly of the ORC/MCM complexes on the ars1 element effectively relieves this repressive constraint, allowing for heightened transcription initiation efficiency from the exposed origin region.

While evaluating transcription from these sites under conditional origin-licensing mutants remains an interesting avenue for future research, we instead chose to rigorously test the sequence specificity and structural requirements of the ARS1 promoter-like function using an alternative genetic strategy. Specifically, we performed a new set of experiments where we replaced the endogenous promoters of the four candidate genes not with ARS1 or ars1, but instead with either a randomized sequence containing the exact same base composition as ARS1, or with a synthetic nucleosome-forming sequence[8] to further test our hypothesis whether the causal link to the promoter activity is the formation of a nucleosome-depleted region.

Intriguingly, both of these new control sequences completely abolished gene expression (Updated **Figure 5d**). In addition, MNase-Seq confirmed the formation of a nucleosome-free region for ARS1, but not for the synthetic nucleosome-forming sequence (Updated **Figure 5e**). Because the randomized sequence preserved the AT-rich character of ARS1 but lacked the capacity to form a strong nucleosome-depleted region, its failure to drive transcription demonstrates that a simple AT-rich sequence is not sufficient on its own. Furthermore, the failure of the synthetic nucleosome-forming sequence to support expression confirms that forcing a closed chromatin state effectively kills promoter activity at these loci. Together, these new data provide strong, definitive evidence that the promoter activity we observe is highly specific to the specific sequence layout of the ARS1 element itself rather than being an artifact of non-specific sequence composition or passive physical spacing, substantially validating our original conclusions.

Major point 3

Regarding the Pearson correlation analysis between the expression levels of differentially expressed genes and the binding strength of ORC and MCM to their promoter regions and gene bodies, the correlation coefficients alone show little to no correlation (Fig. 3c, ORC, Extended Data Fig. 3) or a very weak correlation (Fig. 3c, MCM G2M). If this data is published, it would be good to also show the p-value.

Based on this data, it is difficult to conclude that the redistributed ORC/MCM complexes in Orc4-IHΔ cells tend to more strongly repress gene expression during late S phase. Also, it is not clear why their effect will be more pronounced in late S phase. This is not a major point of the paper and may not need to be shown.

We acknowledge the reviewer's point regarding the correlation analysis and agree that the Pearson correlation coefficients in these datasets are indeed low. As requested,

we have now included the p-values for all correlation analyses now shown in **Extended Data Figure 3c**. While some of these correlations reach statistical significance due to the large number of genes analyzed, we agree that the low correlation values indicate that the binding strength of redistributed ORC/MCM complexes is not the sole determinant of the magnitude of gene expression changes.

In light of this, we have significantly toned down our conclusions regarding the strength and timing of this repression. We have revised the text to clarify that while there is a detectable global trend, the impact of ORC/MCM redistribution on transcription is likely modulated by local chromatin context, baseline transcription rates, and the specific orientation of TRCs at individual loci. Furthermore, we have tempered our claims regarding a more pronounced effect specifically in late S phase.

While the correlation is indeed weak, we have chosen to retain this data in the manuscript for the sake of transparency and completeness as it provides a global overview of the relationship between licensing protein redistribution and the transcriptome. However, we removed it from the main figure to the supporting extended data figure and adjusted the discussion to ensure that these results are presented as a subtle global trend rather than a primary driver of the Orc4-IHΔ phenotype. We believe this more cautious interpretation accurately reflects the complexity of the genomic data while remaining focused on the more robust findings of our study.

Minor points:

1) The description of Extended Data Fig. 2e is insufficient.

We appreciate this feedback and apologize for the lack of detail in the original manuscript submission. We have thoroughly expanded the description of this Figure in the Methods section to explain the details of the performed GO term clustering analysis.

2) Regarding the RNA-seq data, it would be best to specify the number of replicates (presumably two?).

We have updated the manuscript to clearly state the number of biological replicates used for the RNA-seq analysis. The reviewer is correct that this experiment was performed using two independent biological replicates per condition ($n = 2$). We have explicitly inserted this information into both the main text methods section and the corresponding figure legends to ensure complete transparency regarding our sample sizes.

3) Regarding the changes in gene expression levels shown in Figure 5a, were the sequences of the nucleosome-free regions in the promoters deleted when the ARS1/ars1 were inserted? If so, it would be helpful to also show the promoter activity under those conditions.

We apologize for the lack of clarity in our previous manuscript submission. Indeed, we have directly replaced the nucleosome-free regions in the promoters with the nucleosome-depleted ARS1/ars1 sequences and therefore did not construct strains where the nucleosome-free region was deleted only. Nevertheless, as mentioned in our response to major point 2, we have now additionally replaced the endogenous promoter with either a randomized sequence of identical base composition or a

synthetic nucleosome-forming sequence, essentially creating the control conditions suggested by the reviewer. Because both of these replacement strategies completely abolished gene expression (Updated Figure 5d), these results effectively demonstrate that the deletion of the native nucleosome-free promoter elements results in zero baseline transcription, further emphasizing that the promoter activity observed in Figure 5a is uniquely driven by the specific sequence layout of the inserted ARS1/ars1 elements.

Reviewer #3

In this manuscript by Lee et al., the authors report several interesting findings surrounding the impact of a "humanized" yeast ORC that lost its ability to recognize the typical yeast ARS consensus sequence. Two main findings appear. First, this mutant ORC that preferentially localizes to promoter NFR regions triggers a broad change in gene expression accompanied by a significant cell cycle block in G2/M and evidence for increased proximity between the transcription and replication machineries indicative of elevated conflicts between these two processes. In a second part, the authors replace several endogenous promoters by a typical ARS sequence and report the surprising finding that ARSs associate with significant transcription initiation activity. Thus, a mutant ORC no longer able to bind to typical ARS sequences localizes to promoters and an ARS sequence functions as a promoter, highlighting interesting similarities between promoters and ARSs.

Unfortunately, the two parts appear disjointed and, on their own, each part is relatively incomplete. Thus, while the manuscript (which is well-written) starts strong, one leaves disappointed by the end.

We thank the reviewer for their positive assessment of our manuscript, highlighting our two main findings regarding the functional similarities between replication origins and transcriptional promoters.

We appreciate the reviewer's constructive critique regarding the perceived disconnect between the two main parts of the manuscript and the missing depth in each section. We took this feedback very seriously during the revision process. To address these concerns, we have now included a series of new experimental data to bridge the two sections.

Specifically, the revised manuscript now includes:

- We performed cell-cycle-resolved S9.6 slot blots and locus-specific DRIP-qPCR at candidate genes to physically substantiate the involvement of R-loops in our TRC model.
- We replaced the ARS promoter-substitution sequences with both scrambled random controls and synthetic nucleosome-positioning sequences to dissect the sequence- versus structure-dependence of origin-mediated transcription.
- We generated new MNase-Seq profiles in these substitution strains, establishing that the capacity of an ARS to drive transcription is fundamentally dependent on its intrinsic ability to maintain a nucleosome-depleted region (NDR).
- We utilized time-resolved Proximity Ligation Assays (PLA) between ORC and MCM subunits to track the physical displacement of MCM single hexamers

away from their ORC loading sites by the transcription machinery during G1 phase.

We believe these revisions have significantly consolidated the manuscript, transforming it into a cohesive, comprehensive study. Below, we address the reviewer's specific comments point-by-point.

Major comments:

- Authors show convincingly that the ORC-IHΔ mutant induces a cell cycle delay / block without obvious elevated DNA damage. They attribute this to transcription-replication conflicts (TRCs) based on evidence of higher PLA signal between RNAPII and PCNA. Such TRCs were further ascribed to increased R-loops on the evidence that RNase H1 overexpression can rescue the cell cycle defect. However, the authors did not measure R-loops in the study, either in WT or ORC mutant cells, and did not identify which R-loops were allegedly removed by RNase H1 to resolve the conflicts. As the authors are undoubtedly aware. RNase H1 has been implicated in the processing of post-replicative hybrids in yeast and of Replication Fork associated hybrids in mammalian cells. It is therefore quite unclear if a standard R-loop model is correct and at any rate, this hasn't been tested. At this point, all that should be said is the "ORC-IHΔ binding at promoters in ORC-IHΔ cells provoke TRCs that can be suppressed by RNase H1 activity" (line 179). A lot more digging is required to implicate R-loops, including R-loop mapping in WT and mutant strains, identifying which R-loops are affected by RNase H1, and proving beyond correlations that these R-loops caused TRCs.

We thank the reviewer for this constructive comment and completely agree that direct, physical validation of R-loop formation is essential to substantiate our model and rule out alternative interpretations regarding post-replicative or fork-associated hybrids. To address this, we have performed two independent, complementary measurements of R-loop formation in both wildtype (WT) and Orc4-IHΔ strains, including a global cell-cycle-resolved S9.6 slot blot analysis and a locus-specific DNA-RNA Hybrid Immunoprecipitation (DRIP-qPCR) across four key candidate gene loci.

To determine if R-loops are globally elevated and to understand their cell-cycle timing in Orc4-IHΔ cells, we performed a global cell-cycle-resolved S9.6 slot blot analysis on synchronized cell populations isolated at G1, S, and G2/M phases (New **Figure 1f**). Intriguingly, we observed a major, significant accumulation of R-loops specifically in G2/M-phase Orc4-IHΔ cells, which argues that the TRCs occurring during S-phase lead to persistent, unresolved RNA:DNA hybrids that trigger the S-phase checkpoint, ultimately resulting in the observed G2/M cell cycle arrest. This temporal pattern highlights that these aberrant nucleic acid structures are not rapidly cleared during replication but instead accumulate and persist into the post-replicative cell cycle phases.

To provide locus-specific resolution beyond global correlations, we performed S9.6 DRIP-qPCR at four representative candidate genes (*GND2, LOH1, GRX3, and RPL8A*) as now shown in New **Figure 3f**. For all four genes, we observed a significant and reproducible accumulation of R-loops in Orc4-IHΔ cells compared to WT controls at both promoter and gene body regions. This directly demonstrates that ectopic ORC

binding and subsequent aberrant MCM loading physically lead to downstream R-loop stabilization at these genomic loci. We have now updated the manuscript to integrate and discuss these findings.

Finally, following the reviewer's recommendation, we have revised the language throughout the relevant section (including the sentence at the previous line 179 of the original manuscript) to more accurately reflect that R-loops suppressible by RNase H1 are implicated in these TRCs, rather than definitively established as their cause.

- The description of transcriptional rewiring by the ORC-IHΔ mutant is fairly straightforward and convincing. The authors try to pin this major rewiring as a secondary consequence of TRCs and S-phase checkpoint activation. This section is relatively weak and unconvincing, as per the authors own admission. It clearly seems to me that a dedicated cellular stress response is being triggered by the ORC-IHΔ mutant. Given the GOs and the seeming collapse of rRNA production, authors should specifically investigate what is happening over the rDNA regions and follow that aspect. While pinning the blame on TRCs might be seen as necessary to keep "the story", I don't think it is doing the actual biology at play any service…

We thank the reviewer for this insightful critique. The reviewer is entirely correct that a robust, dedicated cellular stress response is being triggered in the Orc4-IHΔ mutant, and we agree that attributing this massive transcriptional rewiring solely to a passive downstream consequence of S-phase checkpoint activation oversimplified the complex biology at play.

To address this and better define the molecular trigger of this stress response, we performed an RNA-seq on wildtype and Orc4-IHΔ cells with and without RNH1 overexpression across three biological replicates. Strikingly, RNase H1 overexpression was able to rescue to a large part (up to 90%) the transcriptional rewiring observed in the Orc4-IHΔ mutant (New **Figures 3h, i, Extended Data Fig. 3g, h**). Notably, individual replicates exhibited varying degrees of RNH1 induction, with Replicate 1(R1) showing the highest, Replicate 2 (R2) intermediate, and Replicate 3 (R3) the lowest RNH1 overexpression levels (Extended Data Fig. 3h). The observed rescue correlated with the level of RNH1 overexpression, strongly suggesting that the widespread transcriptional changes are, at least in part, mechanistically driven by R-loop accumulation and active TRCs, rather than being a non-specific or disjointed secondary effect.

By integrating these new RNase H1 overexpression RNA-Seq data, we believe that the revised manuscript provides now additional and substantial evidence for our initial model that the Orc4-IHΔ mutant induces severe, R-loop-mediated TRCs, which subsequently trigger a robust, dedicated cellular stress response that rewires the transcriptome. We believe these additions significantly strengthen this section and do justice to the actual biology occurring in these cells.

Regarding the reviewer's excellent point concerning the potential collapse of rRNA production and defects at rRNA genes: we closely analyzed our RNA expression data to see if rRNA production is a primary hotspot that drives secondary gene expression changes. Unfortunately, our analysis could not identify a clear trend as throughout the different cell cycle stages, Replicate 1 showed no effective changes in rRNA levels

between Wt and Orc4-IHΔ cells, whereas Replicate 2 showed a small increase in rRNA levels in Orc4-IHΔ cells (see **Figure 2 for Reviewer** below).

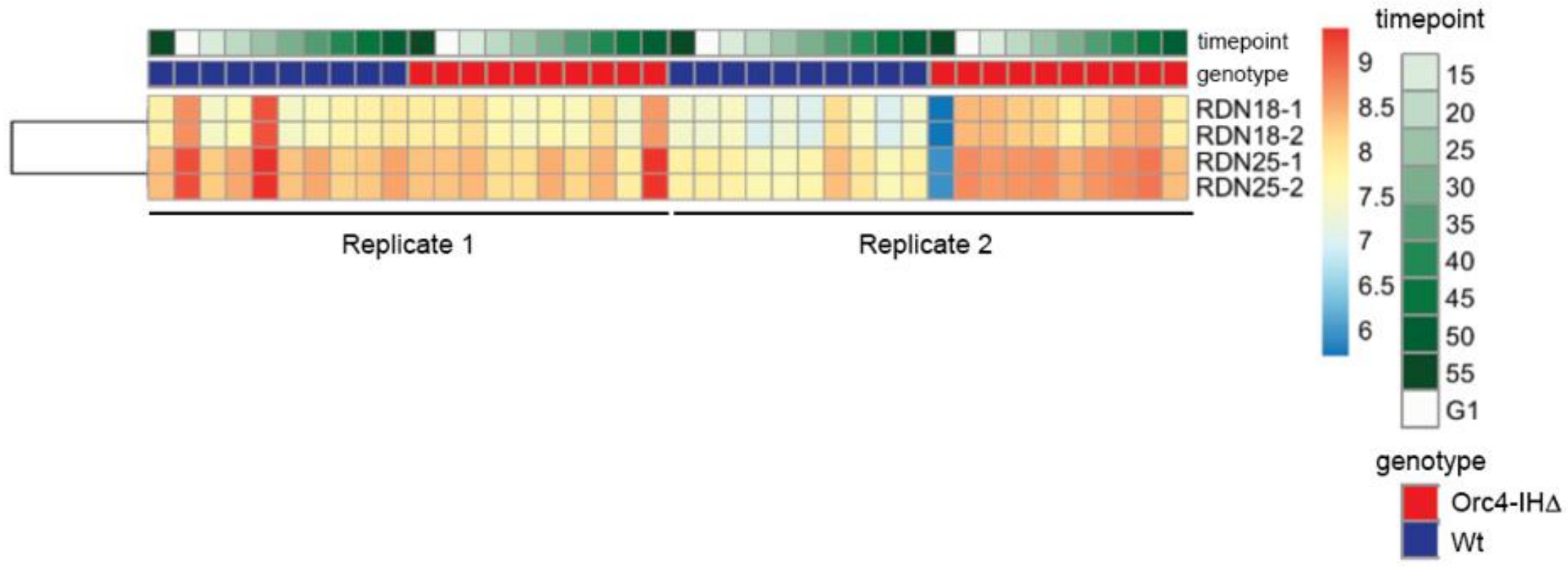

Figure 2 for Reviewer: RNA-Seq analysis of Wt and Orc4-IHΔ cells of 18S (RDN18-1/2) and 25S (RDN25S1-2) ribosomal RNA expression levels of the two biological replicates. Please note the non-coherent pattern of expression levels between Replicate 1 and Replicate 2.

- The section dealing with the notion that transcription might be pushing MCM complexes towards gene bodies in the ORC-IHΔ mutant and repress transcription is again relatively weak and unconvincing.

We appreciate the reviewer's critical assessment of this specific mechanistic aspect of our model and agree that our initial conclusion, that transcription forces the relocation of MCM complexes into gene bodies within the Orc4-IHΔ mutant, required more direct physical evidence to be fully convincing. To directly test this hypothesis and strengthen this section, we have tracked the spatial proximity and dynamics of ORC and MCM complexes across different cell cycle stages. Specifically, we performed Proximity Ligation Assays (PLA) between ORC and MCM subunits in synchronized Wt and Orc4-IHΔ mutant cell populations isolated during both early G1 and late G1 phases.

Our model predicts that while ORC initially loads MCM complexes at ectopic promoter sites in early G1, active transcription subsequently pushes these loaded MCM single hexamers away from their original deposition sites and into downstream gene bodies as cells progress toward the G1/S transition. In full agreement with this hypothesis, our new PLA data show that In Wt cells, ORC-MCM proximity remained stable throughout G1. In contrast, Orc4-IHΔ cells rather tended to decrease their ORC-MCM PLA signal as cells transitioned from early to late G1 phase (New **Fig. 2c, Extended Data Fig. 2b**). This physical uncoupling indicates that while mutant ORC initially loads MCM complexes at non-canonical promoter sites in early G1, active transcription subsequently displaces these loaded single hexamers away from ORC and into downstream gene bodies before replication begins, providing an independent experimental validation for our transcription-driven sliding model. We have now integrated these new PLA quantification data as a new panel in the revised manuscript and updated the main text to strengthen this section.

- The next part, dealing now with replacing specific promoter sequences with the ARS1 sequence, appears disjointed from the rest. The section reads fine but raises many questions. Why for instance, does the same ARS1 function as a strong promoter at 3

tested loci but not so much at the fourth (GRX3)? At GRX3, the introduction of the ARS1 sequence seems to strongly affect initiation at neighboring gene promoters (Ext Fig 5b); how is that explained? A similar phenomenon is seen at LOH1, with the appearance of strong initiation signals 1.5 kb from the insertion point. It seems that at 3 out of 4 tested loci, the mutant ars1 sequence has stronger initiation activity than the ARS1 sequence capable of recruiting pre-RCs, but not at RPL8A – why?

- Perhaps one of the key takeaways from this last part is that ARS1 surprisingly possesses promoter activity. But the generality of this conclusion can't be established because only one ARS was tested. I would strongly suggest to test other ARS to determine whether promoter function is a conserved feature.

We entirely agree with the reviewer that our initial observation, that ARS1 possesses promoter activity, opened up several intriguing questions regarding both its mechanistic basis and its generality. To rigorously address these points and disentangle the underlying rules of this origin promoter activity, we have performed extensive follow-up experiments using new mutant strains coupled with MNase-Seq analysis. These new findings have now been integrated into the revised manuscript, which we believe provides the missing mechanistic depth the reviewer rightly asked for.

First, to address whether this promoter activity is simply a consequence of inserting any AT-rich sequence, we replaced the ARS1 sequence at our reporter loci with a shuffled random sequence of the exact same base composition. Strikingly, this shuffled sequence completely abolished promoter activity, demonstrating that random AT-rich sequences alone are insufficient to drive transcription (see new **Figure 5d**) Second, we replaced the ARS1 sequence with a synthetic sequence engineered to strongly position nucleosomes[8]. This modification also completely killed promoter activity (see new **Figure 5d**).

To understand the molecular basis of these observations, we performed MNase-Seq on these new strains to map the local nucleosome occupancy under these conditions. The results show that both the shuffled random sequence and the synthetic positioning sequence result in the formation of stable nucleosomes that effectively close the promoter region (see new **Figure 5e**). Conversely, the wild-type ARS1 inherently maintains a robust nucleosome-depleted region (NDR) (see new **Figure 5a and 5e**). Taken together, these new experiments allow us to conclude that the key, conserved feature enabling an ARS to drive gene expression is its intrinsic ability to form and maintain an NDR, which subsequently allows the pre-initiation complex (PIC) to assemble and initiate transcription.

This mechanism also directly explains the locus-to-locus variability highlighted by the reviewer, including the altered initiation dynamics at GRX3 and the emergence of distal initiation signals at LOH1. To directly test this, we performed STRIPE-seq to map TSSs genome-wide and overlaid these data with our MNase-seq datasets, allowing us to simultaneously track TSS selection and +1 nucleosome positioning at each locus (new **Fig. 6a–d**). This revealed a general principle: at every locus tested, origin substitution repositions the +1 nucleosome, and TSS selection shifts concordantly to track this new nucleosome boundary, frequently uncovering cryptic initiation sites that are normally suppressed by native chromatin architecture. At GRX3 specifically, MNase-seq

showed that promoter replacement shifts the boundary of the nucleosome-depleted region, repositioning the -1 and +2 nucleosomes downstream and thereby exposing cryptic, internal TSSs within the GRX3 coding sequence (**Figure 6d**). Similarly, at LOH1, both ARS1 and ars1 replacement strains lost the native TSS entirely; instead, the shifted +1 nucleosome closely tracked the appearance of new, highly active cryptic TSSs located both near the start codon and further into the coding region, consistent with the distal initiation signals noted by the reviewer (**Figure 6b**). Notably, overall initiation frequency at LOH1 was elevated in both origin-substituted strains relative to wild type, in line with their increased steady-state mRNA levels (**Figure 5a**). Together, these co-localized STRIPE-seq and MNase-seq datasets establish a chromatin-centered model in which origin-mediated nucleosome-depleted regions drive transcription initiation by setting new local boundaries for pre-initiation complex assembly, with the precise choice of TSS at each locus dictated by +1 nucleosome positioning.

Regarding the reviewer's excellent point about testing other ARS elements to establish the generality of this phenomenon: while we did not replace the promoter with an entirely different, independent ARS locus, we believe our new control experiments successfully establish the underlying molecular rules governing this behavior. To address the broader context of this finding, we examined our endogenous STRIPE-Seq data and found that approximately 10% of native ARS elements are associated with or located in close proximity to a transcription start site (TSS) (**Figure 4c**). This suggests that while widespread promoter activity may not be a universal baseline feature of all ARS sequences within their native genomic contexts, a significant subset does naturally possess this capacity.

Nevertheless, it remains highly possible that transferring other ARS elements out of their endogenous contexts and into a different genomic location would result in the establishment of a transcription-competent NDR. Fully exploring this positional plasticity will be an important avenue for future research. We have revised the text to explicitly clarify this distinction, highlighting that the capacity to drive transcription is fundamentally linked to the conserved, NDR-forming nature of these loci.

Minor comments:

- Authors argue that the lack of difference between ARS1 and ars1 in replication initiation at LOH1 is due to the proximity with a nearby efficient origin. Authors should test this and delete ARS1012.

We thank the reviewer for this constructive suggestion to test our hypothesis regarding the proximity of ARS1012. While we agree that deleting ARS1012 would definitively determine its influence on replication initiation at the modified *LOH1* locus, we feel that pursuing this line of experimentation falls outside the primary scope of our current study, which focuses on the transcriptional capabilities of these loci. Because this point does not alter the main conclusions of the manuscript, we have instead revised the text to more carefully frame this explanation as a likely possibility rather than a definitive claim.

- There is a Typo in Figure 5a x-axis (relese)

We thank the reviewer for spotting this mistake and have now corrected the x axis labeling.

References:

1. Lee, C. S. K. *et al.* Humanizing the yeast origin recognition complex. *Nat Commun* **12**, 33 (2021).
2. Chen, Y.-H. *et al.* Transcription shapes DNA replication initiation and termination in human cells. *Nat Struct Mol Biol* **26**, 67–77 (2019).
3. Gros, J., Devbhandari, S. & Remus, D. Origin plasticity during budding yeast DNA replication in vitro. *EMBO J* **33**, 621–636 (2014).
4. Scherr, M. J., Wahab, S. A., Remus, D. & Duderstadt, K. E. Mobile origin-licensing factors confer resistance to conflicts with RNA polymerase. *Cell Rep* **38**, 110531 (2022).
5. Cairns, B. R. The logic of chromatin architecture and remodelling at promoters. *Nature* **461**, 193–198 (2009).
6. Triolo, T. & Sternglanz, R. Role of interactions between the origin recognition complex and SIR1 in transcriptional silencing. *Nature* **381**, 251–253 (1996).
7. Foss, M., McNally, F. J., Laurenson, P. & Rine, J. Origin recognition complex (ORC) in transcriptional silencing and DNA replication in S. cerevisiae. *Science* **262**, 1838–1844 (1993).
8. Routhier, E. *et al.* In silico design of DNA sequences for in vivo nucleosome positioning. *Nucleic Acids Res* **52**, 6802–6810 (2024).
